# Long noncoding RNA SH3PXD2A-AS1 promotes NSCLC proliferation and accelerates cell cycle progression by interacting with DHX9

**DOI:** 10.1038/s41420-022-01004-6

**Published:** 2022-04-11

**Authors:** Yeqing Zhou, Hongmei Yong, WenJie Cui, Sufang Chu, Minle Li, Zhongwei Li, Jin Bai, Hao Zhang

**Affiliations:** 1 Thoracic Surgery Laboratory, The First College of Clinical Medicine, Xuzhou Medical University, Xuzhou, 221006 Jiangsu Province China; 2 Department of Thoracic Surgery, Affiliated Hospital of Xuzhou Medical University, 99 West Huaihai Road, Xuzhou, 221006 Jiangsu China; 3 Department of Thoracic Surgery, Shengze Hospital in Jiangsu, Suzhou, 215228 Jiangsu China; 4 Department of Oncology, the Affiliated Huai’an Hospital of Xuzhou Medical University and The Second People’s Hospital of Huai’an, Huai’an, Jiangsu China; 5 Department of Respiratory and Critical Care Medicine, The Municipal Hospital Affiliated to Xuzhou Medical University, Xuzhou, Jiangsu China; 6 Cancer Institute, Xuzhou Medical University, Xuzhou, 221006 Jiangsu China; 7 Center of Clinical Oncology, the Affiliated Hospital of Xuzhou Medical University, Xuzhou, 221006 Jiangsu China

**Keywords:** Cancer, Cell growth

## Abstract

As the most commonly diagnosed lung cancer, non–small cell lung carcinoma (NSCLC) is regulated by many long noncoding RNAs (lncRNAs). In the present study, we found that SH3PXD2A-AS1 expression in NSCLC tissues was upregulated compared with that in normal lung tissues in The Cancer Genome Atlas (TCGA) database by using the GEPIA website. K-M analysis was performed to explore the effects of this molecule on the survival rate in NSCLC. The results demonstrated that SH3PXD2A-AS1 expression was increased in human NSCLC, and high SH3PXD2A-AS1 expression was correlated with poor overall survival. SH3PXD2A-AS1 promotes lung cancer cell proliferation and accelerates cell cycle progression in vitro. Animal studies validated that knockdown of SH3PXD2A-AS1 inhibits NSCLC cell proliferation in vivo. Mechanically, SH3PXD2A-AS1 interacted with DHX9 to enhance FOXM1 expression, promote tumour cell proliferation and accelerate cell cycle progression. Altogether, SH3PXD2A-AS1 promoted NSCLC growth by interacting with DHX9 to enhance FOXM1 expression. SH3PXD2A-AS1 may serve as a promising predictive biomarker for the diagnosis and prognosis of patients with NSCLC.

## Introduction

Lung cancer is the most frequent cause of cancer-related death globally [1]. Approximately 85% of all new lung cancer cases are non–small cell lung carcinoma (NSCLC). the issue ofA diagnosis at advanced stages and the propensity for metastasis result in poor outcomes, and the overall 5-year survival rate for the disease is less than 15% [2, 3]. Thus, identification of novel effective biomarkers for early diagnosis, prognosis and therapeutic improvement is urgently needed.

Long noncoding RNAs (lncRNAs) are an important group of transcribed RNA molecules greater than 200 nucleotides in length [4, 5]. LncRNAs participate in multiple biological processes in cancer cells, and the dysregulation of lncRNAs is highly associated with cancer cell proliferation, invasion and metastasisat the transcriptional or post-transcriptional level [6–8]. For example, the long noncoding RNA GMAN, which is upregulated in gastric cancer tissues, is associated with migration and metastasis [9]. LncRNA KTN1-AS1 predicts a poor prognosis and regulates non–small cell lung cancer cell proliferation [10]. LncRNAs may be important regulators of tumorigenesis and proliferation [11–13], but the detailed mechanisms of lncRNAs in lung cancer should be further elucidated. These results prompted us to explore the role of lncRNAs in human NSCLC.

In a preliminary experiment, we assayed SH3PXD2A-AS1 expression through The Cancer Genome Atlas (TCGA) database in NSCLC. We found higher expression in tumour tissues than normal tissues and high expression was associated with a poor prognosis. SH3PXD2A-AS1 is an antisense transcript transcribed from SH3PXD2A and located on chromosome 10 and is 2023 bp in length. SH3PXD2A-AS1 has recently been reported to be upregulated in colorectal cancer tissues and to promote cell proliferation, cell cycle progression, migration and invasion [14, 15]. However, its biological role and specific mechanism in NSCLC should be further uncovered.

To further elucidate the mechanism by which SH3PXD2A-AS1 regulates lung cancer, we identified probable target genes of SH3PXD2A-AS1 in NSCLC. Forkhead box M1 (FOXM1), also known as HNF-3, HFH-11 or Trident, is a transcription factor of the Forkhead box (Fox) protein superfamily that is defined by a conserved winged helix DNA-binding domain1 [16]. FOXM1 is a critical proliferation-associated transcription factor that is widely spatiotemporally expressed during the cell cycle [17]. Centromere protein F (CENPF) is a cell cycle-associated nuclear antigen that is expressed at low levels in G0/G1 cells and accumulates in the nuclear matrix during S phase, with maximal expression in G2/M cells [18]. Kinesin family member 20 A (KIF20A), previously named MKLP2 and RAB6KIFL, is located on chromosome 5q31.2 [19]. KIF20A mainly accumulates in the central region of the mitotic cell spindle and participates in cell mitosis [20].

In this study, we revealed that SH3PXD2A-AS1 could promote NSCLC cell proliferation and accelerate cell cycle progression in vitro and in vivo. SH3PXD2A-AS1 interacted with ATP-dependent RNA helicase A (DHX9) to promote the expression of FOXM1 and further led to NSCLC cell proliferation and cell cycle progression.

## Results

### SH3PXD2A-AS1 is upregulated in NSCLC tumour tissues

**Fig. 1:**
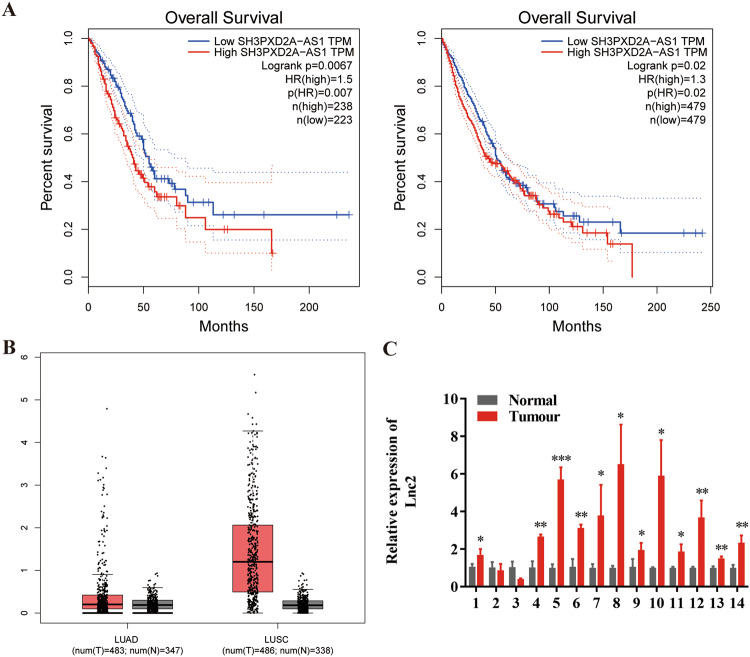
SH3PXD2A-AS1 is upregulated in NSCLC tumour tissues. **A** Kaplan–Meier survival curve analysis was performed to explore the effects of the genes on the survival rate in LUAD (Left, *P* < 0.01) and NSCLC (Right, *P* < 0.05). **B** SH3PXD2A-AS1 expression was analysed in LUAD or LUSC tissues and normal tissues in the GEPIA database. **C** SH3PXD2A-AS1 expression was validated in 14 pairs of NSCLC patient samples by qRT-PCR. Lnc2(SH3PXD2A-AS1). Data are shown as the mean ± standard deviation from three independent experiments. *P < 0.05, **P < 0.01, ***P < 0.001.

Microarray analysis through GEPIA was used to identify differentially expressed SH3PXD2A-AS1 in LUAD or NSCLC tissues and adjacent tissues. SH3PXD2A-AS1 expression in the LUAD or NSCLC tissues was upregulated compared with that in the normal lung tissues (Fig. 1A). In addition, a prognostic analysis of SH3PXD2A-AS1 was performed, and the results demonstrated that high expression of SH3PXD2A-AS1 had an adverse effect on survival, as shown by Kaplan‐Meier analysis (Fig. 1B). Then, we performed qRT-PCR analysis to investigate the SH3PXD2A-AS1 expression level in LUAD patient tissues, which was significantly higher in the tumour tissues than in the corresponding normal tissues (Fig. 1C).

### SH3PXD2A-AS1 promotes lung cancer cell proliferation and accelerates cell cycle progression in vitro

**Fig. 2:**
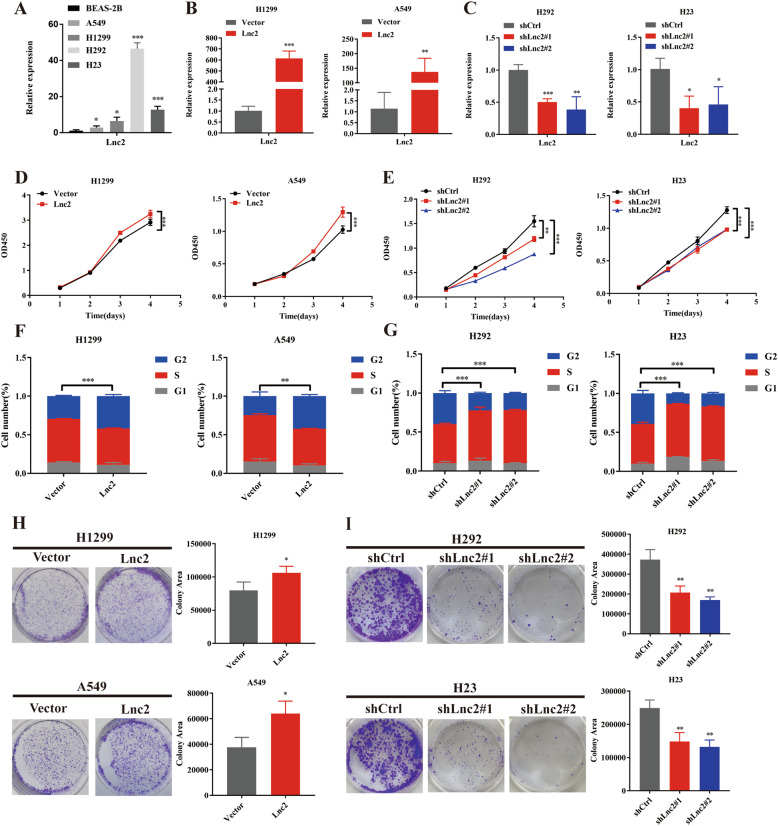
SH3PXD2A-AS1 promoted lung cancer cell proliferation and accelerated cell cycle progression in vitro. **A** The expression level of SH3PXD2A-AS1 was detected by qRT-PCR assays in four NSCLC cell lines (A549, H1299, H292, and H23) and an immortalised normal human lung epithelial cell line (BEAS-2B). **B** Overexpression of SH3PXD2A-AS1 was confirmed at the mRNA level in A549 and H1299 cells by qRT-PCR. **C** Knockdown of SH3PXD2A-AS1 was confirmed at the mRNA level in H292 and H23 cells by qRT-PCR. **D, E** Effect of SH3PXD2A-AS1 OE or KD on A549, H1299, H292 and H23 cell proliferation as assessed by Cell Counting Kit-8 assays. **F, G** The percentage of S/G2 population cells was measured by flow cytometry for SH3PXD2A-AS1 OE or KD A549, H1299, H292 and H23 cell lines. **H, I** Colony formation assays for SH3PXD2A-AS1 OE or KD on A549, H1299, H292 and H23 cell lines. Lnc2(SH3PXD2A-AS1). Data are shown as the mean ± standard deviation from three independent experiments. *P < 0.05, **P < 0.01, ***P < 0.001.

To explore the biological functions of SH3PXD2A-AS1 in lung cancer cells, we first examined SH3PXD2A-AS1 expression in the immortalised normal human lung epithelial cell line BEAS-2B and a series of lung cancer cell lines, including A549, H1299, H292 and H23, by qRT-PCR (Fig. 2A). SH3PXD2A-AS1 expression in the lung cancer cell lines was higher than that in the normal human lung epithelial cell line and was much higher in H292 and H23 cells than the other cells. Then, a full-length recombinant plasmid with SH3PXD2A-AS1 was used to increase SH3PXD2A-AS1 expression in A549 and H1299 cells (Fig. 2B), whereas lentivirus-mediated control shRNA or an effective SH3PXD2A-AS1 shRNA was used to specifically knock down SH3PXD2A-AS1 expression in H292 and H23 cells (Fig. 2C). SH3PXD2A-AS1 expression was substantially up- or downregulated 24 or 48 h after transfection. CCK-8 and colony formation assays revealed that SH3PXD2A-AS1 overexpression promoted cell growth and proliferation of A549 and H1299 cells, while knockdown of SH3PXD2A-AS1 significantly inhibited the growth and proliferation of H292 and H23 cells (Fig. 2D–E, H–I). Then, we further determined whether SH3PXD2A-AS1 regulates the cell cycle. Flow cytometric analysis showed that upregulation of SH3PXD2A-AS1 expression increased the percentage of cells in the S/G2 phases, and downregulation of SH3PXD2A-AS1 expression decreased the percentage of cells in the S/G2 phases (Fig. 2F, G).

### Identification of cytokine-related genes as probable target genes of SH3PXD2A-AS1 in NSCLC proliferation

**Fig. 3:**
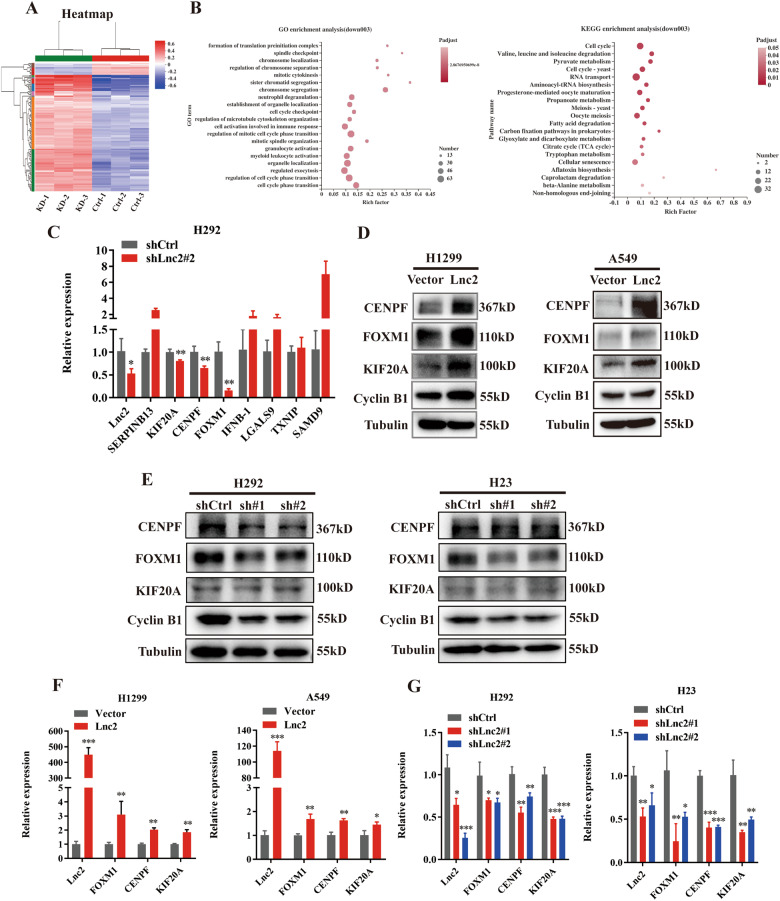
Identification of cytokine-related genes as probable target genes of SH3PXD2A-AS1 in NSCLC proliferation. **A** Cluster analysis of expression patterns of genes/transcripts in the selected gene set. The colour in the figure represents the normalised expression value of the gene in each sample. Red represents higher expression of the gene in the sample, and blue represents lower expression. **B** GO and KEGG enrichment analyses were used to classify the significantly differentially expressed genes of cancer cells. **C** The significantly differentially expressed genes of H292 cells with SH3PXD2A-AS1 knockdown in sequencing results were verified by qRT-PCR assays. **D, E** Effect of SH3PXD2A-AS1 OE or KD on the protein expression of FOXM1, KIF20A and CENPF in A549, H1299, H292 and H23 cells, as assessed by Western blottings. Tubulin was used as a reference control. **F, G** Relative mRNA expression levels of SH3PXD2A-AS1, FOXM1, KIF20A and CENPF in A549, H1299, H292 and H23 cells with SH3PXD2A-AS1 OE or KD. The 18 S gene was used as a reference control. Lnc2(SH3PXD2A-AS1). Data are shown as the mean ± standard deviation from three independent experiments. *P < 0.05, **P < 0.01, ***P < 0.001.

To further elucidate the mechanism by which SH3PXD2A-AS1 regulates lung cancer proliferation, we conducted gene expression profiling of SH3PXD2A-AS1 knockdown and vector control H292 cells in triplicate (Fig. 3A and S1A,B). GO and KEGG enrichment analysis revealed that many biological functions in 894 downregulated genes related to the cell cycle (Fig. 3B). Additionally, we verified the RNA-seq results by qRT-PCR and showed that the mRNA expression levels of CENPF, KIF20A and FOXM1 were decreased among the 8 top differentially expressed genes induced by SH3PXD2A-AS1 knockdown (Fig. 3C). We used pearson correlation analysis and identified a strong correlation between FOXM1 and CENPF (*P* < 0.001, R = 0.72) and FOXM1 and KIF20A (*P* < 0.001, R = 0.73) (Fig. S2A, B). The FOXM1 transcription factor is recognised as a regulator of cell cycle progression; thus, we also examined Cyclin B1. Western blotting was used to test the protein expression levels of FOXM1, Cyclin B1, KIF20A and CENPF, and the results showed that the protein expression was upregulated by SH3PXD2A-AS1 overexpression and inhibited by SH3PXD2A-AS1 knockdown (Fig. 3D, E). Then, qRT-PCR was used to test the mRNA expression levels of FOXM1, Cyclin B1, KIF20A and CENPF, and the results showed that these genes were upregulated by SH3PXD2A-AS1 overexpression and inhibited by SH3PXD2A-AS1 knockdown (Fig. 3F, G).

### SH3PXD2A-AS1 interacts with the DHX9 protein

**Fig. 4:**
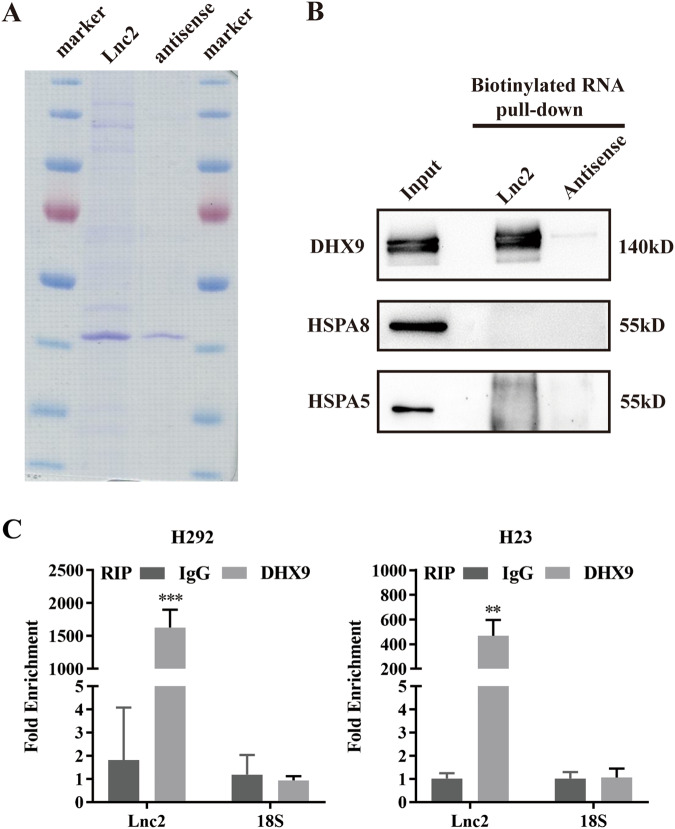
SH3PXD2A-AS1 interacts with the DHX9 protein. **A** Coomassie blue staining of biotinylated SH3PXD2A-AS1-associated proteins is shown. **B** RNA pulldown assays were performed to verify the mass spectrometry results. Biotin-SH3PXD2A-AS1 and antisense RNA were obtained by in vitro transcription by using T7 RNA polymerase. Western blotting was performed to determine DHX9, HSPA8 and HSPA5. **C** RIP assays were performed to test the interaction of SH3PXD2A-AS1 and DHX9. Relative quantification of SH3PXD2A-AS1 and 18 S rRNA in RNA-protein complexes immunoprecipitated with IgG or DHX9 from whole cell extracts; 18 S rRNA was used as a negative control binding RNA. Lnc2(SH3PXD2A-AS1). Data are shown as the mean ± standard deviation from three independent experiments. ***P < 0.001.

LncRNAs have been reported to exert biological functions by interacting with proteins. Therefore, to identify the SH3PXD2A-AS1-binding proteins, we performed in vitro transcription assays and RNA pulldown assays by using biotin-labelled SH3PXD2A-AS1 and a negative control in H292 cells (Fig. 4A). We performed Western blot analysis on RNA pulldown material to verify the MS results. The results indicated that SH3PXD2A-AS1 was associated with DHX9 but not HSPA8 or HSPA5 (Fig. 4B). Moreover, RIP assays were performed to confirm the significant interaction of SH3PXD2A-AS1 with DHX9 in H292 and H23 cells (Figs. 4C and S6A).

### Knockdown of DHX9 inhibits cell proliferation and cell cycle progression

**Fig. 5:**
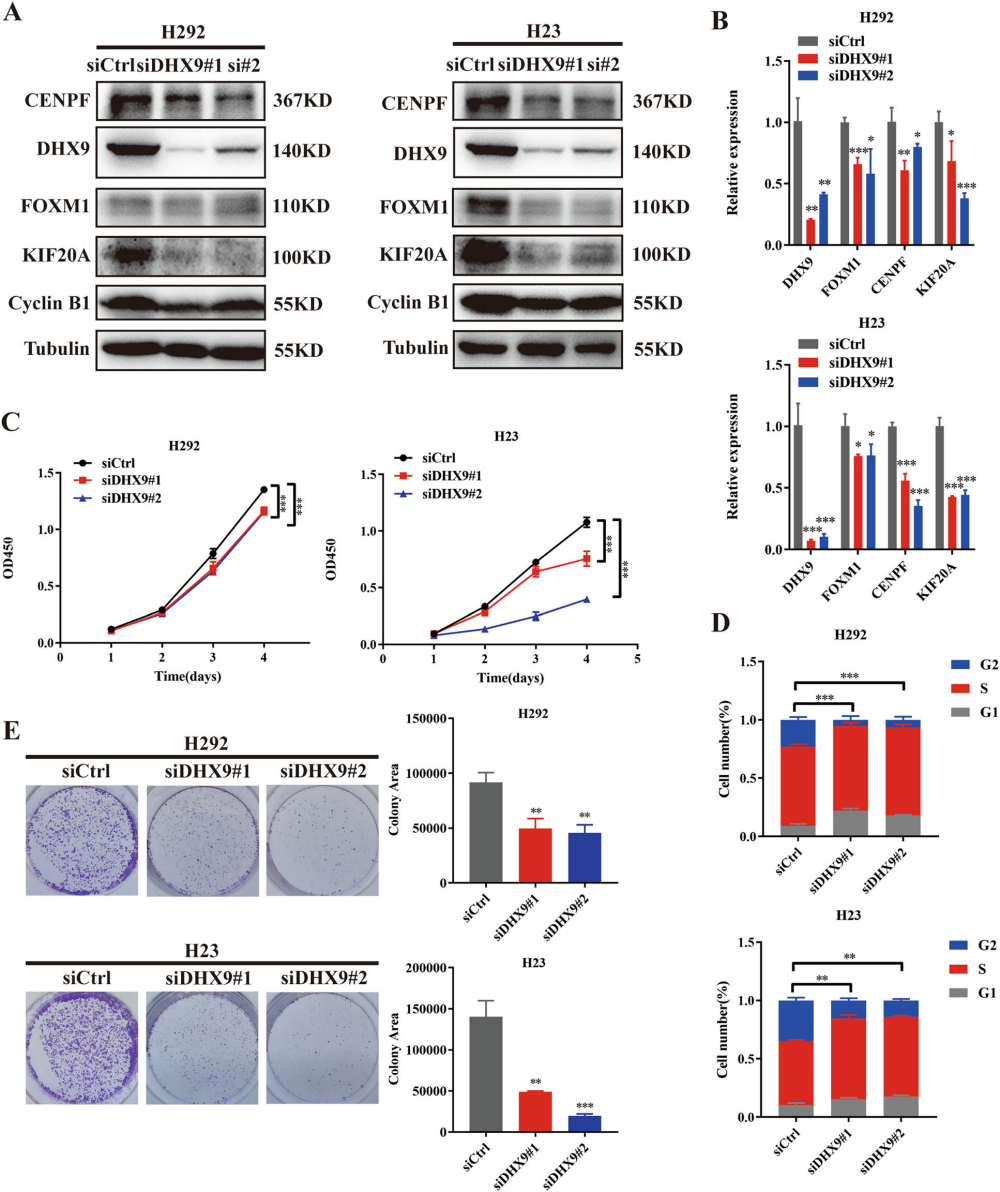
Knockdown of DHX9 inhibits cell proliferation and cell cycle progression. **A** Effect of DHX9 KD on the protein expression of DHX9, FOXM1, Cyclin B1, KIF20A and CENPF in H292 and H23 cells, as assessed by Western blotting assays. **B** Effect of DHX9 KD on the mRNA expression of DHX9, FOXM1, KIF20A and CENPF in H292 and H23 cells, as assessed by qRT-PCR assays. **C** Effect of DHX9 KD on H292 and H23 cell proliferation as assessed by Cell Counting Kit-8 assays. **D** The percentage of S/G2 population cells was measured by flow cytometry of DHX9 KD H292 and H23 cell lines. **E** Colony formation assays of DHX9 KD H292 and H23 cell lines. Data are shown as the mean ± standard deviation from three independent experiments. *P < 0.05, **P < 0.01, ***P < 0.001.

To determine the role of DHX9 in SH3PXD2A-AS1-mediated cell proliferation, we first employed western blotting and PCR to determine the effects of DHX9. The results indicated that knockdown of DHX9 by siRNAs inhibited the expression of FOXM1, Cyclin B1, KIF20A and CENPF at both the protein and mRNA levels in H292 and H23 cells (Fig. 5A, B). Then, we observed that the H292 and H23 cells with DHX9 knockdown showed significantly inhibited cell proliferation compared with the control cells (Fig. 5C, E). Furthermore, we found that the H292 and H23 cells with DHX9 knockdown had decreased percentages of cells in the S/G2 phases (Fig. 5D).

### DHX9 knockdown reverses the effects on proliferation and cell cycle progression induced by SH3PXD2A-AS1 overexpression

**Fig. 6:**
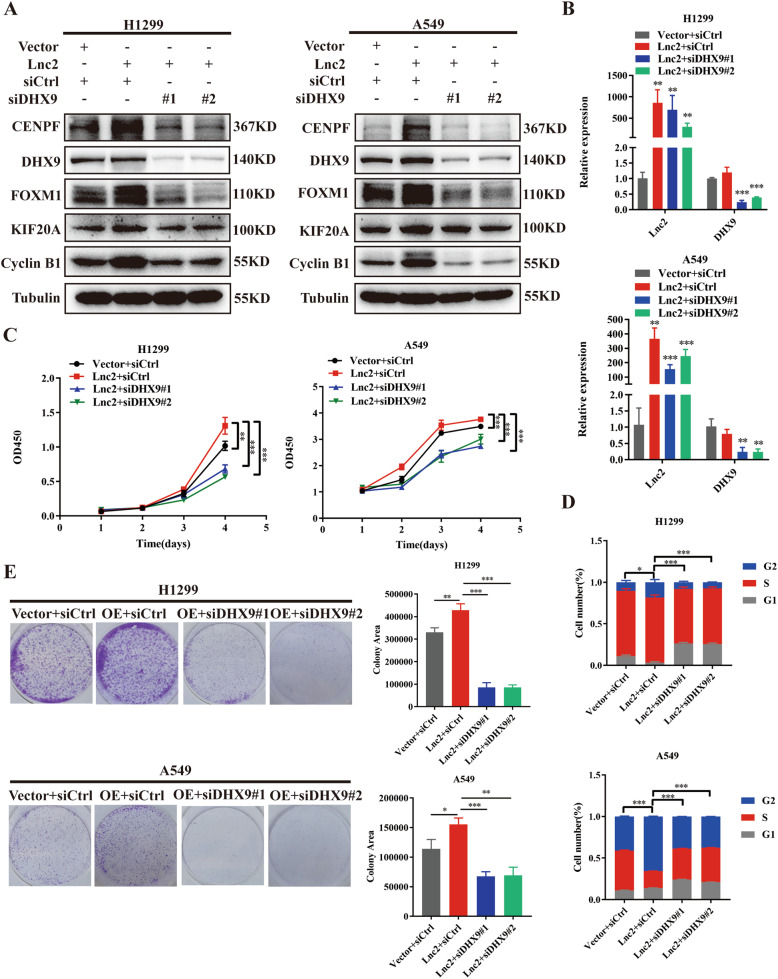
Downregulation of DHX9 reverses the effects on proliferation and cell cycle progression induced by SH3PXD2A-AS1 overexpression. **A** Western blotting assays were performed to measure the DHX9, FOXM1, Cyclin B1, KIF20A and CENPF protein levels after transfection of DHX9 siRNA in SH3PXD2A-AS1 overexpressing NSCLC cells. **B** qRT-PCR assays were performed to measure the SH3PXD2A-AS1, DHX9, FOXM1, Cyclin B1, KIF20A and CENPF mRNA levels after transfection of DHX9 siRNA in SH3PXD2A-AS1 overexpressing NSCLC cells. **C** Cell Counting Kit-8 assays for the effects of DHX9 KD on the proliferation of A549 and H1299 cells with SH3PXD2A-AS1 OE. **D** Flow cytometry for the effects of DHX9 KD on the cell cycle of A549 and H1299 cells with SH3PXD2A-AS1 OE. **E** Colony formation assays for the effects of DHX9 KD on the proliferation of A549 and H1299 cells with SH3PXD2A-AS1 OE. Lnc2(SH3PXD2A-AS1). Data are shown as the mean ± standard deviation from three independent experiments. *P < 0.05, **P < 0.01, ***P < 0.001.

To assess the role of DHX9 in proliferation and cell cycle progression induced by SH3PXD2A-AS1 overexpression, we silenced DHX9 by siRNA in H1299 and A549 cells with SH3PXD2A-AS1 overexpression (Fig. 6B). First, the Western blotting results showed that the FOXM1, Cyclin B1, KIF20A and CENPF levels, which were upregulated by SH3PXD2A-AS1 overexpression, were subsequently recovered following DHX9 knockdown (Fig. 6A). Moreover, the results showed that the increases in proliferation and the percentage of cells in the S/G2 phases caused by SH3PXD2A-AS1 overexpression were reversed by transient transfection of DHX9 siRNA in H1299 and A549 cells (Fig. 6C–E). These results demonstrated that DHX9 plays a crucial role in SH3PXD2A-AS1-regulated cell proliferation and cell cycle progression.

### Knockdown of SH3PXD2A-AS1 inhibits NSCLC cell proliferation in vivo

**Fig. 7:**
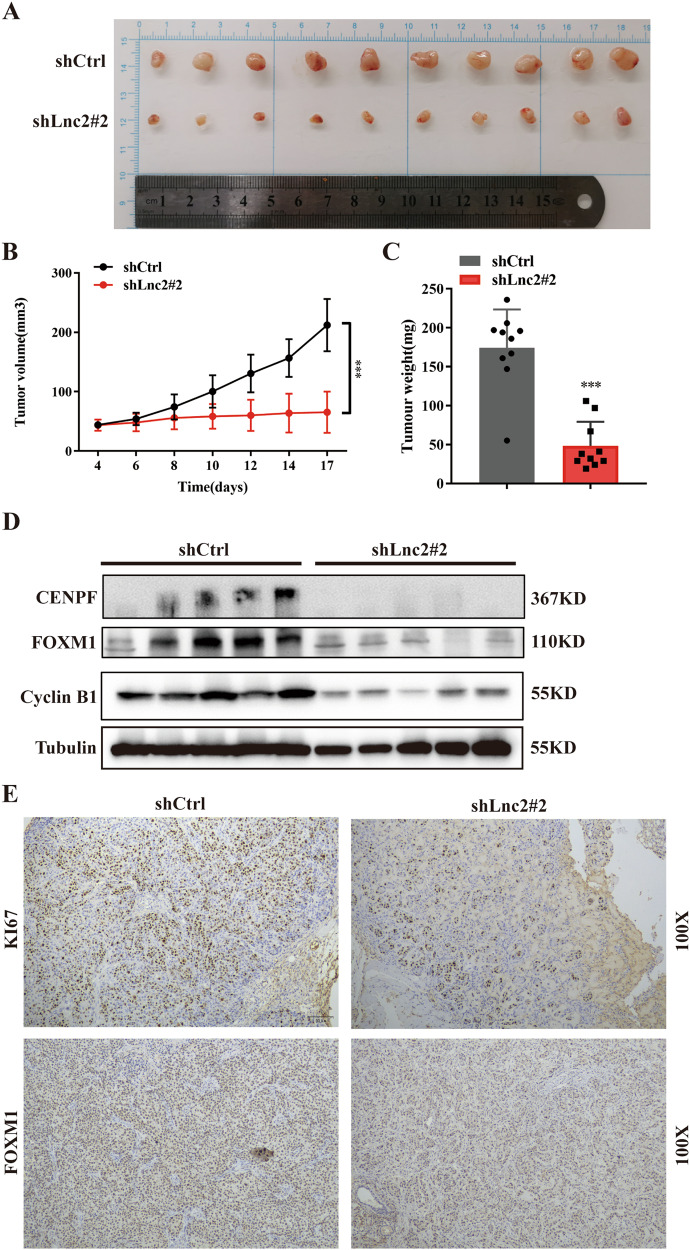
Knockdown of SH3PXD2A-AS1 inhibits NSCLC cell proliferation in vivo. **A**–**C** The effects of SH3PXD2A-AS1 KD on tumour volume and weight were assessed by using the xenograft model. A total of 5 × 10^6^ H292 SH3PXD2A-AS1 Con/KD cells and Matrigel (Corning; 1:1 ratio) were subcutaneously injected into the flanks of each mouse. ***P < 0.001. **D** Western blotting analysis of FOXM1, Cyclin B1 and CENPF expression in the tumour xenografts. Tubulin was used as a loading control. **E** The tumour sections were subjected to immunochemistry staining with antibodies against Ki67 and FOXM1, and representative images are shown. Lnc2(SH3PXD2A-AS1). ***P < 0.001.

To further confirm the role of SH3PXD2A-AS1 in vivo, we used a xenograft mouse model. H292 cells stably transfected with sh-SH3PXD2A-AS1 or an empty vector were subcutaneously injected into nude mice (control on left, sh-SH3PXD2A-AS1 on right). The results showed that the tumours derived from the SH3PXD2A-AS1 stable knockdown cells exhibited significantly smaller tumour volumes (Fig. 7A) and a lower tumour growth capacity (Fig. 7B) than the tumours from the control group. Seventeen days after injection, the average tumour weight in the sh-SH3PXD2A-AS1 group was significantly lower than that in the control group (Fig. 7C). Then Western blotting was used to test the protein expression levels of FOXM1, Cyclin B1 and CENPF in tumour tissues, the results showed that the protein expression were inhibited by SH3PXD2A-AS1 knockdown (Fig. 7D). Furthermore, IHC staining of tissue sections showed that the tumour tissues from the SH3PXD2A-AS1 knockdown group had a weaker staining intensity of Ki67 and FOXM1 than those from the control group (Fig. 7E). Taken together, these results suggest that knockdown of SH3PXD2A-AS1 inhibits the proliferation of NSCLC cells in vivo.

## Discussion

Lung cancer is the leading cause of cancer-related death. Emerging studies have shown that lncRNAs play a critical role in cancer. Although many lncRNAs have been identified in the human genome, very few have been experimentally validated and functionally annotated in lung cancer [21–23]. In the present study, we investigated a lncRNA (SH3PXD2A-AS1) that is markedly upregulated in lung cancer tissues. SH3PXD2A-AS1 is an antisense transcript transcribed from SH3PXD2A. The SH3PXD2A gene was expressed at higher levels in lung, colon, breast and prostate cancer tissues than in normal tissues [24]. Due to the dysregulation of SH3PXD2A gene expression has been shown in many diverse cancers [25–28], lncRNA generation in the SH3PXD2A genes may play important roles in tumorigenesis. SH3PXD2A-AS1 is significantly up-regulated in colorectal cancer tissues and cell lines, and promotes tumorigenesis through cell proliferation, migration and invasion [14, 15]. Our study showed that increased SH3PXD2A-AS1 expression was associated with a poor prognosis and shorter survival time in NSCLC patients. Our gene expression and functional data support the potential utility of SH3PXD2A-AS1 as a biomarker and as a therapeutic target for NSCLC.

The tumorigenesis and progression of NSCLC is accompanied or driven by infinite proliferation [29, 30]. Herein our data showed that SH3PXD2A-AS1 was highly expressed in NSCLC and was closely related to lung cancer cell proliferation and cell cycle progression. There is evidence that LncRNA can affect tumorigenesis by promoting the cell cycle process [31]. In summary, our results indicate that SH3PXD2A-AS1 may promote the proliferation of lung cancer cells by regulating the cell cycle process, thereby playing an important regulator role in NSCLC.

With regard to the potential of SH3PXD2A-AS1 in lung cancer proliferation, we further investigated the molecular mechanism of its functional properties. In this study, gene expression profiling analysis was used to reveal that hundreds of genes were regulated by SH3PXD2A-AS1 knockdown. We performed GO and KEGG enrichment analysis of 894 downregulated genes, and the results showed many biological functions such as regulation of cell cycle phase transition related to the cell cycle. Subsequently, we revealed that knockdown of SH3PXD2A-AS1 suppressed the expression of FOXM1, CENPF and KIF20A. FOXM1 is a critical proliferation-associated transcription factor that is widely spatiotemporally expressed during the cell cycle [32, 33]. FOXM1 is essential for progression to the DNA replication and mitosis stages and stimulates the proliferation of tumour cells during the progression of NSCLC via the Cyclin B1 [34]. CENPF is a cell cycle-associated nuclear antigen, and its mRNA controls the cell cycle by binding to lncRNA SMILR [35]. FOXM1 and CENPF expression is positively correlated with lncRNA SBF2-AS1 expression, and their synergistic interaction promotes the proliferation of lung cancer cells [36]. In addition, KIF20A regulated by lncRNA UCA1, may promote cell proliferation during the growth of cervical cancer cells [37]. As a FOXM1 target gene, KIF20A increases the proliferation and invasion of ovarian cancer cells [38]. FOXM1 has an effect on the biological functions of various cancers through synergistic interactions or transcriptional regulation with CENPF [39–41] and KIF20A [38, 42, 43]. Because FOXM1 has a strong correlation with CENPF and KIF20A, and there is a synergistic effect in the literature. Therefore, we use FOXM1 as the main downstream factor in this article. In our study, we found that there is a strong correlation between FOXM1 and CENPF and between FOXM1 and KIF20A. FOXM1, Cyclin B1, KIF20A and CENPF were inhibited by SH3PXD2A-AS1 knockdown. These findings indicated that FOXM1, CENPF and KIF20A partially contribute to SH3PXD2A-AS1-regulated lung cancer cell proliferation.

Given that protein changes resulted from SH3PXD2A-AS1 knockdown, we then elucidated the signalling pathways by which SH3PXD2A-AS1 regulated these proteins. Generally, lncRNAs exert their function by interacting with various RNA binding proteins, leading to inactivation or activation of gene expression. The lncRNA LINRIS interacts with IGF2BP2 to maintain the stability of IGF2BP2, and promote the progression of colorectal cancer [44]. Therefore, we used biotin-labelled SH3PXD2A-AS1 in RNA pulldown assays to identify interacting partners by mass spectrometry. The results indicated that DHX9 might be the most important transcriptional regulator. DHX9 is a member of the DExD/H-box family of helicases with the ability to unwind DNA and RNA duplexes [45]. In cervical cancer cells, the proto-oncogene DHX9 binds to the lncRNA lnc-CCDST and MDM2 to regulate cell invasion and angiogenesis [46]. DHX9 post‐transcriptionally inhibits CDK6 expression by binding to the CDK6‐3′UTR. LNC-UCID enhances the expression of CDK6 by binding to DHX9 in hepatocellular carcinoma, and promotes G1/S transformation and the growth of hepatocellular carcinoma [47]. Based on these findings, we speculate that DHX9 may act as an RNA binding protein to promote cell cycle progression by binding to SH3PXD2A-AS1. To further confirm the interaction between DHX9 and SH3PXD2A-AS1, we silenced DHX9 in NSCLC cells overexpressing SH3PXD2A-AS1. Our data showed that silencing DHX9 suppresses cell proliferation, cell cycle progression and the changes in the expression of the downstream factor FOXM1 induced by overexpression of SH3PXD2A-AS1. Hence, we revealed that SH3PXD2A-AS1 interacts with DHX9 to enhance FOXM1 expression to promote cell proliferation and cell cycle progression. However, we have not yet identified the specific binding sequence of SH3PXD2A-AS1 and DHX9, nor the clear mechanism for SH3PXD2A-AS1 and DHX9 binding to regulate FOXM1. We will explore these research topics in future experiments.

Overall, SH3PXD2A-AS1 can promote the expression of target genes such as FOXM1, CENPF and KIF20A by binding to the DHX9 protein, thereby promoting the cell cycle progression and cell proliferation of NSCLC, and promoting the growth of lung cancer.

## Conclusion

Our study shows for the first time that aberrant expression of SH3PXD2A-AS1 contributes to the proliferation and cell cycle progression of NSCLC. SH3PXD2A-AS1 regulates the expression of FOXM1 by binding to DHX9 in lung cancer cells to promote cell proliferation and cell cycle progression. These findings identify SH3PXD2A-AS1 as a potential biomarker and target for prognosis and therapy of NSCLC.

## Methods

### Bioinformatics analysis

GEPIA [48] (http://gepia.cancer-pku.cn/detail.php) was used to analyse the expression of SH3PXD2A-AS1 in NSCLC and normal lung tissues and in lung adenocarcinoma (LUAD) and normal lung tissues in TCGA database. In addition, K-M analysis was performed to explore the effects of target genes on the survival rate in NSCLC and LUAD. Correlation analysis showed the correlation between the genes using the nonlog scale for calculation and the log-scale axis for visualisation.

### Clinical samples

LUAD tissues were obtained from the Affiliated Hospital of Xuzhou Medical University. All specimens were pathologically confirmed as NSCLC, and the patients had not received radiotherapy or chemotherapy prior to surgery at the Affiliated Hospital of Xuzhou Medical University. After resection, the tumour and adjacent tissues were frozen in liquid nitrogen, and the specimens were immediately stored at −80 °C. The patient studies were conducted in accordance with the Declaration of Helsinki. This study was conducted in compliance with the Declaration of Helsinki. The use of these specimens and data for research purposes was approved by the Ethics Committee of the Affiliated Hospital of Xuzhou Medical University.

### Cell lines and cell culture

The immortalised normal human lung epithelial cell line BEAS-2B and the human NSCLC cell lines A549, H1299, H292 and H23 were purchased from the Shanghai Institute of Biochemistry and Cell Biology, Chinese Academy of Science (Shanghai China). BEAS-2B, A549, H1299, H292 and H23 cells were cultured in DMEM medium and RPMI 1640 medium supplemented with 10% foetal bovine serum, 100 U/ml penicillin, and 100 μg/ml streptomycin and incubated in a 37 °C humidified incubator with 5% CO_2_.

### Transient transfections and stable cell lines

PCDNA3.1-SH3PXD2A-AS1, PCDNA3.1-vector, shRNA-SH3PXD2A-AS1, and shRNA-ctrl vectors were transfected into NSCLC cells by Lipofectamine 2000 transfection reagent (Invitrogen, Shanghai, China). SiRNA-DHX9 was transfected into NSCLC cells by SilenFect reagent (Thermo Fisher Scientific, Inc., USA), while nonspecific siRNA was used as a negative control. All siRNAs were purchased from GenePharma Technology (Shanghai, China). SH3PXD2A-AS1 shRNA sequences were cloned into the vector pLko.1 at Age1/ECOR1 sites. SH3PXD2A-AS1 knockdown lentiviruses were generated by cotransfecting 293 T cells with two packaging vectors, pMD2G and psPAX. The supernatants of cultured 293 T cells were collected 48 h later, filtered through 0.45-mm filters (Millipore, Temecula, CA, USA) and concentrated using Amico Ultra centrifugal filters (Millipore 100 KD MWCO). H292 cells were infected with lentivirus for 48 h and then selected with 2 ng/ml puromycin for 2 weeks, with the medium refreshed every 3 days. The sequences are listed in below:

shSH3PXD2A-AS1#1-For: CCGGGCAGCTCAGGTGTATGTAAGGCTCGAGCCTTACATACACCTGAGCTGCTTTTTG;

shSH3PXD2A-AS1#1-Rev: AATTCAAAAAGCAGCTCAGGTGTATGTAAGGCTCGAGCCTTACATACACCTGAGCTGC;

shSH3PXD2A-AS1#2-For: CCGGGCACCAAGAGAGCCCTAAAGACTCGAGTCTTTAGGGCTCTCTTGGTGCTTTTTG;

shSH3PXD2A-AS1#2-Rev: AATTCAAAAAGCACCAAGAGAGCCCTAAAGACTCGAGTCTTTAGGGCTCTCTTGGTGC.

siDHX9#1: GAGCCAACUUGAAGGAUUATTUAAUCCUUCAAGUUGGCUCTT

siDHX9#2: CCUGGGAUGAUGCUAGAAUTTAUUCUAGCAUCAUCCCAGGTT.

### Cell proliferation and colony formation assays

Forty-eight hours after transfection, for CCK-8 analysis, ~4 × 10^3^ cells were seeded in each well of 96-well plates, and CCK-8 solution was added 24, 48, 72, and 96 h after placing. Cells were incubated at 37 °C for 2 h after 10 μl of CCK-8 solution was added. The absorbance at 450 nm was measured. For the colony formation assay, 1 × 10^3^ cells were cultured in six-well plates at 37 °C for 14 days; visible colonies were washed twice with PBS, fixed, and stained with 4% paraformaldehyde and crystal violet. The area of colony formation is measured by Image Pro Plus 6.0 and calculated with graphpad.

### Cell cycle analysis

Cells were treated with 1 μg/ml aphidicolin at 48 h after transfection. After 12 h, the cells were incubated in fresh medium containing 50 ng/ml nocodazole for 0, 3 or 6 h. Then, the cells were fixed with 70% cold ethanol at 4 °C overnight and stained with 40 μg/ml propidium iodide in hypotonic fluorochrome buffer for 30 min. The samples were then analysed using a FACSCanto flow cytometer (BD Biosciences, San Jose, CA).whoT.

### Differential expression analysis and functional enrichment

For identification of differentially expressed genes (DEG) between two different samples, the expression level of each transcript was calculated according to the fragments per kilobase of exon per million mapped reads (FRKM) method. RSEM (http://deweylab.biostat.wisc.edu/rsem/) [49] was used to quantify gene abundances. The R statistical package software EdgeR (Empirical analysis of Digital Gene Expression in R, (http://www.bioconductor.org/packages/2.12/bioc/html/edgeR.html)) [50] was utilised for differential expression analysis. In addition, functional enrichment analysis, including GO and KEGG analyses, was performed to identify which DEGs were significantly enriched in GO terms and metabolic pathways with a Bonferroni-corrected *P*-value ≤ 0.05 compared with the whole-transcriptome background. GO functional enrichment and KEGG pathway analysis were carried out by Goatools (https://github.com/tanghaibao/Goatools) and KOBAS (http://kobas.cbi.pku.edu.cn/home.do) [51].

### RNA extraction, reverse transcription-PCR and qRT-PCR

Total RNA from the lung tissue specimens and cell lines used in this study was extracted with TRIzol reagent (Vazyme Biotech, Nanjing, China). We synthesised cDNA by using HiScript Q RT SuperMix for qPCR (+gDNA wiper) (Vazyme Biotech, Nanjing, China). Relative RNA levels determined by RT-qPCR were measured on a Roche LightCycler 480 by using UltraSYBR Mixture (CWBIO, Beijing, China). The primers used for quantitative RT-PCR analysis are listed below:

SH3PXD2A-AS1-For: CAGGAGTGTGCCACCATGCTTG;

SH3PXD2A-AS1-Rev: GGCAAGACTGGCTCATGAACTCTC;

SERPINB3-For: AGATTAACTCCTGGGTGGAAAG;

SERPINB3-Rev: CAATGTGGTATTGCTGCCAATA;

KIF20A-For: GAATGTGGAGACCCTTGTTCTA;

KIF20A-Rev: CCATCTCCTTCACAGTTAGGTT;

CENPF-For: TACAACGAGAGAGTAAGAACGC;

CENPF-Rev: CTACCTCCACTGACTTACTGTC;

FOXM1-For: GATCTGCGAGATTTTGGTACAC;

FOXM1-Rev: CTGCAGAAGAAAGAGGAGCTAT;

IFNB-1-For: TGGCTGGAATGAGACTATTGTT;

IFNB-1-Rev: GGTAATGCAGAATCCTCCCATA;

LGALS3-For: CAGACAATTTTTCGCTCCATGA;

LGALS3-Rev: TAGGCCCCAGGATAGGAAG;

TXNIP-For: GTTCAGAAGATCAGGCCTTCTA;

TXNIP-Rev: TCCAGGAACGCTAACATAGATC;

SAMD9-For: GCTTGAAAGTATCCATCGGTTC;

SAMD9-Rev: TCAACTGAAATGTTCCCGTTTC;

DHX9-For: TCCAACTGGAATCCTTGGAC;

DHX9-Rev: TTTTCCCACATCCAGTAGCC;

18 S rRNA-For: GTAACCCGTTGAACCCCATT;

18 S rRNA-Rev: CCATCCAATCGGTAGTAGCG.

### Western blotting analysis

Western blotting was carried out as previously reported. Briefly, protein extracts from cells or immunoprecipitation samples were prepared using detergent-containing lysis buffer. Total protein was subjected to SDS-PAGE and transferred to 0.45 μm PVDF membranes (Millipore). Antibodies against FOXM1 (Proteintech, 13147-1-AP, 1:1000, USA), CENPF (Affinity, DF2310, 1:1000, China), KIF20A (Affinity, DF8671, 1:2000 China), Cyclin B1 (Cell Signaling Technology, 12231, 1:1000, USA), HSPA5 (Proteintech, 115871-AP, 1:2000, USA), HSPA8 (Proteintech, 10654-1-AP, 1:2000, USA), DHX9 (Proteintech, 17721-1-AP, 1:2000, USA), and alpha tubulin (Proteintech, 66031-1-Ig, 1:100000, USA) were used for primary antibody incubation at 4 °C overnight.

### RNA pulldown assay and mass spectrometry

RNA pulldown assays were carried out as described briefly: in vitro biotin-labelled RNAs (SH3PXD2A-AS1, its antisense RNA) were transcribed with Biotin RNA Labeling Mix (Promega Corporation, USA) and T7 RNA polymerase (Thermo Fisher Scientific, USA) treated with RNase inhibitor and purified with a Clean-up kit (Promega Corporation, USA). The biotinylated SH3PXD2A-AS1 probes were dissolved in binding and washing buffer and incubated with streptavidin agarose resin (Thermo Fisher Scientific, USA). Then, H292 cell lysates were incubated with probe-coated streptavidin beads, and the pulled-down proteins were run on SDS-PAGE gels. Then, the gels were stained with Coomassie Blue, and differentially abundant bands were cut out for mass spectrometry (Shanghai Applied Protein Technology Co., Ltd., China).

### RNA immunoprecipitation

The RIP experiment was carried out with the EZ-Magna RIP Kit (Millipore) according to the manufacturer’s protocol using 5 mg of antibody. Cells were lysed in complete RIP lysis buffer, and the cell extract was incubated with protein A/g agarose beads conjugated with antibody DHX9 (17721-1-AP, Proteintech, USA) or control IgG for 2 h at 4 °C. Beads were washed and incubated with Proteinase K to remove proteins. Finally, purified RNA was subjected to quantitative RT-PCR analysis.

### Animal experiments

Female BALB/cJGpt-Foxn1^nu^/Gpt mice (6-8 weeks old) were purchased from Nanjing GemPharmatech Technology Co., Ltd. (Nanjing, China). All animal experiments were approved by the Animal Care and Use Committee of Xuzhou Medical University. Groups of H292-shCtrl and H292-shSH3PXD2A-AS1 cells (5 × 10^6^) were injected subcutaneously into the flanks of mice. Tumour volume (V) was monitored every 2/3 days by measuring the long axis (L) and the short axis (W) of xenograft tumours and calculated with the following formula: V = (L×W^2^)/2. The animals were euthanized by cervical dislocation according to the American Veterinary Medical Association Guidelines (Schaumburg, IL, USA).

### Immunohistochemistry (IHC)

IHC assays were implemented following a standard streptavidin-peroxidase (SP) method as previously reported [52], and heat-induced epitope retrieval (HIER) was performed with retrieval buffer (citrate, pH 6.0) prior to commencing the IHC staining protocol. For primary antibody incubation, anti-FOXM1 (sc-376471, santa cruz, USA) antibody at a 1:100 dilution and anti-Ki67 (ab16667, Abcam, USA) antibodies at a 1:200 dilution were applied. The slide without primary antibody incubation served as a negative control.

### Statistical analysis

Statistical analyses were carried out using SPSS 20.0 software (SPSS, Inc., Chicago, IL, USA) and GraphPad Prism 8. The Kaplan–Meier method and log-rank test were used to evaluate the correlation between SH3PXD2A-AS1 expression and NSCLC/LUAD patient survival. The unpaired t test was used to determine the statistical significance of differences between groups. Data are presented as the mean ± SD. *P* < 0.05 was considered statistically significant.

### Role of funding source

The study design was approved by the Funder. The funders played no role in data collection, analysis, or interpretation of the data, or drafting of the manuscript. The corresponding author had full access to all the data in the study and had final responsibility for the decision to submit for publication.

## Supplementary information

Reproducibility checklist

Supplemental material

Table S1 Text of mass spectrometry result.

Table S2 Text of RNA-Seq result.

Table S3 Text of GO enrichment result.

Table S4 Text of KEGG enrichment result.

original western blots

## Data Availability

The datasets used and/or analysed during the current study are available from the corresponding author on reasonable request.
