# Correction: Long noncoding RNA SH3PXD2A-AS1 promotes NSCLC proliferation and accelerates cell cycle progression by interacting with DHX9

**DOI:** 10.1038/s41420-023-01596-7

**Published:** 2023-08-21

**Authors:** Yeqing Zhou, Hongmei Yong, WenJie Cui, Sufang Chu, Minle Li, Zhongwei Li, Jin Bai, Hao Zhang

**Affiliations:** 1 Thoracic Surgery Laboratory, Xuzhou Medical University, Xuzhou, 221006 Jiangsu Province China; 2 Department of Thoracic Surgery, Affiliated Hospital of Xuzhou Medical University, 99 West Huaihai Road, Xuzhou, 221006 Jiangsu China; 3 Department of Thoracic Surgery, Shengze Hospital in Jiangsu, Suzhou, 215228 Jiangsu China; 4 Department of Oncology, the Affiliated Huai’an Hospital of Xuzhou Medical University and The Second People’s Hospital of Huai’an, Huai’an, Jiangsu China; 5 Department of Respiratory and Critical Care Medicine, The Municipal Hospital Affiliated to Xuzhou Medical University, Xuzhou, Jiangsu China; 6 Cancer Institute, Xuzhou Medical University, Xuzhou, 221006 Jiangsu China; 7 Center of Clinical Oncology, the Affiliated Hospital of Xuzhou Medical University, Xuzhou, 221006 Jiangsu China

**Keywords:** Cancer, Cell growth

Correction to: *Cell Death Discovery* 10.1038/s41420-022-01004-6, published online 11 April 2022

In this article figure 5 has been corrected:
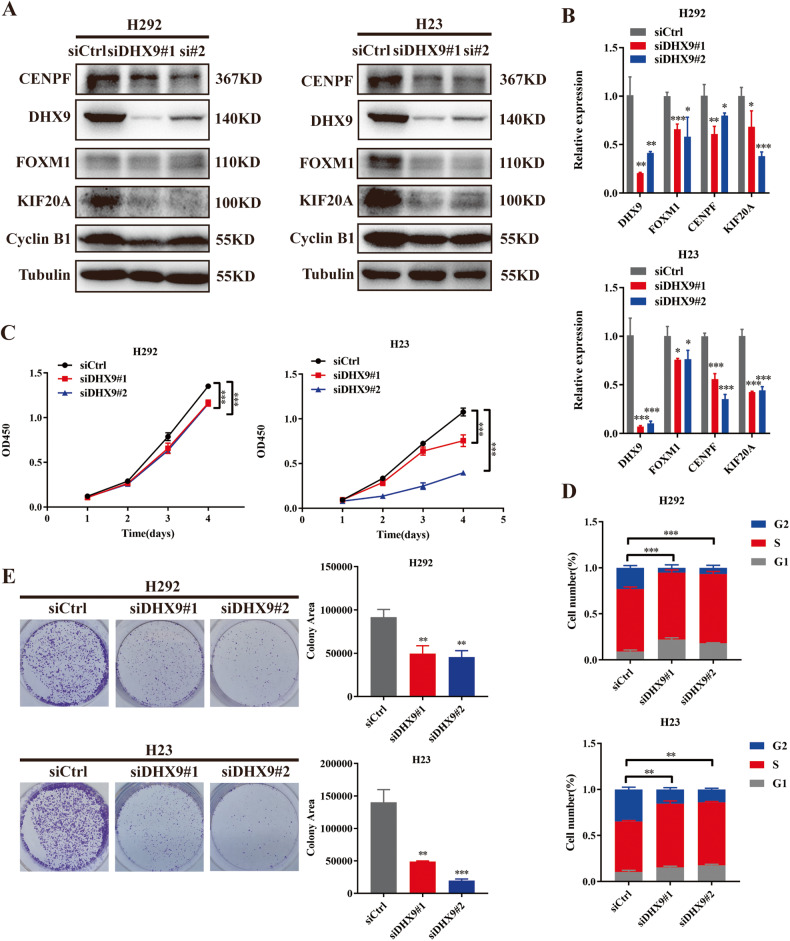


**Fig. 5 Knockdown of DHX9 inhibits cell proliferation and cell cycle progression**. **A** Effect of DHX9 KD on the protein expression of DHX9, FOXM1, Cyclin B1, KIF20A and CENPF in H292 and H23 cells, as assessed by Western blotting assays. **B** Effect of DHX9 KD on the mRNA expression of DHX9, FOXM1, KIF20A and CENPF in H292 and H23 cells, as assessed by qRT-PCR assays. **C** Effect of DHX9 KD on H292 and H23 cell proliferation as assessed by Cell Counting Kit-8 assays. **D** The percentage of S/G2 population cells was measured by flow cytometry of DHX9 KD H292 and H23 cell lines. **E** Colony formation assays of DHX9 KD H292 and H23 cell lines. Data are shown as the mean ± standard deviation from three independent experiments. *P < 0.05, **P < 0.01, ***P < 0.001. The original article has been corrected.

