# Supplementary material for: Long noncoding RNA SH3PXD2A-AS1 promotes NSCLC proliferation and accelerates cell cycle progression by interacting with DHX9
Source: Cell Death Discov. 2022 Apr 11;8:192. doi: 10.1038/s41420-022-01004-6 (PMC9001675; doi:10.1038/s41420-022-01004-6)
Supplement: Supplementary file 2 — Supplemental material [file 41420_2022_1004_MOESM2_ESM.pdf]

Supplemental material

Supplemental Figures and Figure Legends

Fig. S1 Identification of cytokine-related genes as probable target genes of SH3PXD2A-AS1. A Principal component analysis (PCA) shows the relationship and variation between samples. **B** The volcano map shows the gene/transcript expression differences between samples. The abscissa is the multiple of the difference in gene/transcript expression between the two samples, and the ordinate is the statistical test value of the difference in gene expression.

Fig. S2 Correlation analysis showed the correlations among FOXM1, CENPF, KIF20A and SH3PXD2A-AS1. A, B The correlations among FOXM1, CENPF and KIF20A in LUAD tissue as assessed by GEPIA. **C, D, E** The correlations between SH3PXD2A-AS1 and FOXM1, SH3PXD2A-AS1 and CENPF, SH3PXD2A-AS1 and KIF20A in LUAD tissue as assessed by GEPIA. We used the nonlog scale for calculation and used the log-scale axis for visualization. Statistical analysis: Data were analysed using Pearson correlation analysis.

Fig. S3 SH3PXD2A-AS1 accelerates lung cancer cell cycle progression. A, B The original FACS images for cell cycle analysis.

Fig. S4 Downregulation of DHX9 reverses the effects on cell cycle progression induced by SH3PXD2A-AS1 overexpression. A, B The original FACS images for cell cycle analysis.

Fig. S5 Knockdown of SH3PXD2A-AS1 inhibits cell metastasis. A, B Effects of SH3PXD2A-AS1 knockdown on migration and invasion of H292 and H23 cells. Lnc2(SH3PXD2A-AS1). ** $P < 0.01$, *** $P < 0.001$.

Fig. S6 Overexpression of SH3PXD2A-AS1 and DHX9 in LUAD correlates with poor prognosis. A Kaplan–Meier survival curve analysis was performed to explore the

effects of SH3PXD2A-AS1 and DHX9 on the overall survival rate (*P*<0.01) or disease free survival (*P*=0.05) in LUAD.

Fig. S7 Quantitative statistics of Western blottings. A Western blotting analysis for SH3PXD2A-AS1 OE in H1299 and A549 cells. **B** Western blotting analysis for SH3PXD2A-AS1 KD in H292 and H23 cells. **C** Western blotting analysis for DHX9 KD in H292 and H23 cells. **D** Western blotting analysis for SH3PXD2A-AS1 OE+DHX9 KD in H1299 and A549 cells. Lnc2(SH3PXD2A-AS1). Data are shown as the mean ± standard deviation from three independent experiments. *$P < 0.05$, **$P < 0.01$, ***$P < 0.001$.

Table S1 Text of mass spectrometry result.

Table S2 Text of RNA-Seq result.

Table S3 Text of GO enrichment result.

Table S4 Text of KEGG enrichment result.

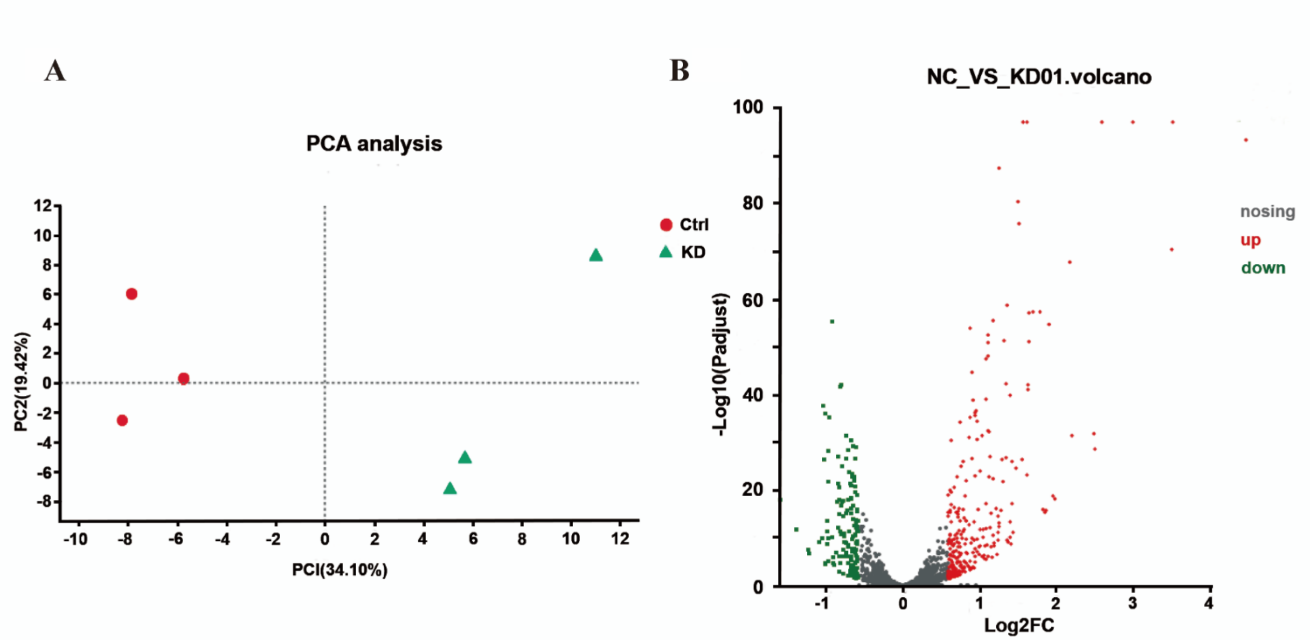
A
PCA analysis
PC2(19.42%)
PCI(34.10%)
Ctrl
KD
B
NC_VS_KD01.volcano
-Log10(Padjust)
Log2FC
nosing
up
down

Figure S1

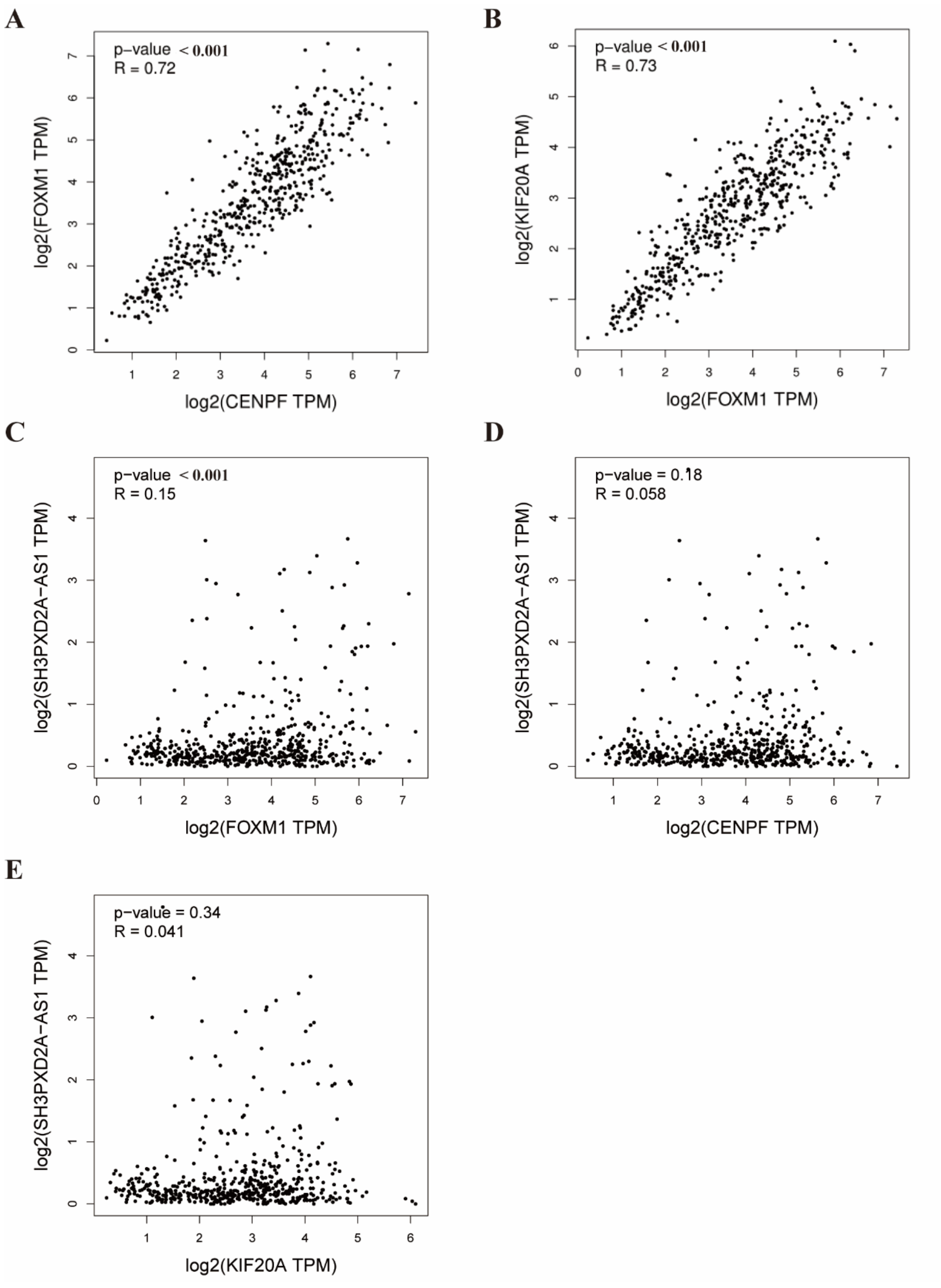
A
p−value < 0.001
R = 0.72
log2(FOXM1 TPM)
log2(CENPF TPM)
B
p−value < 0.001
R = 0.73
log2(KIF20A TPM)
log2(FOXM1 TPM)
C
p−value < 0.001
R = 0.15
log2(SH3PXD2A−AS1 TPM)
log2(FOXM1 TPM)
D
p−value = 0.18
R = 0.058
log2(SH3PXD2A−AS1 TPM)
log2(CENPF TPM)
E
p−value = 0.34
R = 0.041
log2(SH3PXD2A−AS1 TPM)
log2(KIF20A TPM)

Figure S2

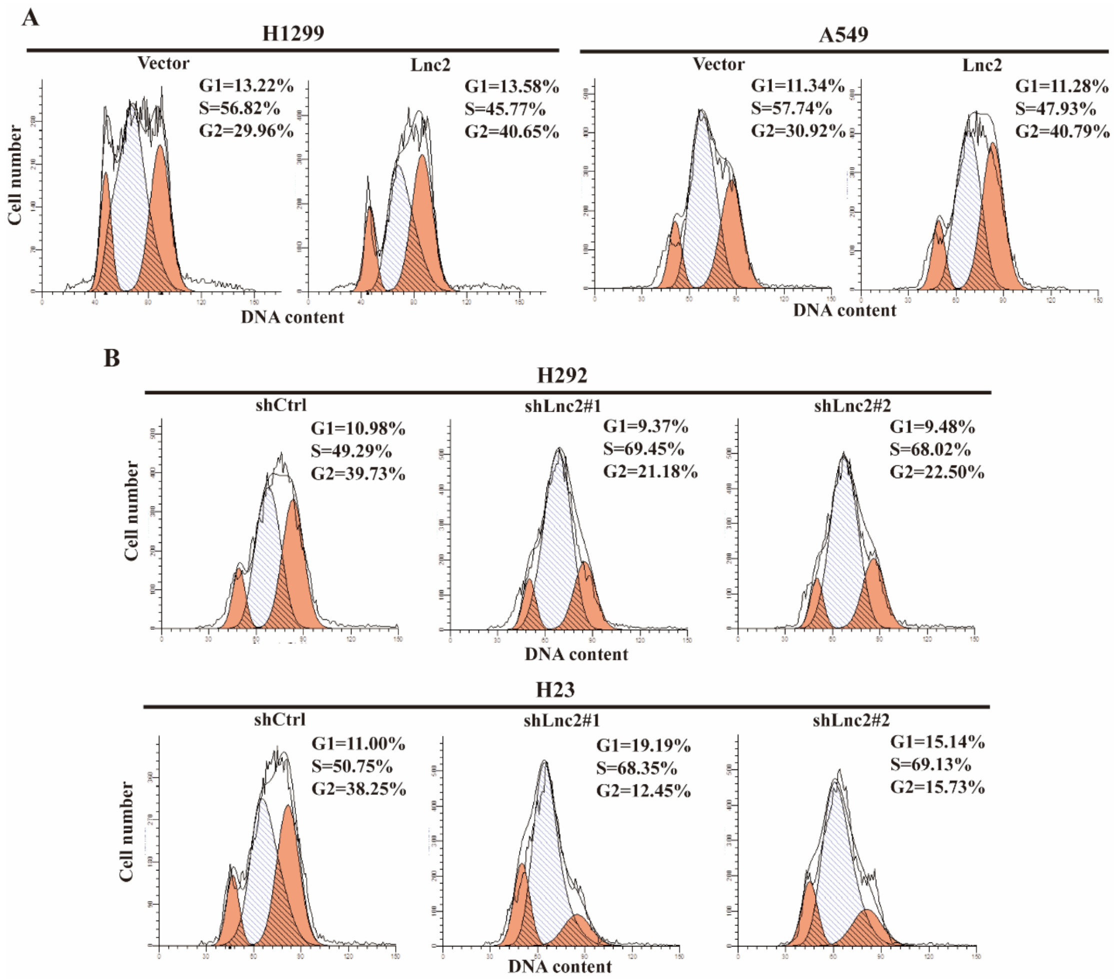
A
H1299
A549
Vector
Lnc2
Vector
Lnc2
G1=13.22%
S=56.82%
G2=29.96%
G1=13.58%
S=45.77%
G2=40.65%
G1=11.34%
S=57.74%
G2=30.92%
G1=11.28%
S=47.93%
G2=40.79%
Cell number
DNA content
DNA content
B
H292
shCtrl
shLnc2#1
shLnc2#2
G1=10.98%
S=49.29%
G2=39.73%
G1=9.37%
S=69.45%
G2=21.18%
G1=9.48%
S=68.02%
G2=22.50%
Cell number
DNA content
H23
shCtrl
shLnc2#1
shLnc2#2
G1=11.00%
S=50.75%
G2=38.25%
G1=19.19%
S=68.35%
G2=12.45%
G1=15.14%
S=69.13%
G2=15.73%
Cell number
DNA content

Figure S3

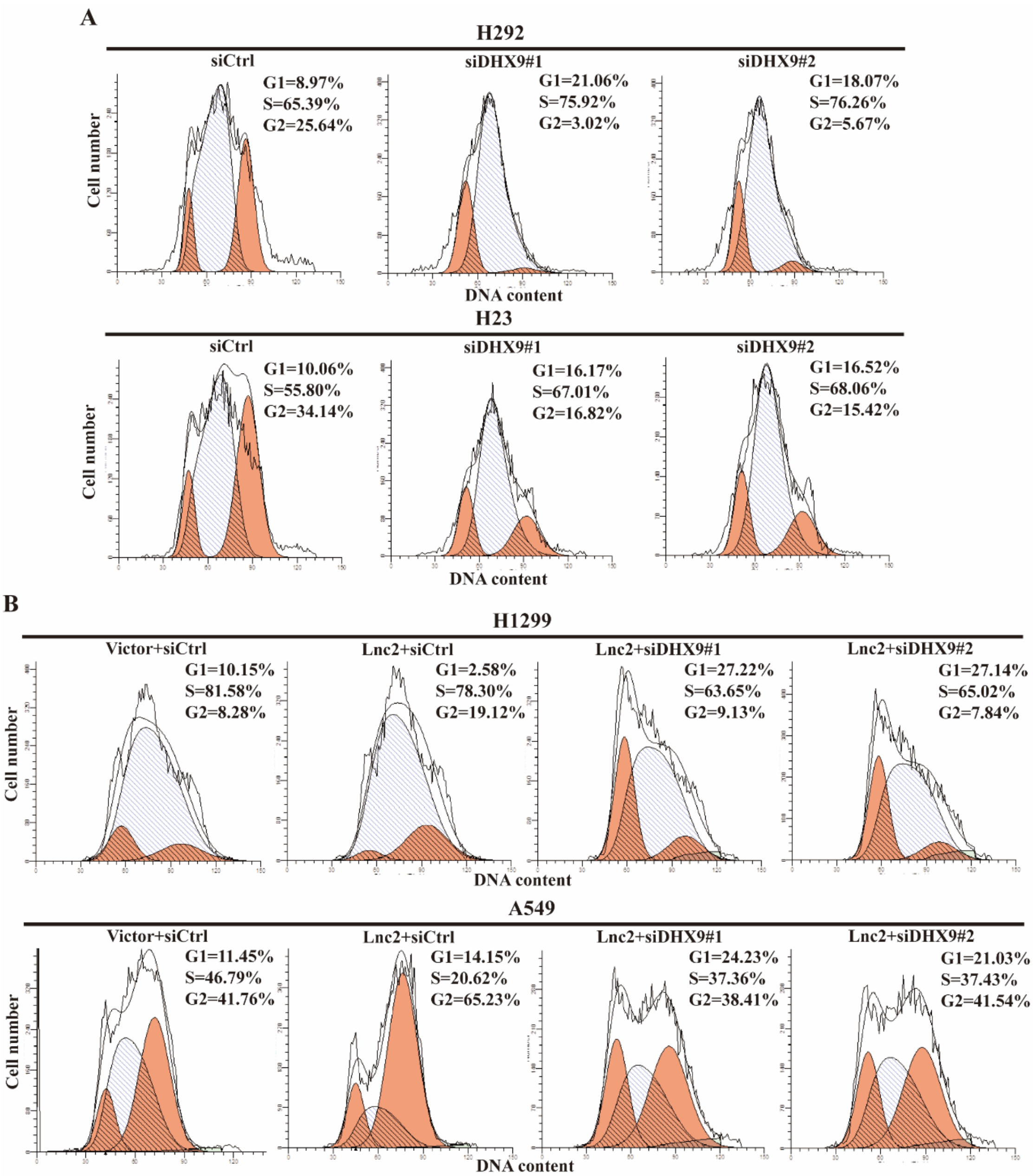
A
H292
siCtrl
G1=8.97%
S=65.39%
G2=25.64%
siDHX9#1
G1=21.06%
S=75.92%
G2=3.02%
siDHX9#2
G1=18.07%
S=76.26%
G2=5.67%
Cell number
DNA content
H23
siCtrl
G1=10.06%
S=55.80%
G2=34.14%
siDHX9#1
G1=16.17%
S=67.01%
G2=16.82%
siDHX9#2
G1=16.52%
S=68.06%
G2=15.42%
Cell number
DNA content
B
H1299
Victor+siCtrl
G1=10.15%
S=81.58%
G2=8.28%
Lnc2+siCtrl
G1=2.58%
S=78.30%
G2=19.12%
Lnc2+siDHX9#1
G1=27.22%
S=63.65%
G2=9.13%
Lnc2+siDHX9#2
G1=27.14%
S=65.02%
G2=7.84%
Cell number
DNA content
A549
Victor+siCtrl
G1=11.45%
S=46.79%
G2=41.76%
Lnc2+siCtrl
G1=14.15%
S=20.62%
G2=65.23%
Lnc2+siDHX9#1
G1=24.23%
S=37.36%
G2=38.41%
Lnc2+siDHX9#2
G1=21.03%
S=37.43%
G2=41.54%
Cell number
DNA content

Figure S4

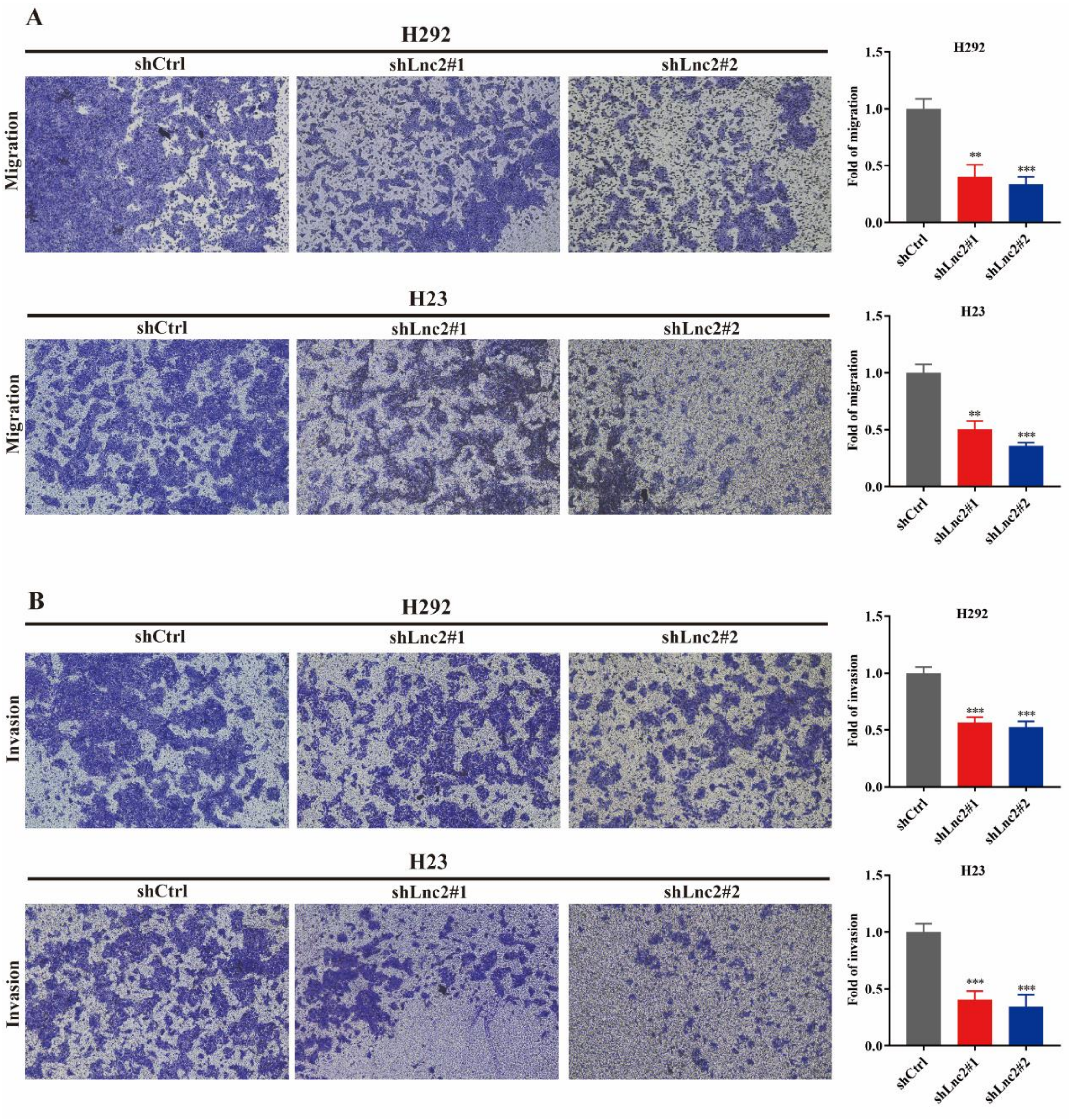
A
H292
shCtrl
shLnc2#1
shLnc2#2
Migration
H292
Fold of migration
1.5
1.0
0.5
0.0
**
***
shCtrl
shLnc2#1
shLnc2#2
H23
shCtrl
shLnc2#1
shLnc2#2
Migration
H23
Fold of migration
1.5
1.0
0.5
0.0
**
***
shCtrl
shLnc2#1
shLnc2#2
B
H292
shCtrl
shLnc2#1
shLnc2#2
Invasion
H292
Fold of invasion
1.5
1.0
0.5
0.0
***
***
shCtrl
shLnc2#1
shLnc2#2
H23
shCtrl
shLnc2#1
shLnc2#2
Invasion
H23
Fold of invasion
1.5
1.0
0.5
0.0
***
***
shCtrl
shLnc2#1
shLnc2#2

Figure S5

A

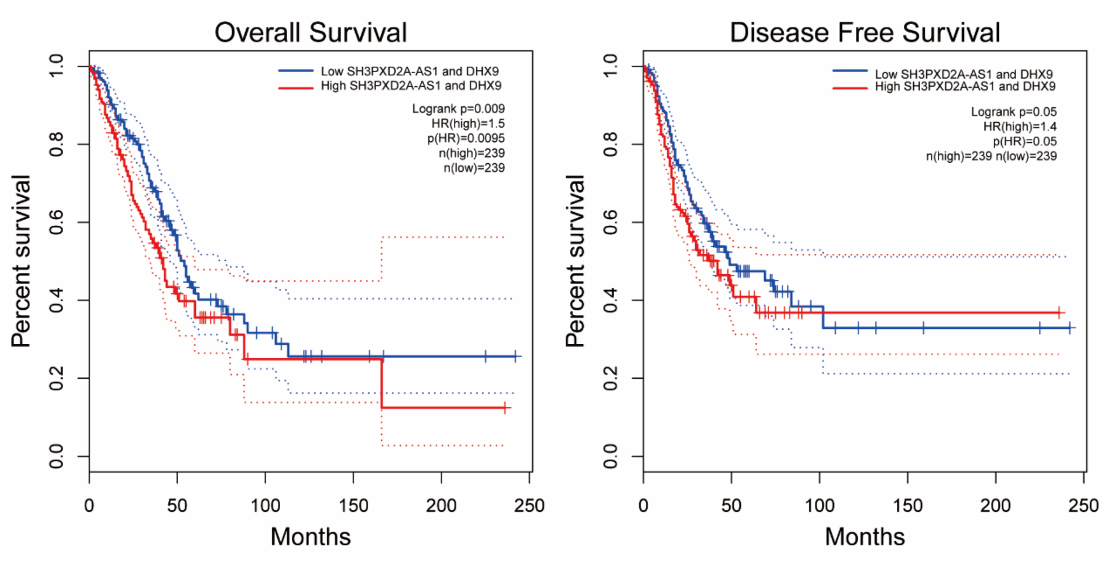

Overall Survival
Low SH3PXD2A-AS1 and DHX9
High SH3PXD2A-AS1 and DHX9
Logrank p=0.009
HR(high)=1.5
p(HR)=0.0095
n(high)=239
n(low)=239
Percent survival
0.0
0.2
0.4
0.6
0.8
1.0
0
50
100
150
200
250
Months
Disease Free Survival
Low SH3PXD2A-AS1 and DHX9
High SH3PXD2A-AS1 and DHX9
Logrank p=0.05
HR(high)=1.4
p(HR)=0.05
n(high)=239 n(low)=239
Percent survival
0.0
0.2
0.4
0.6
0.8
1.0
0
50
100
150
200
250
Months

Figure S6

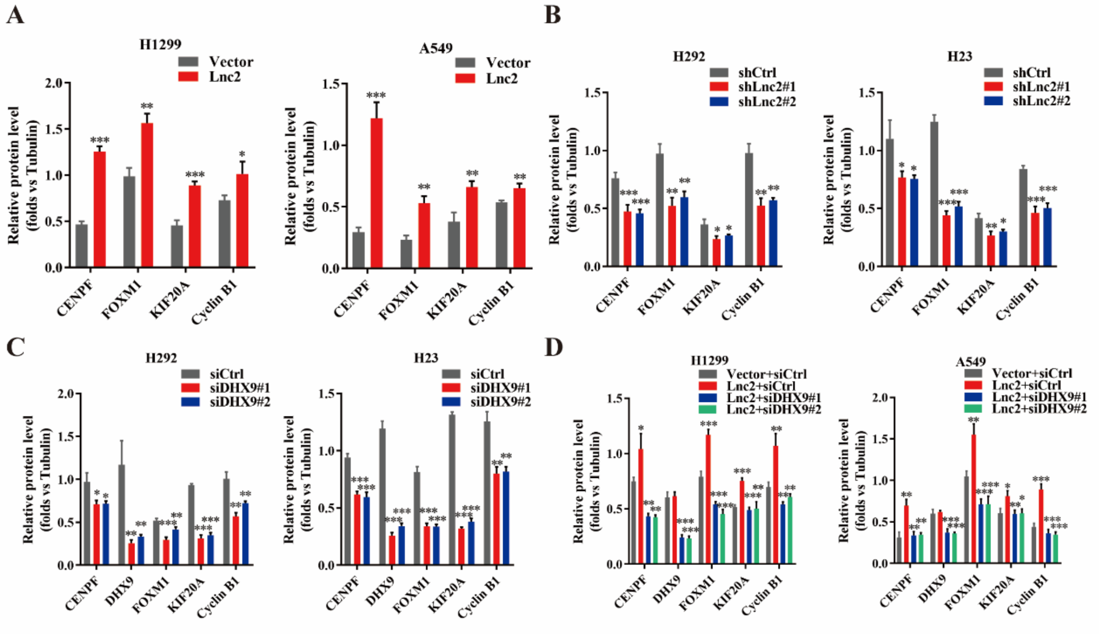
A
H1299
Vector
Lnc2
A549
Relative protein level (folds vs Tubulin)
CENPF
FOXM1
KIF20A
Cyclin B1
B
H292
H23
shCtrl
shLnc2#1
shLnc2#2
C
siCtrl
siDHX9#1
siDHX9#2
DHX9
D
Vector+siCtrl
Lnc2+siCtrl
Lnc2+siDHX9#1
Lnc2+siDHX9#2

Figure S7
