# Supplementary material for: Long noncoding RNA SH3PXD2A-AS1 promotes NSCLC proliferation and accelerates cell cycle progression by interacting with DHX9
Source: Cell Death Discov. 2022 Apr 11;8:192. doi: 10.1038/s41420-022-01004-6 (PMC9001675; doi:10.1038/s41420-022-01004-6)
Supplement: Supplementary file 7 — original western blots [file 41420_2022_1004_MOESM7_ESM.pdf]

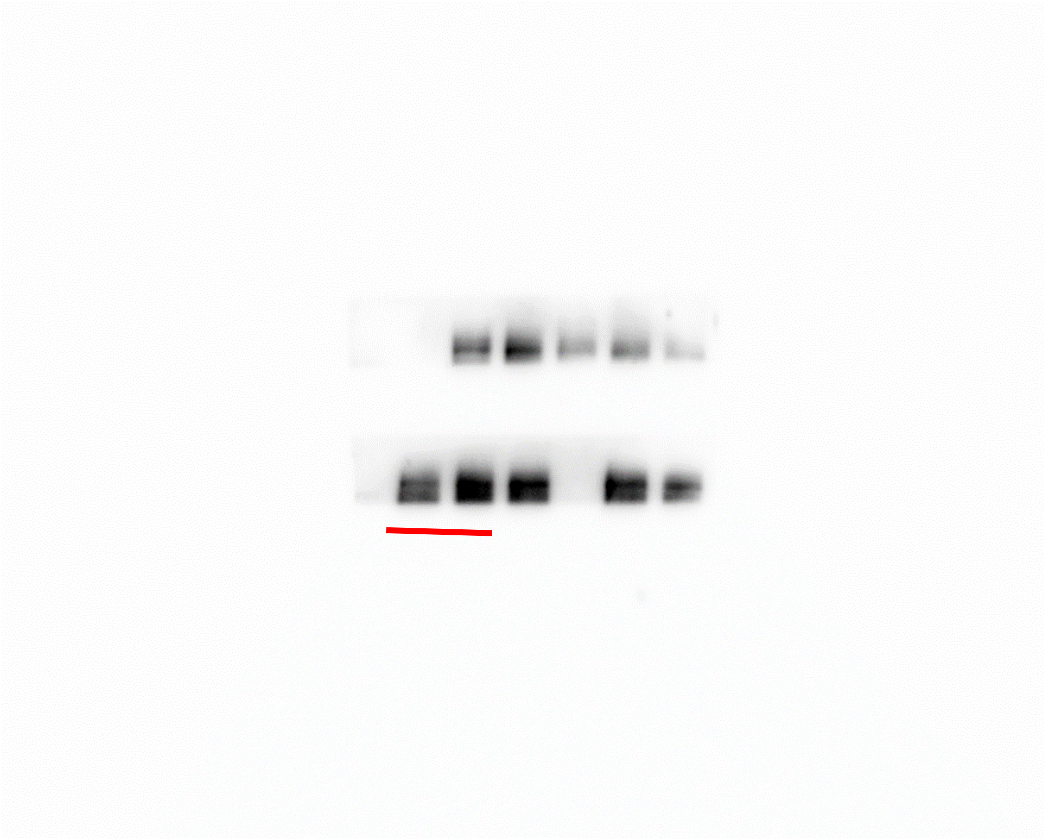

Fig 3D H1299 CENPF

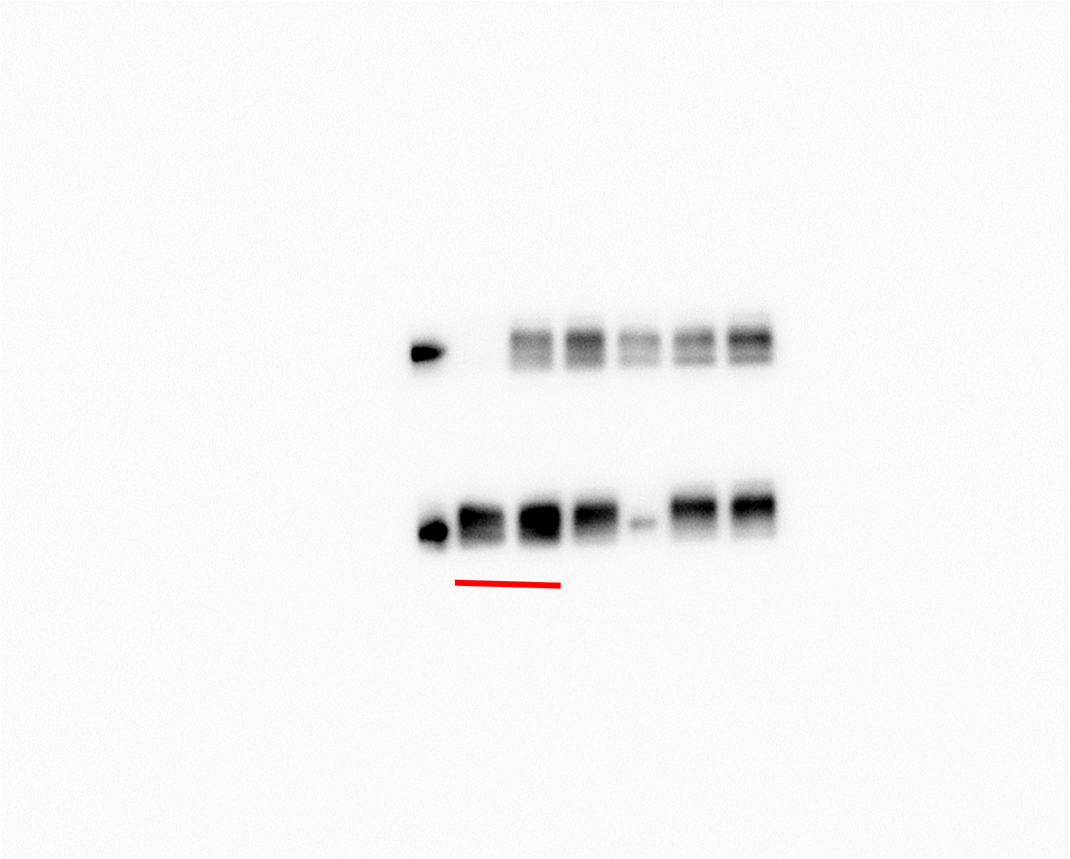

Fig 3D H1299 FOXM1

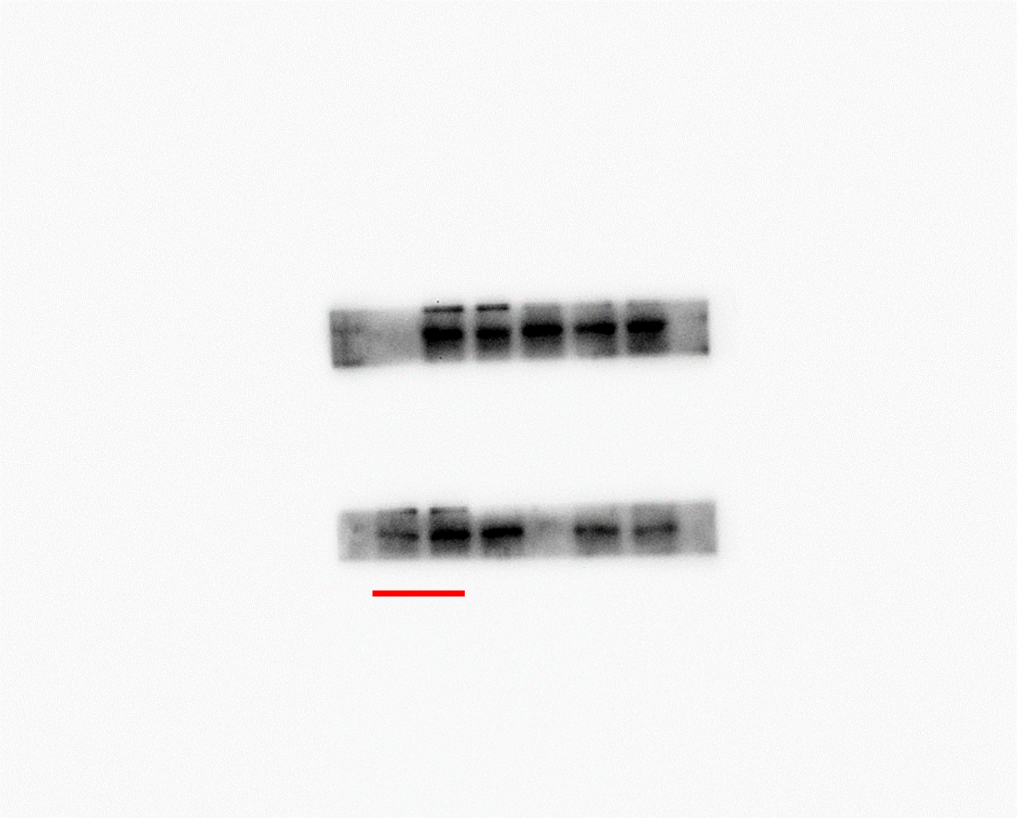

Fig 3D H1299 KIF20A

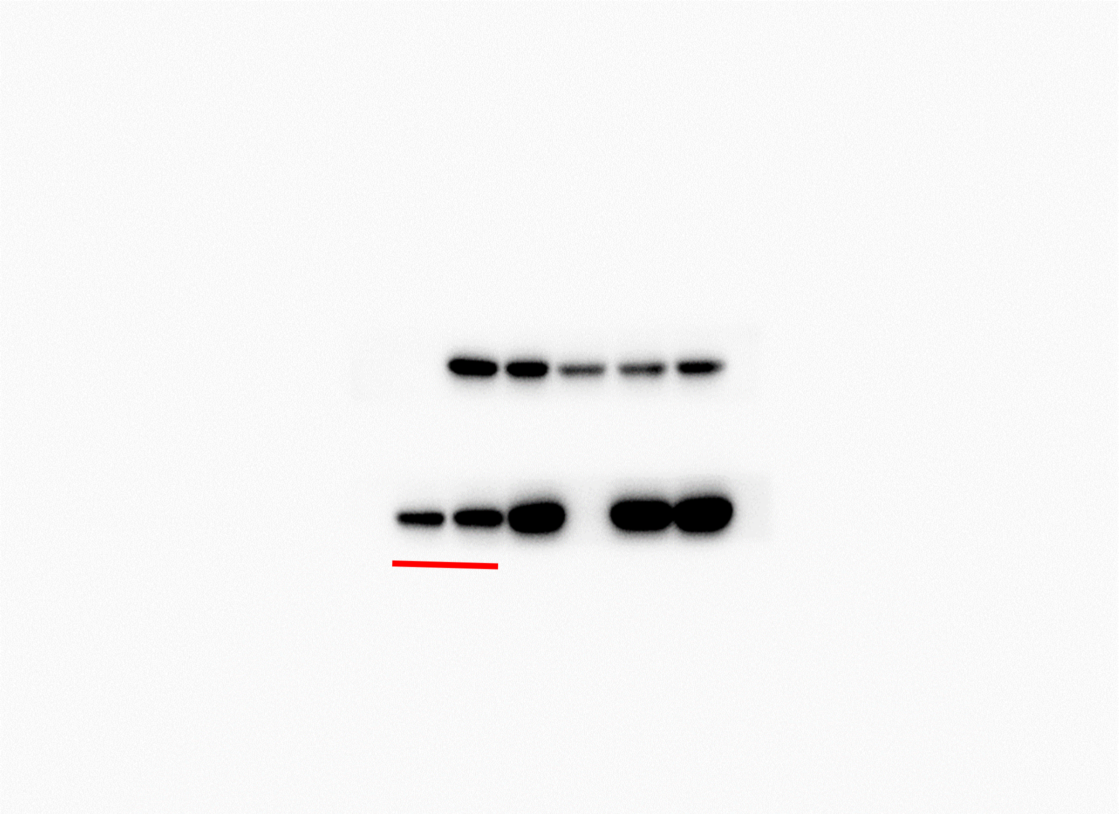

Fig 3D H1299 CCNB1

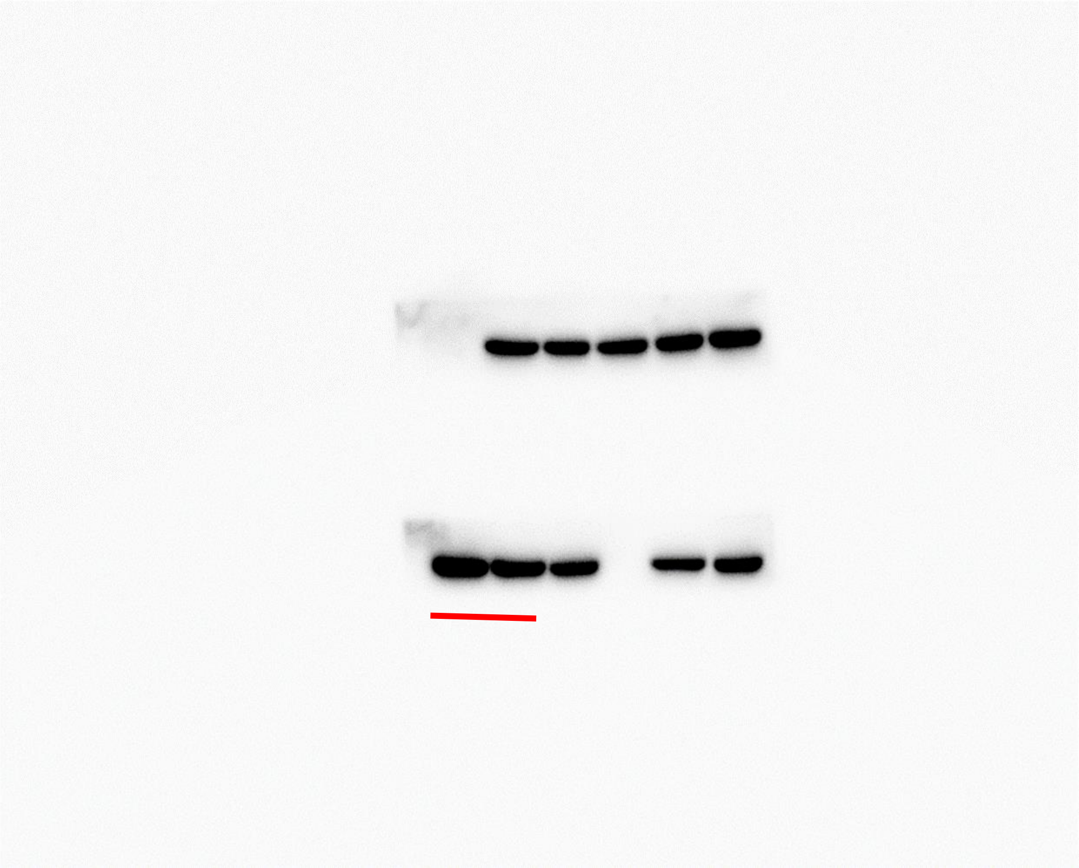

Fig 3D H1299 TUBULIN

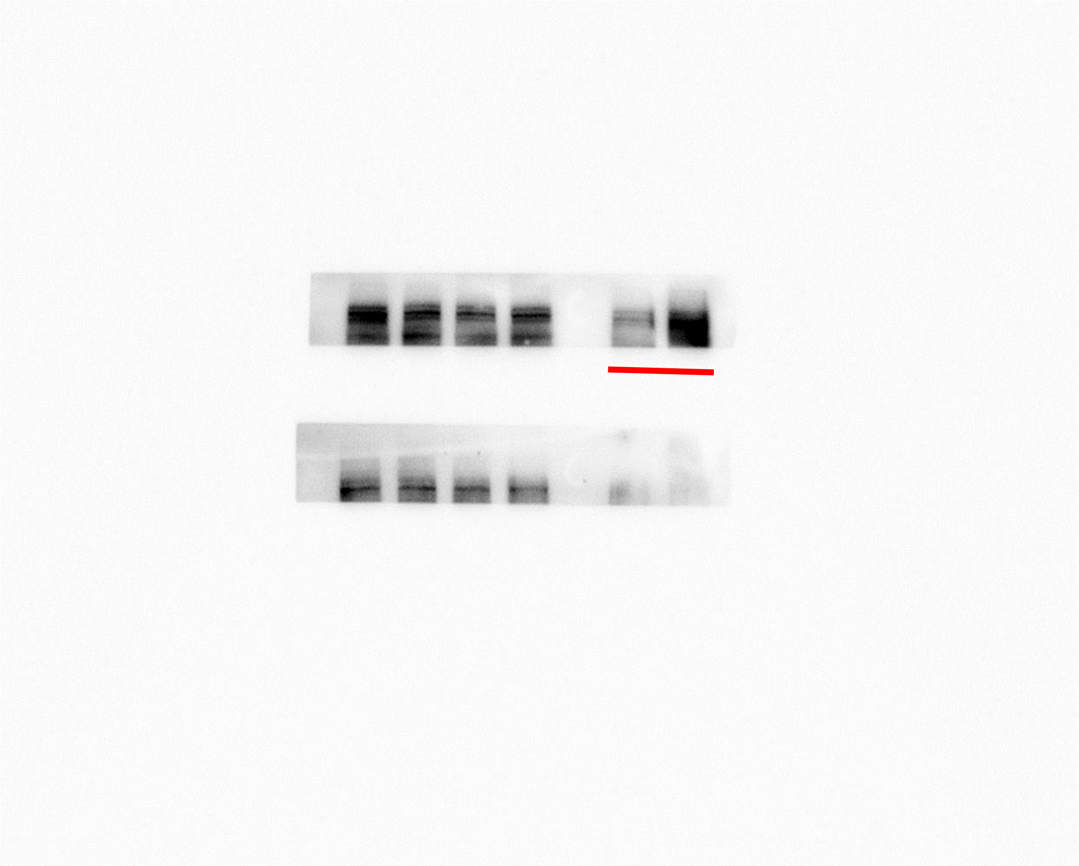

Fig 3D A549 CENPF

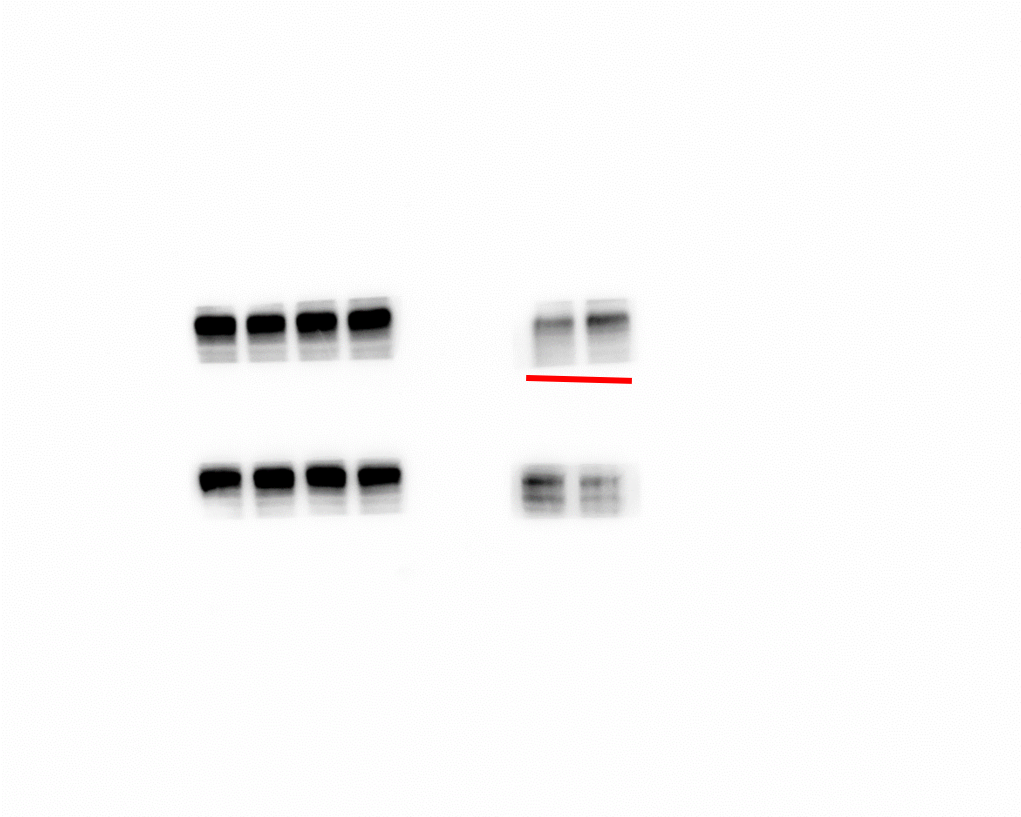

Fig 3D A549 FOXM1

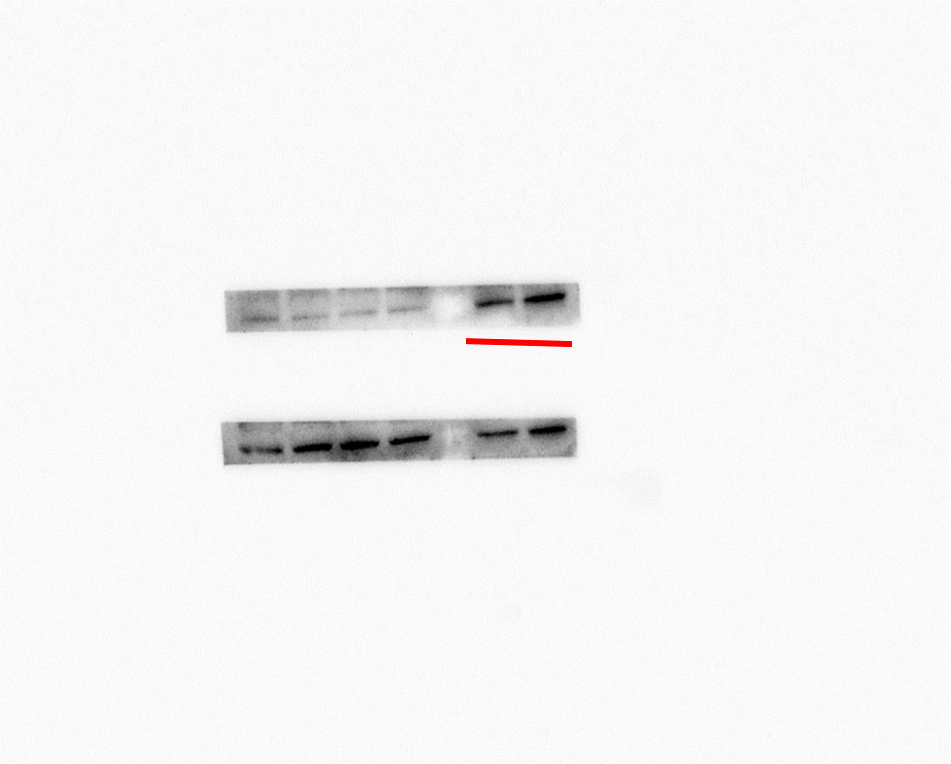

Fig 3D A549 KIF20A

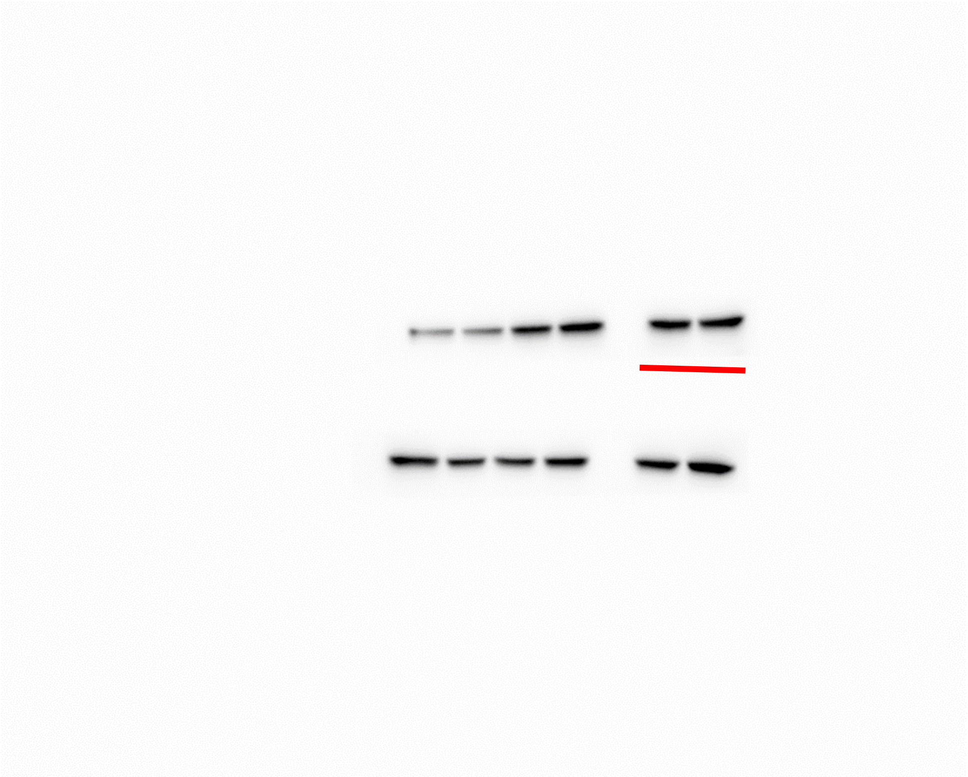

Fig 3D A549 CCNB1

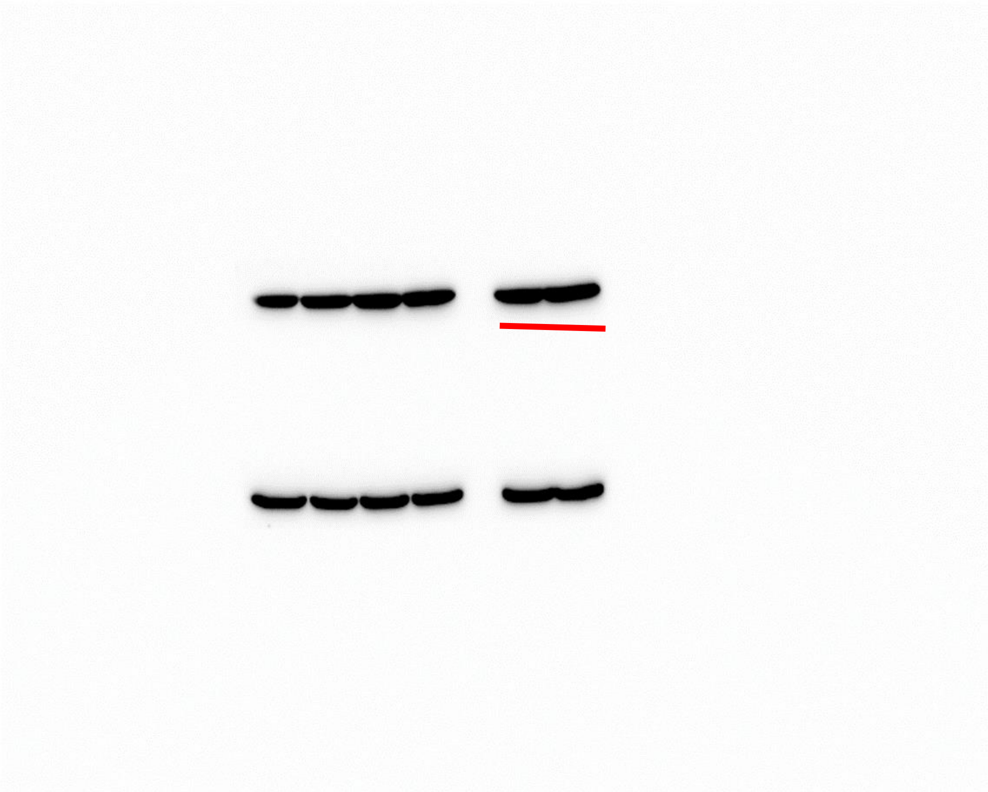

Fig 3D A549 TUBULIN

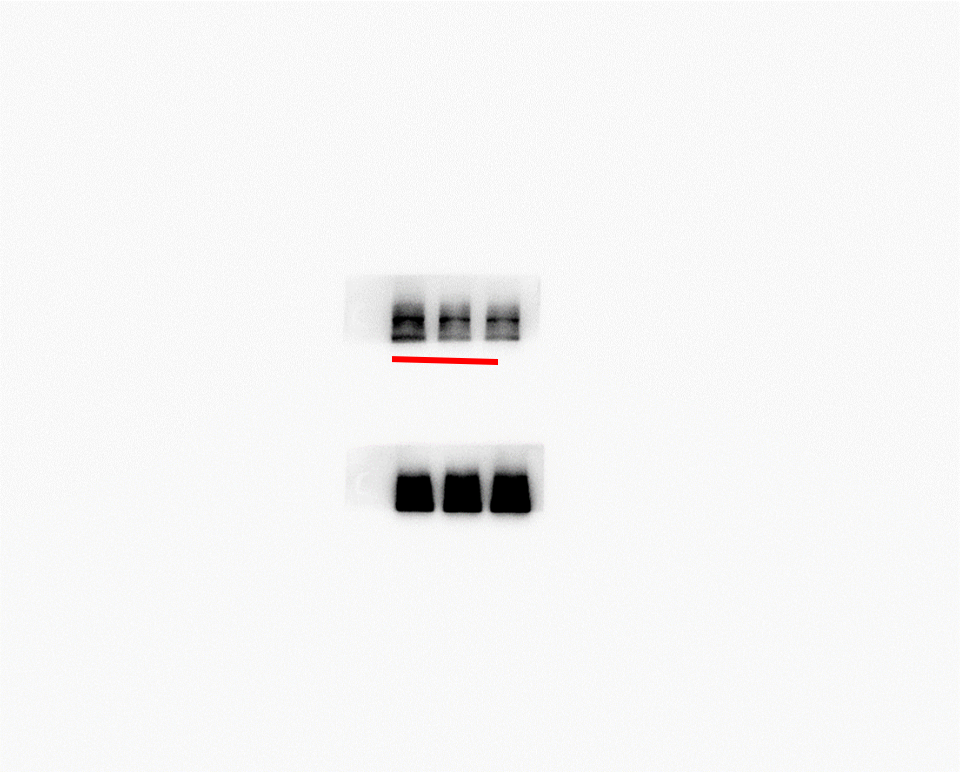

Fig 3E H292 CENPF

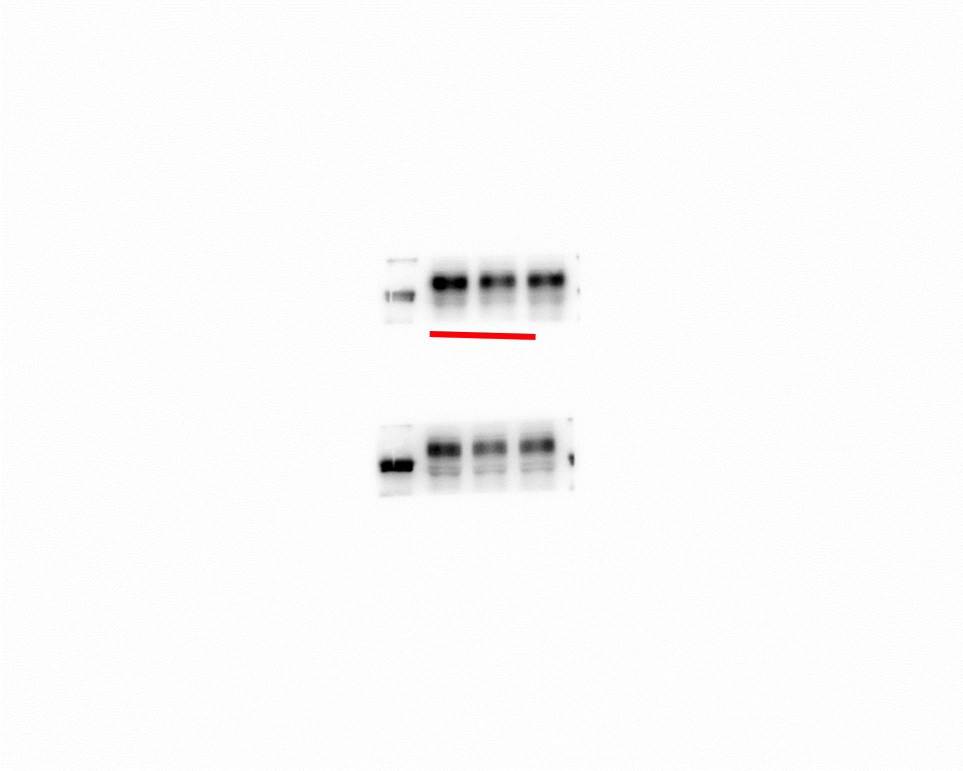

Fig 3E H292 FOXM1

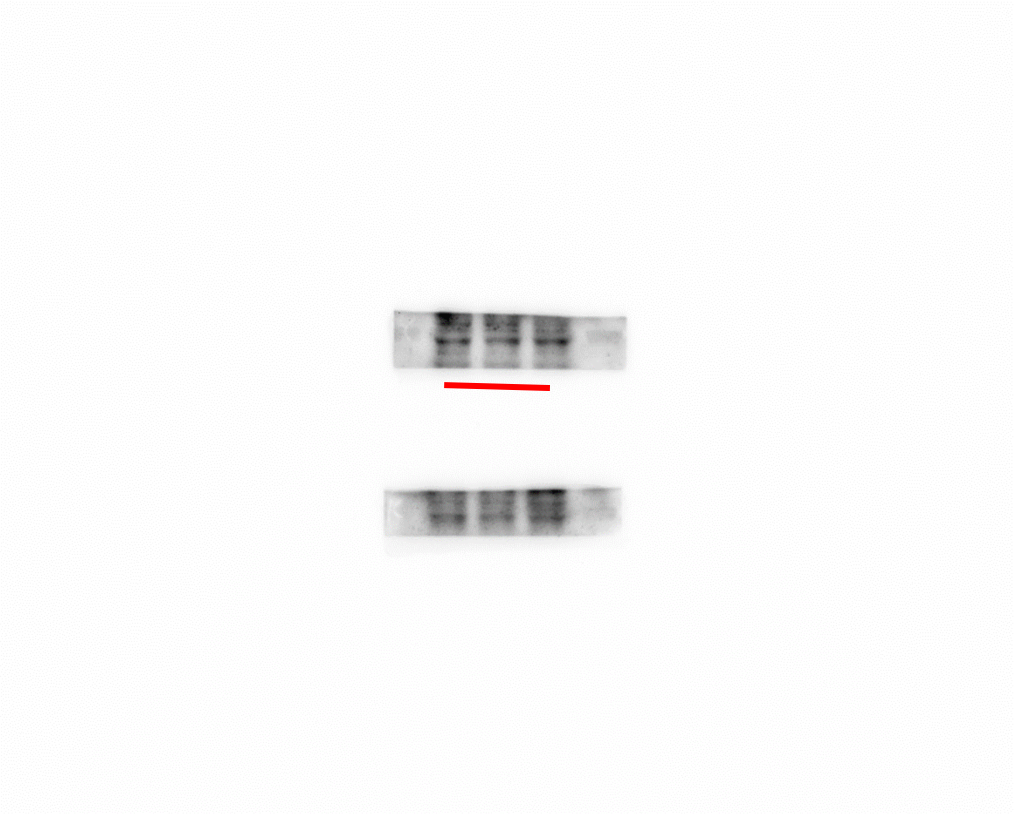

Fig 3E H292 KIF20A

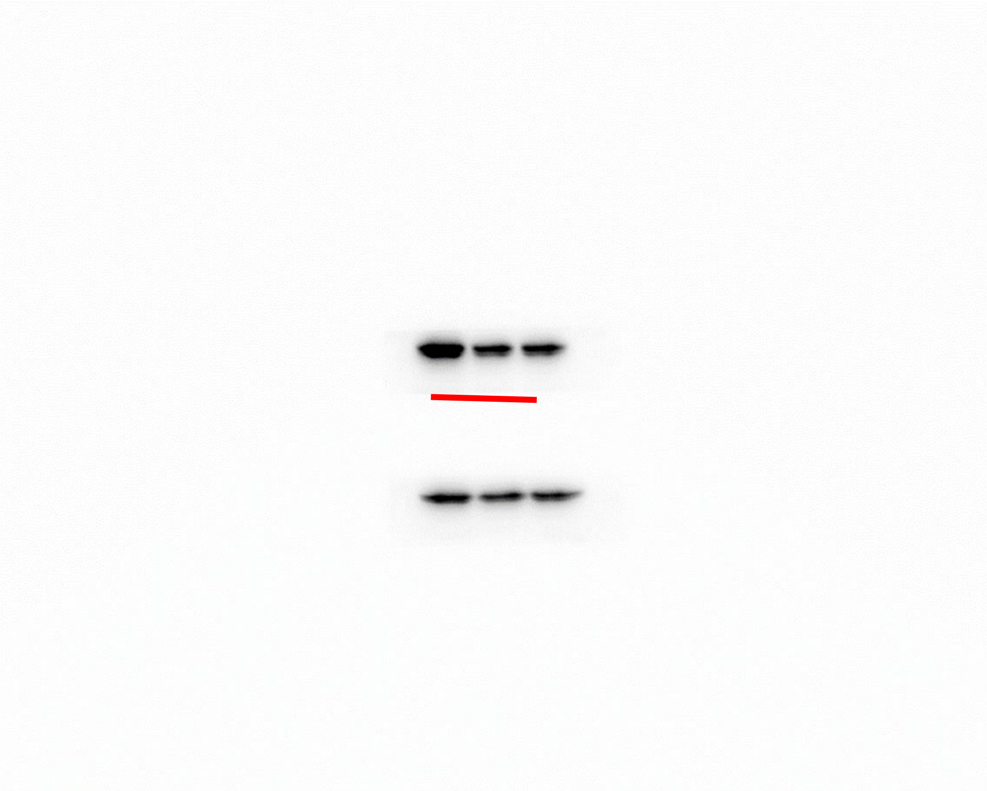

Fig 3E H292 CCNB1

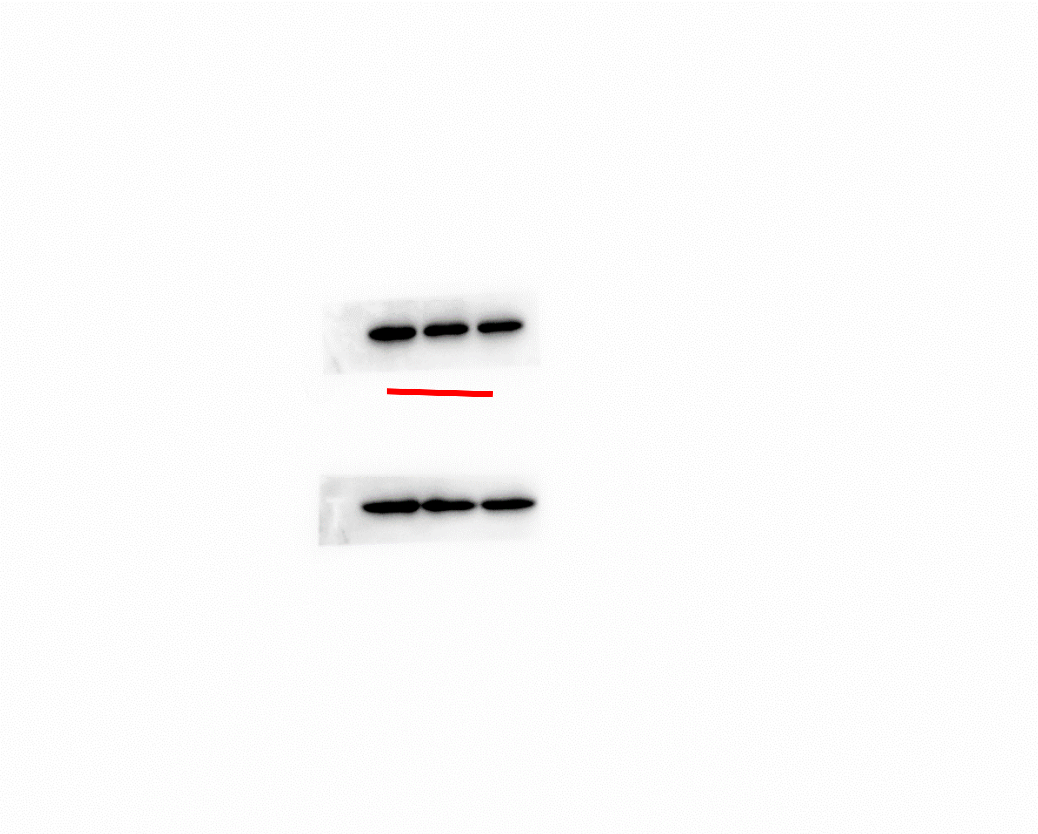

Fig 3E H292 TUBULIN

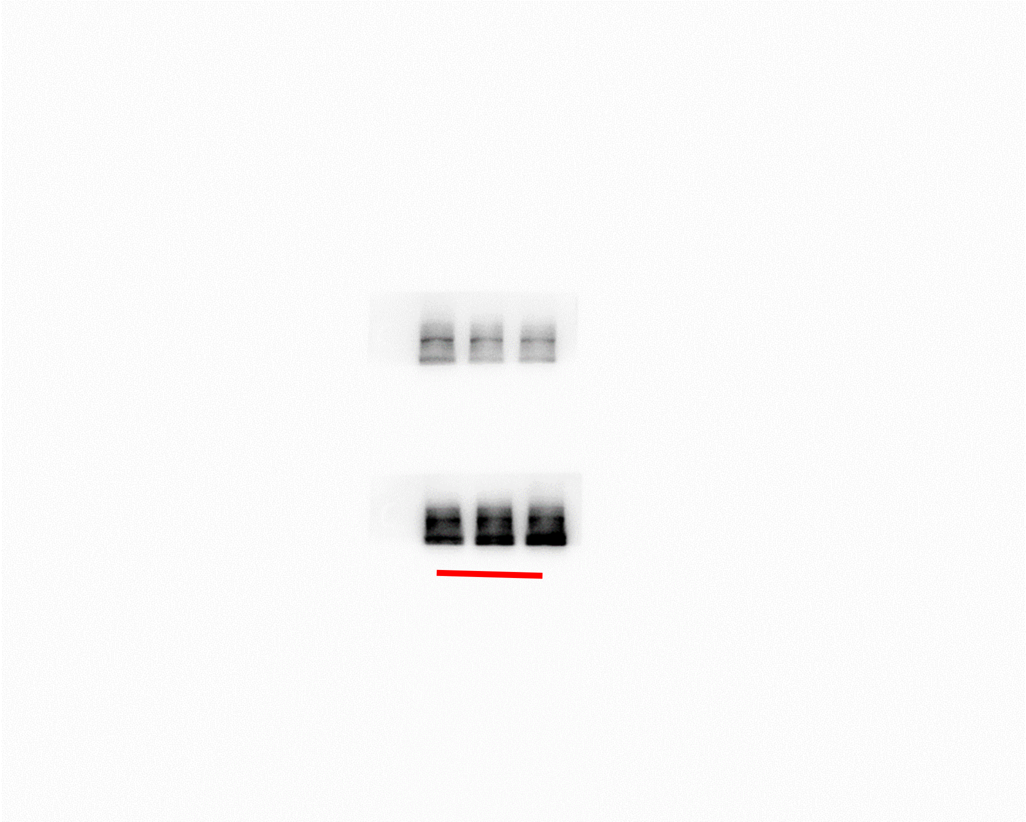

Fig 3E H23 CENPF

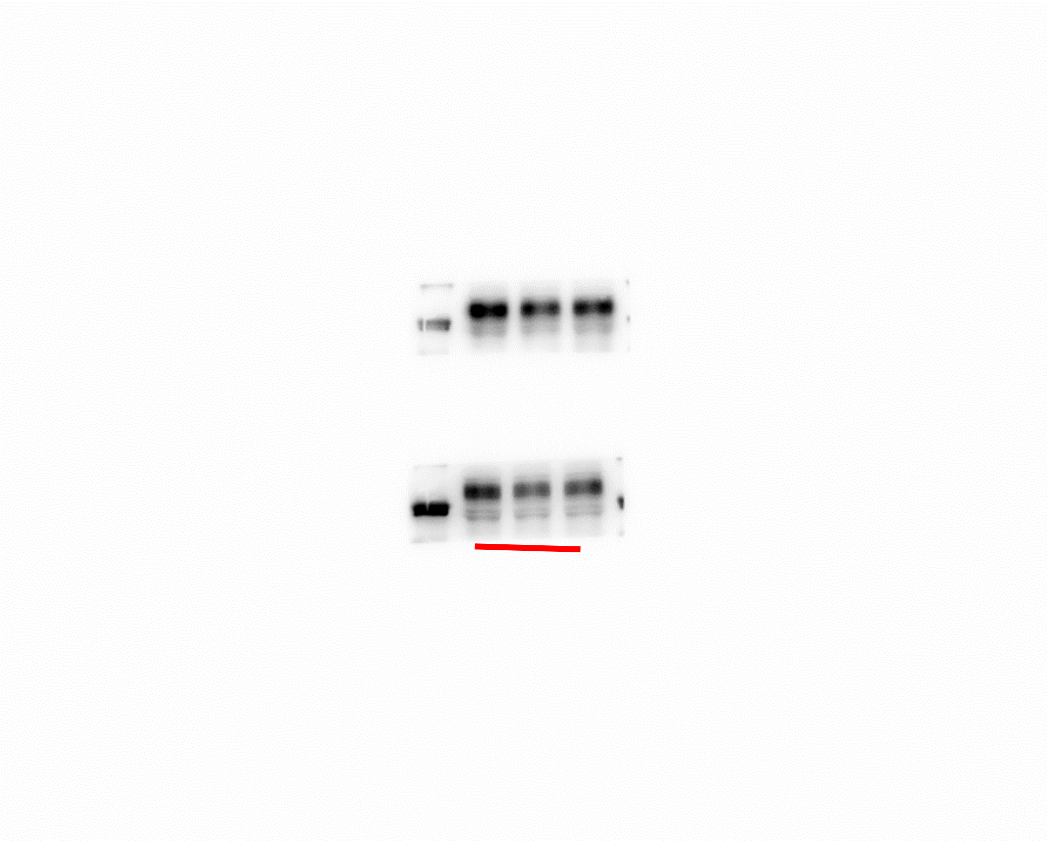

Fig 3E H23 FOXM1

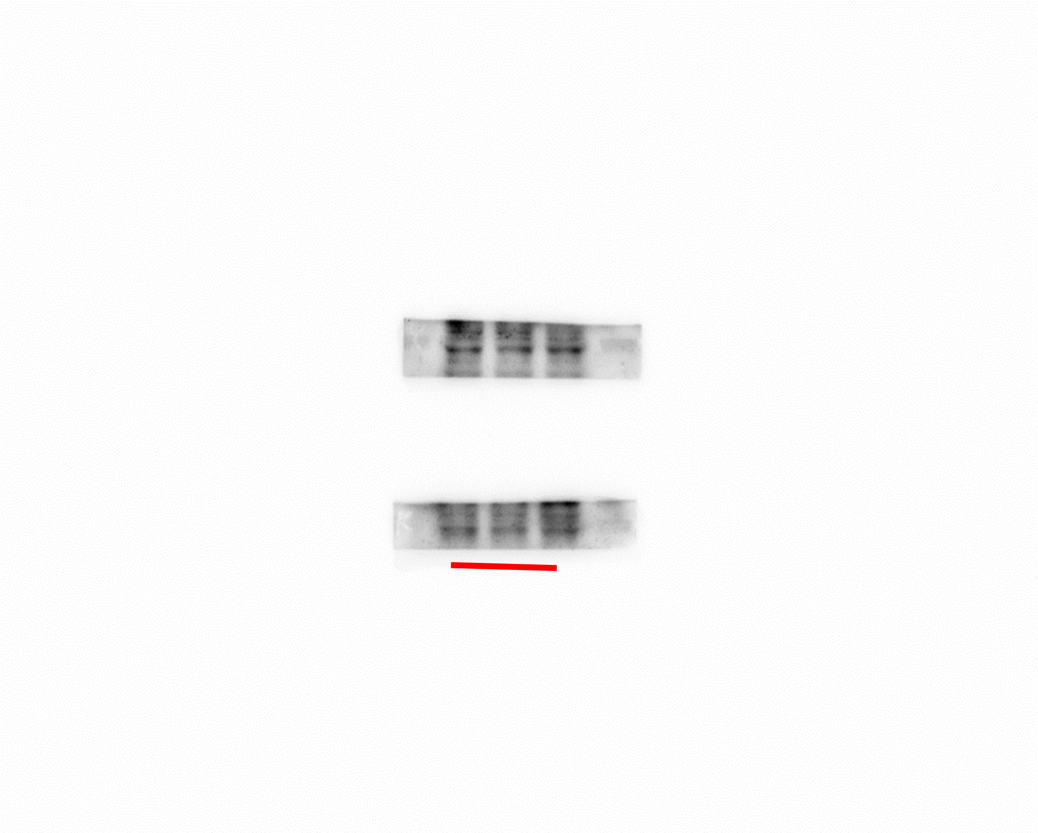

Fig 3E H23 KIF20A

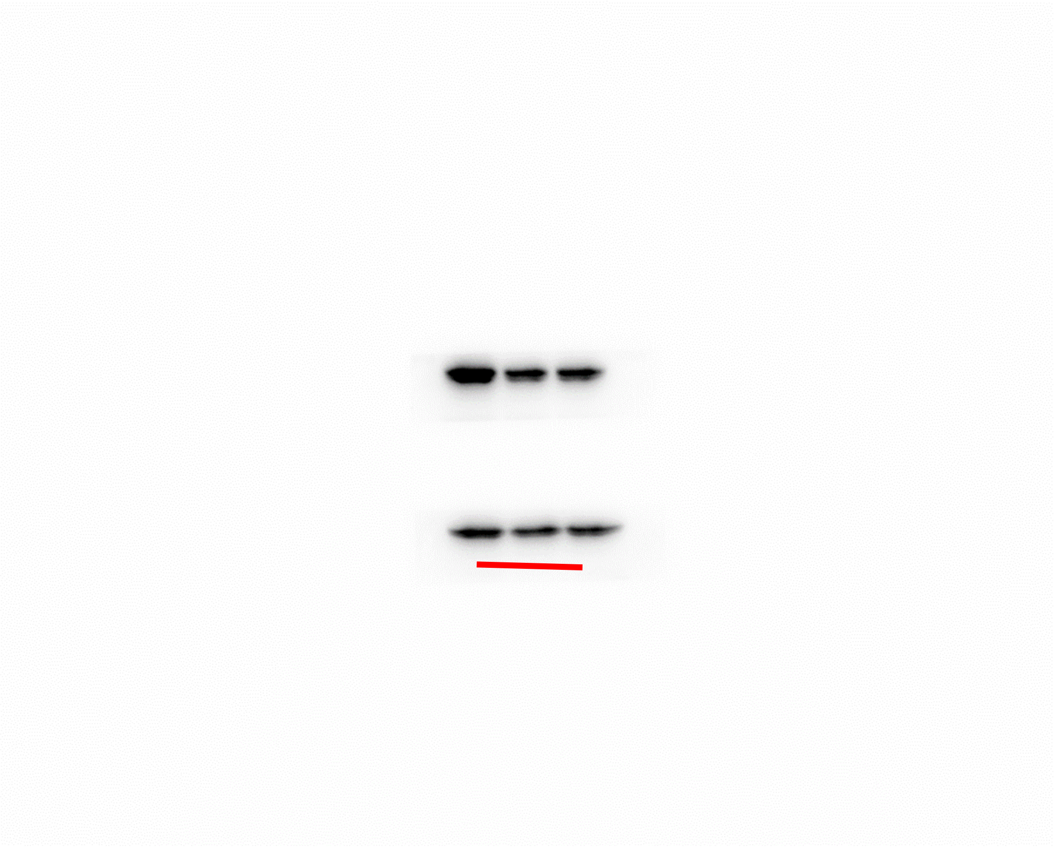

Fig 3E H23 CCNB1

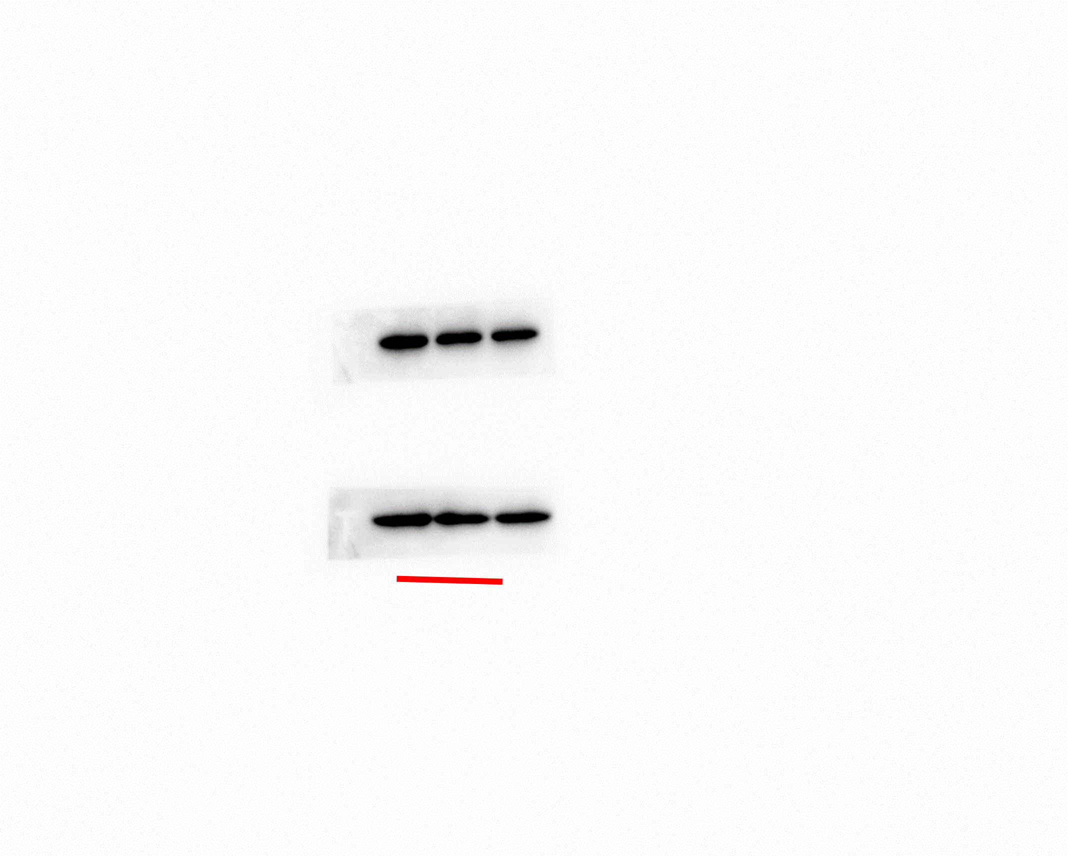

Fig 3E H23 TUBULIN

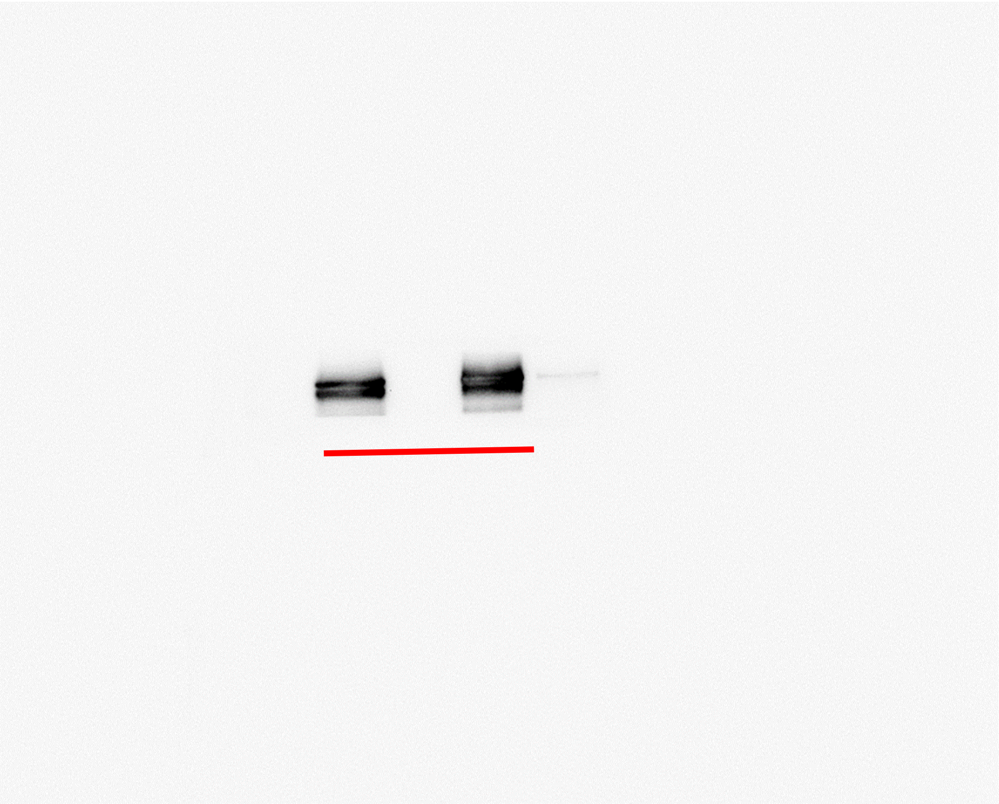

Fig 4B DHX9

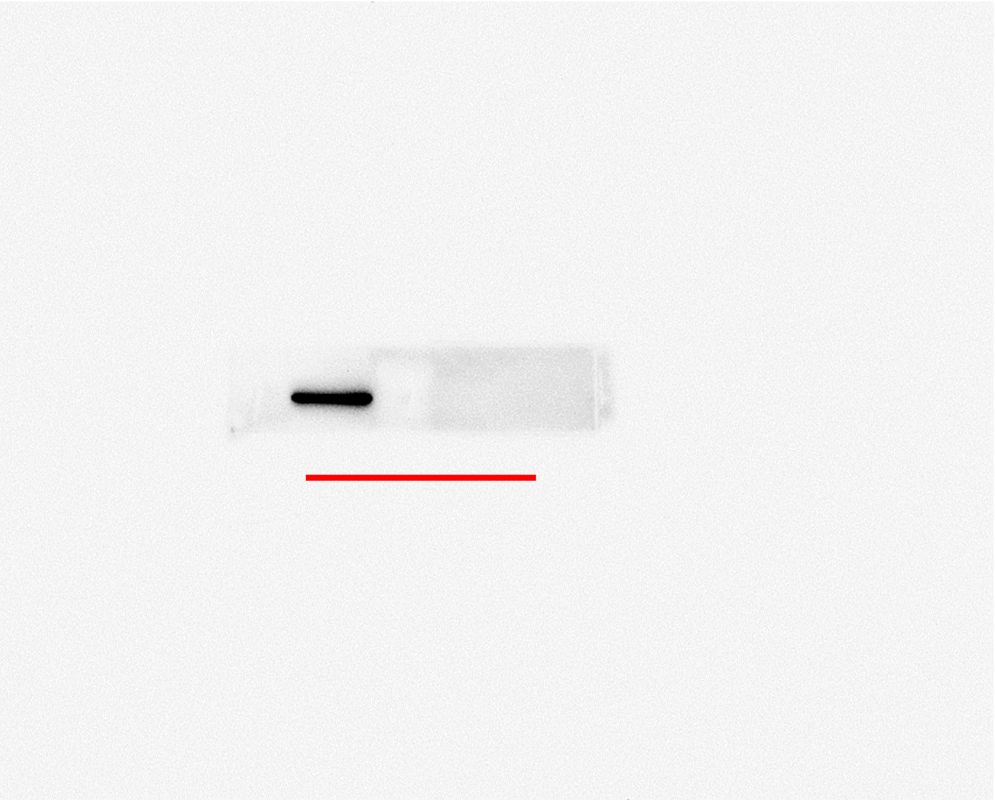

Fig 4B HSPA8

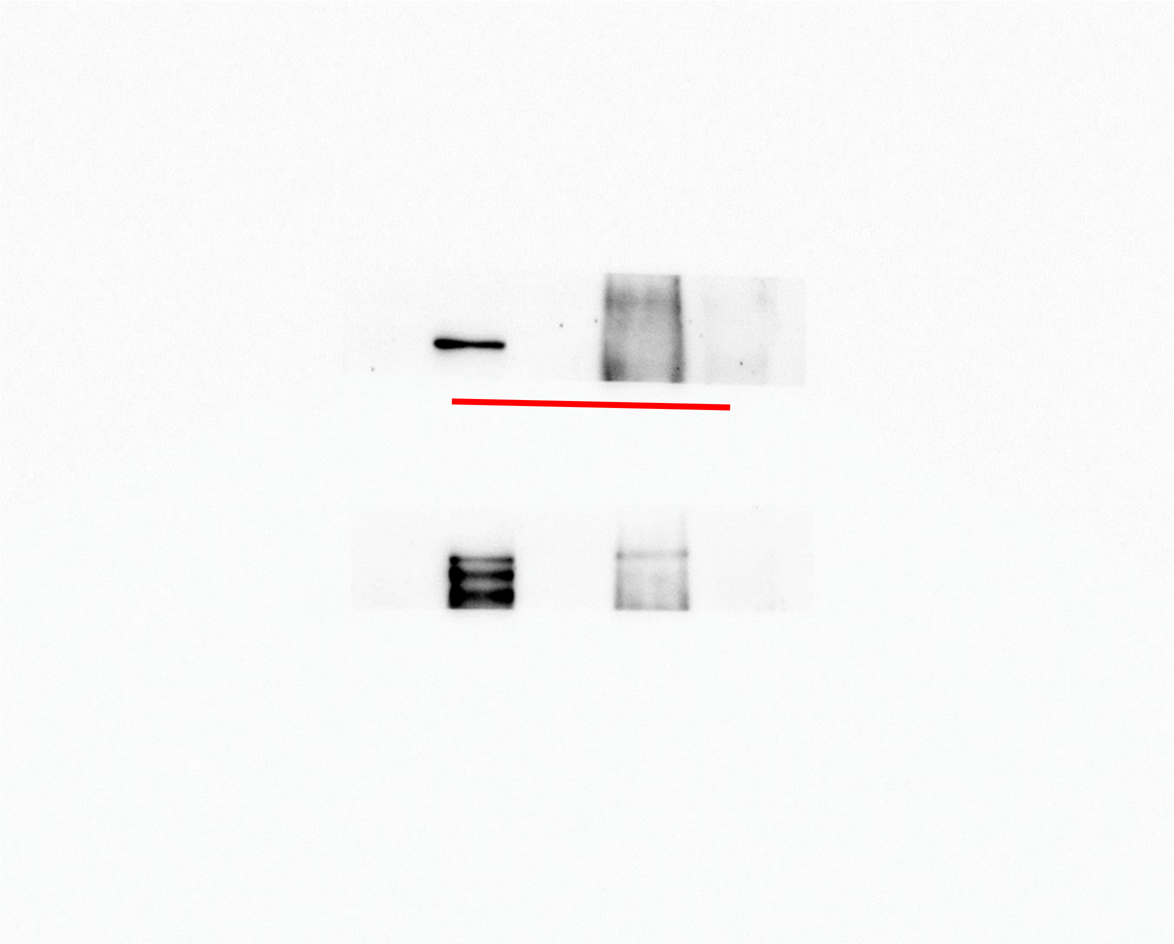

Fig 4B HSPA5

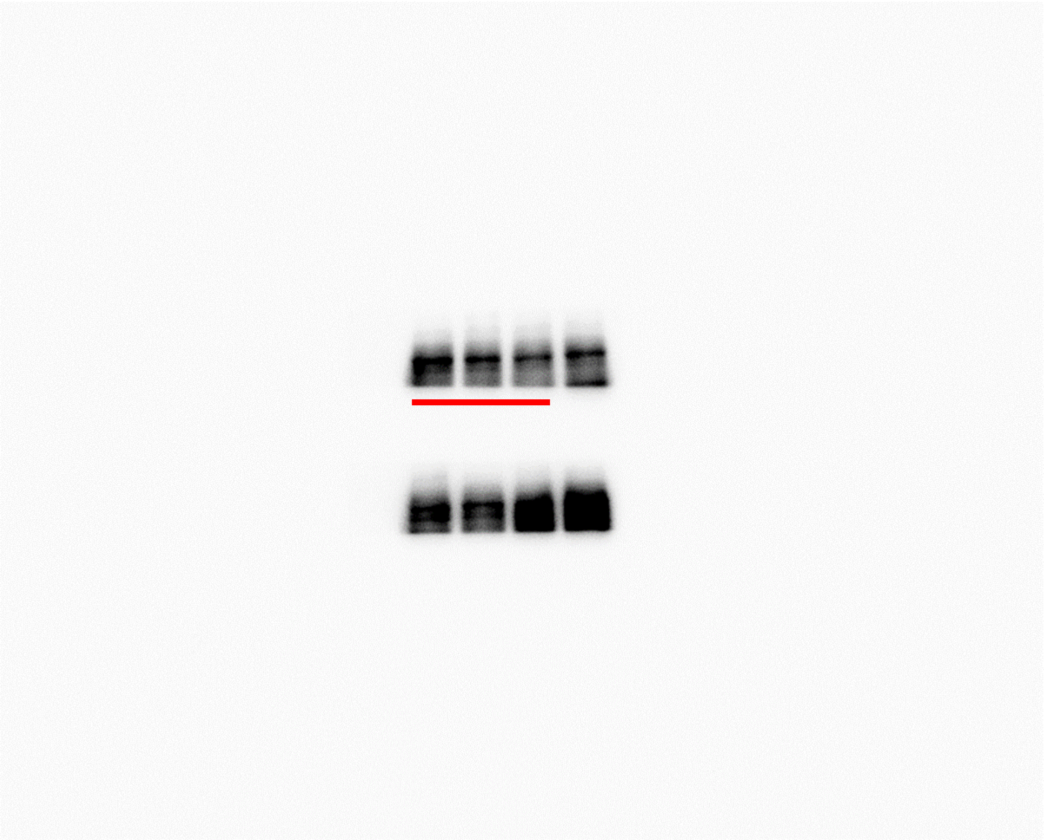

Fig 5A H292 CENPF

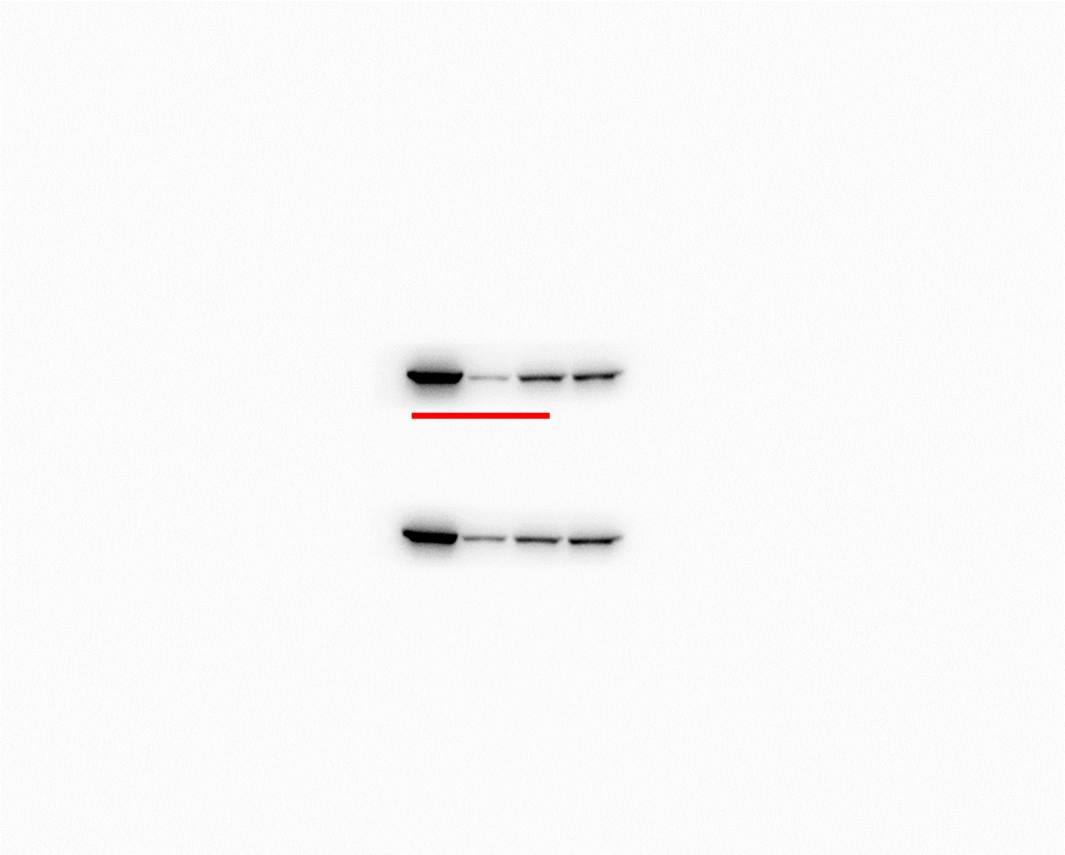

Fig 5A H292 DHX9

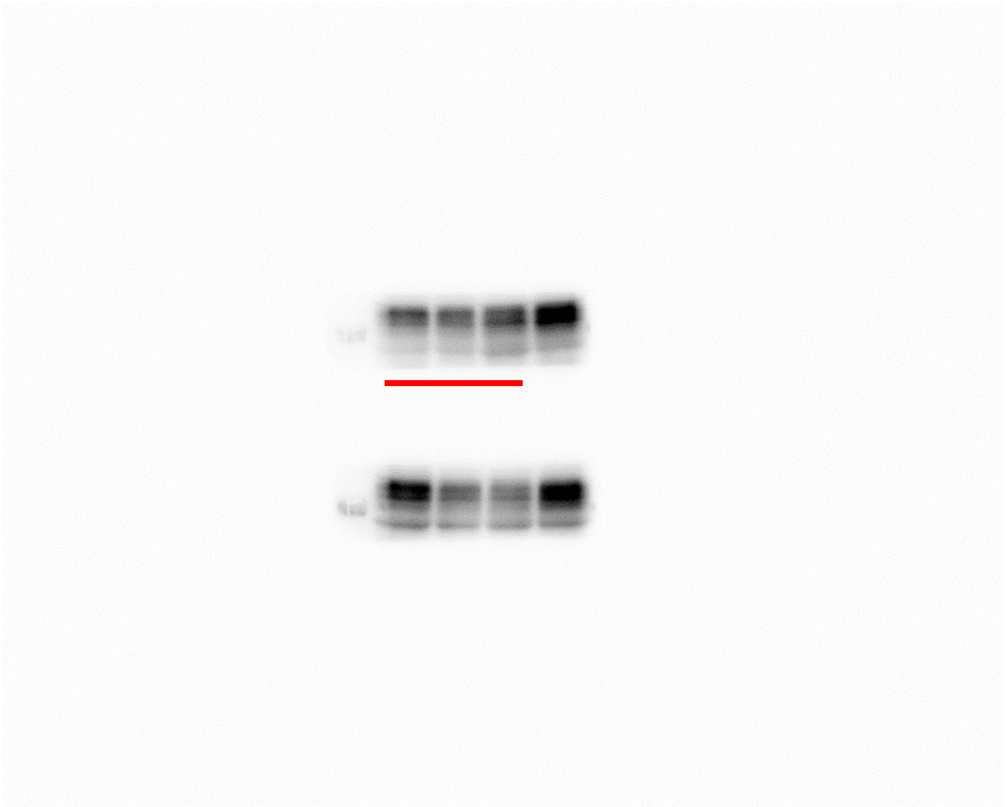

Fig 5A H292 FOXM1

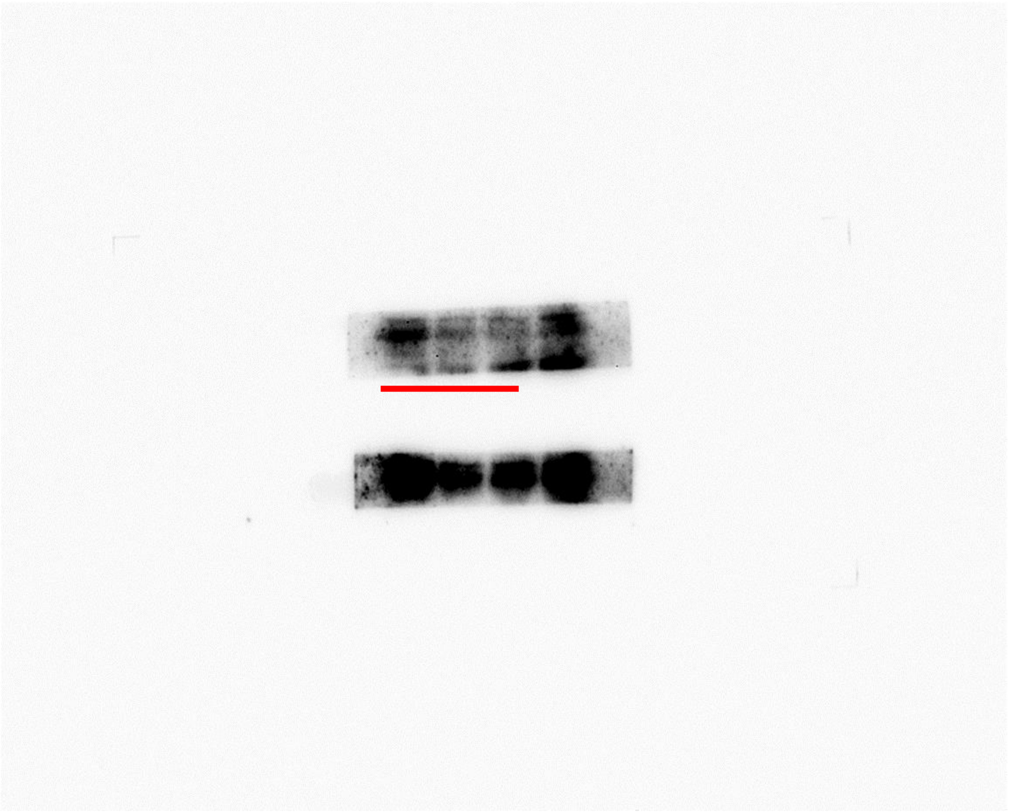

Fig 5A H292 KIF20A

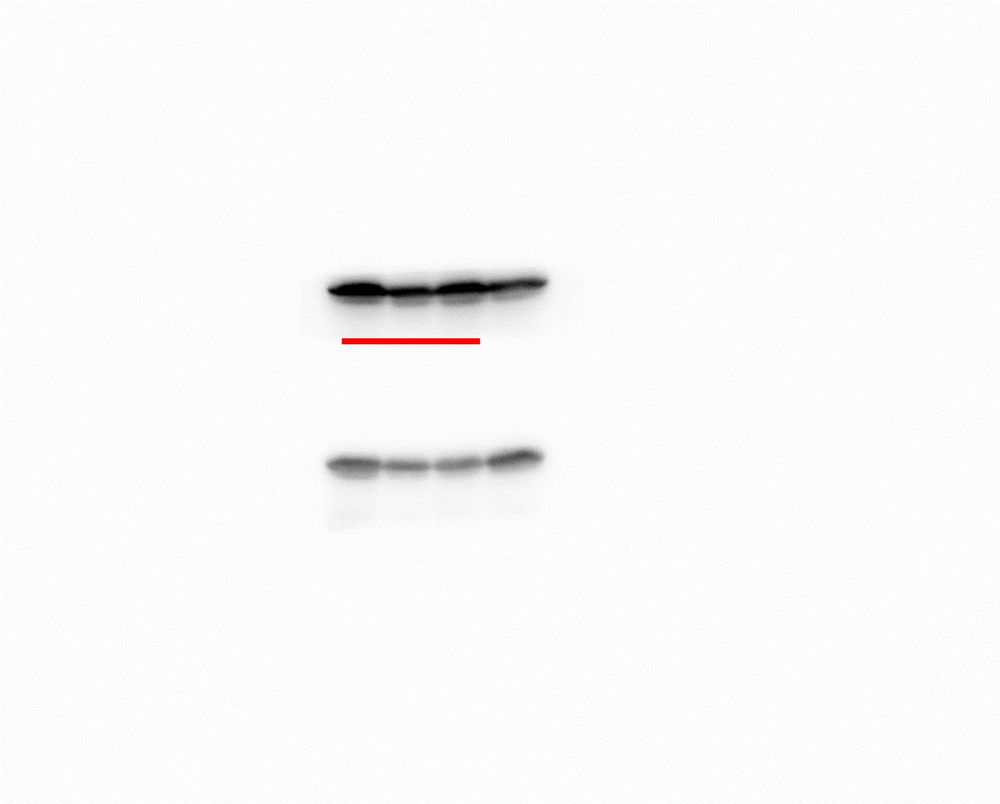

Fig 5A H292 CCNB1

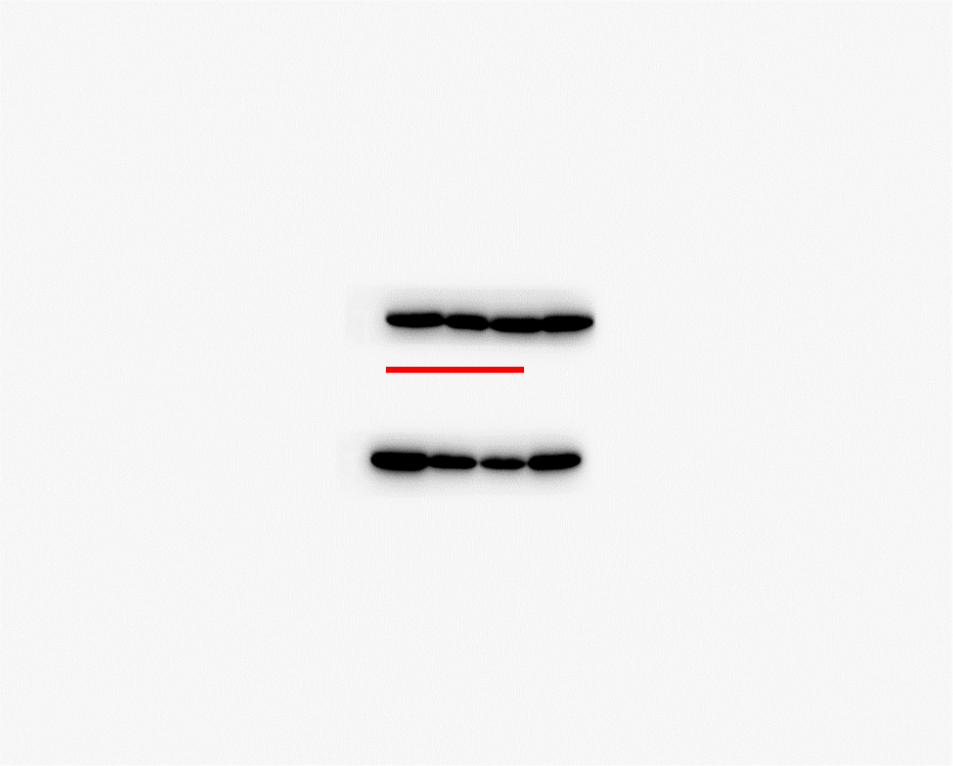

Fig 5A H292 TUBULIN

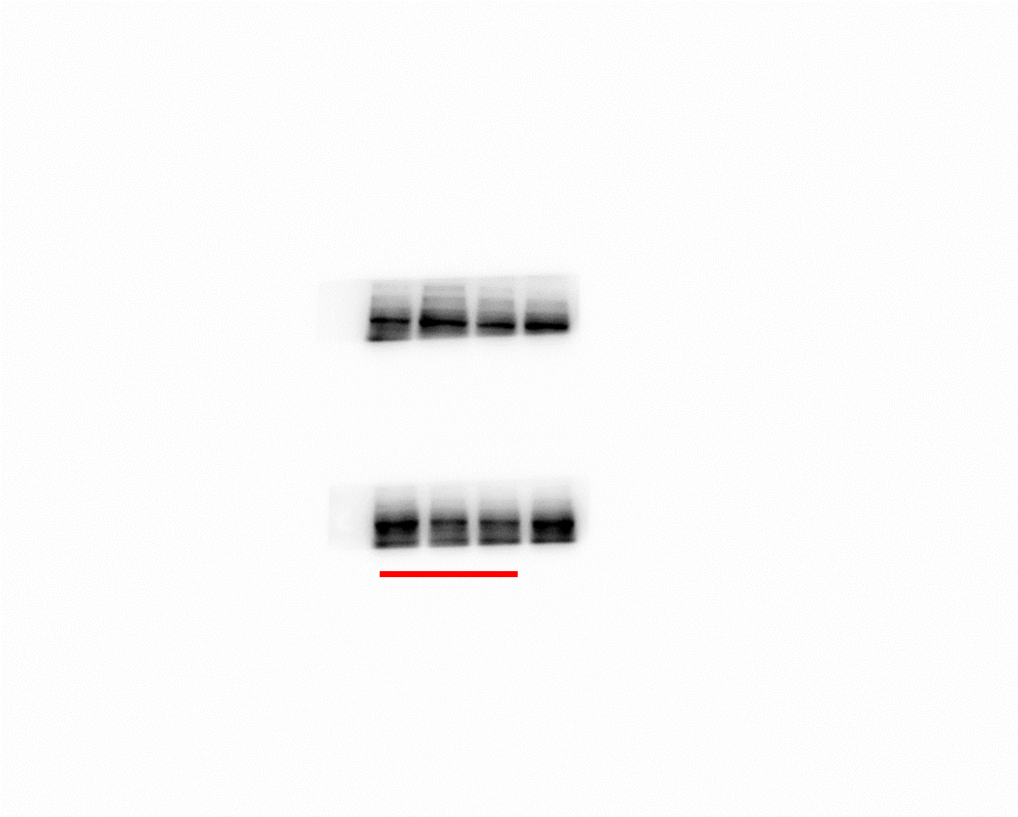

Fig 5A H23 CENPF

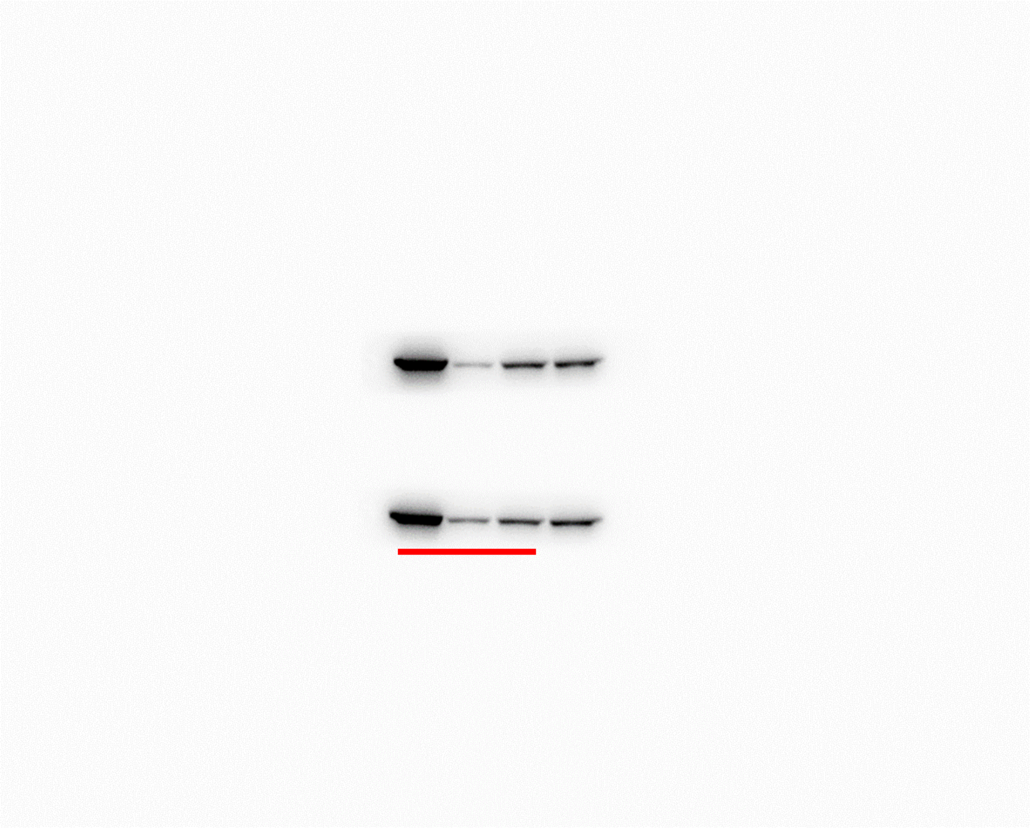

Fig 5A H23 DHX9

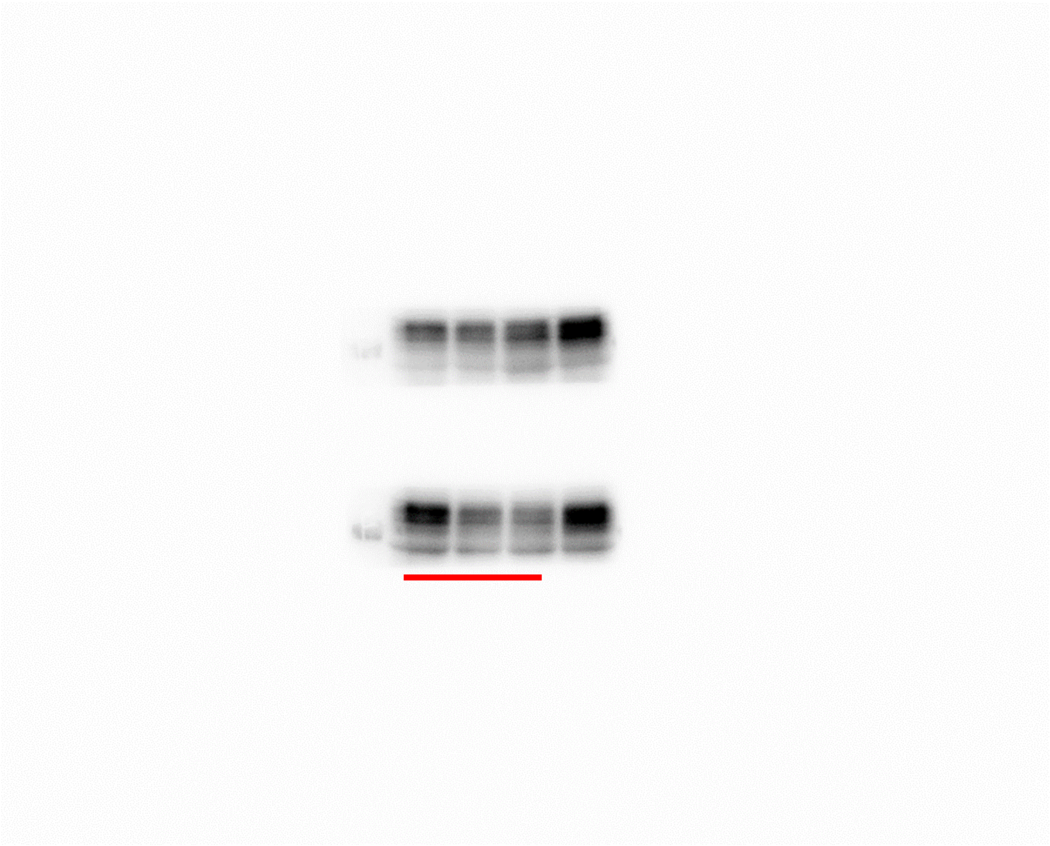

Fig 5A H23 FOXM1

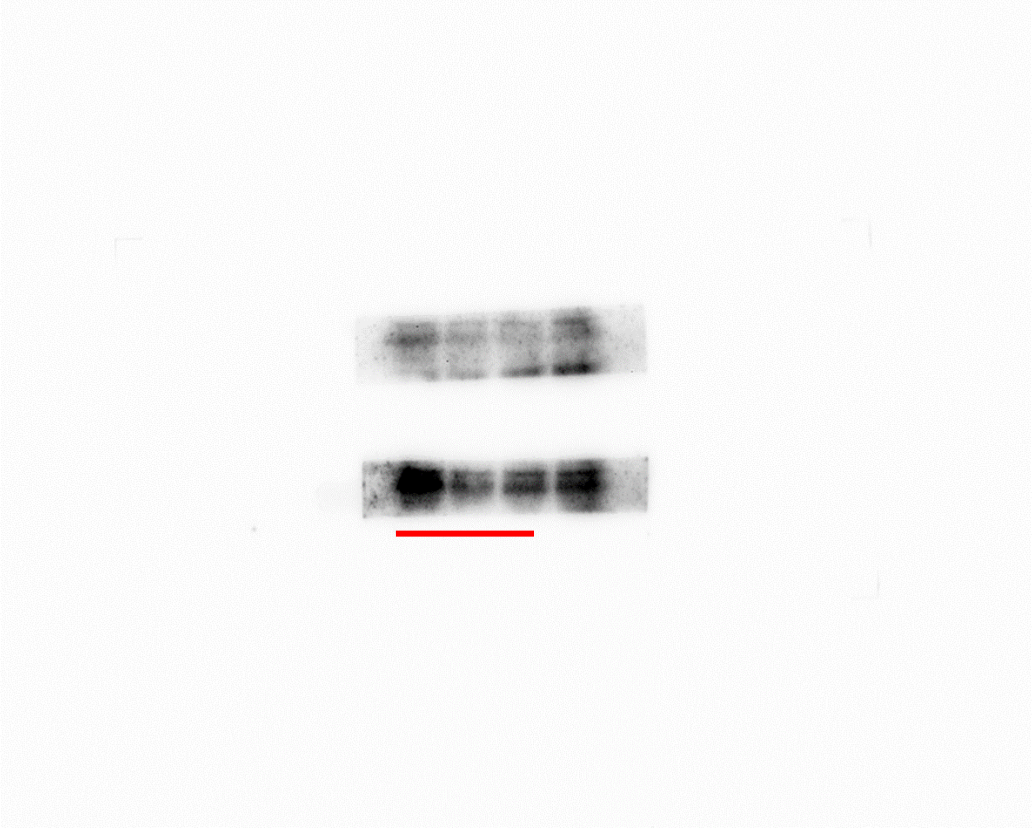

Fig 5A H23 KIF20A

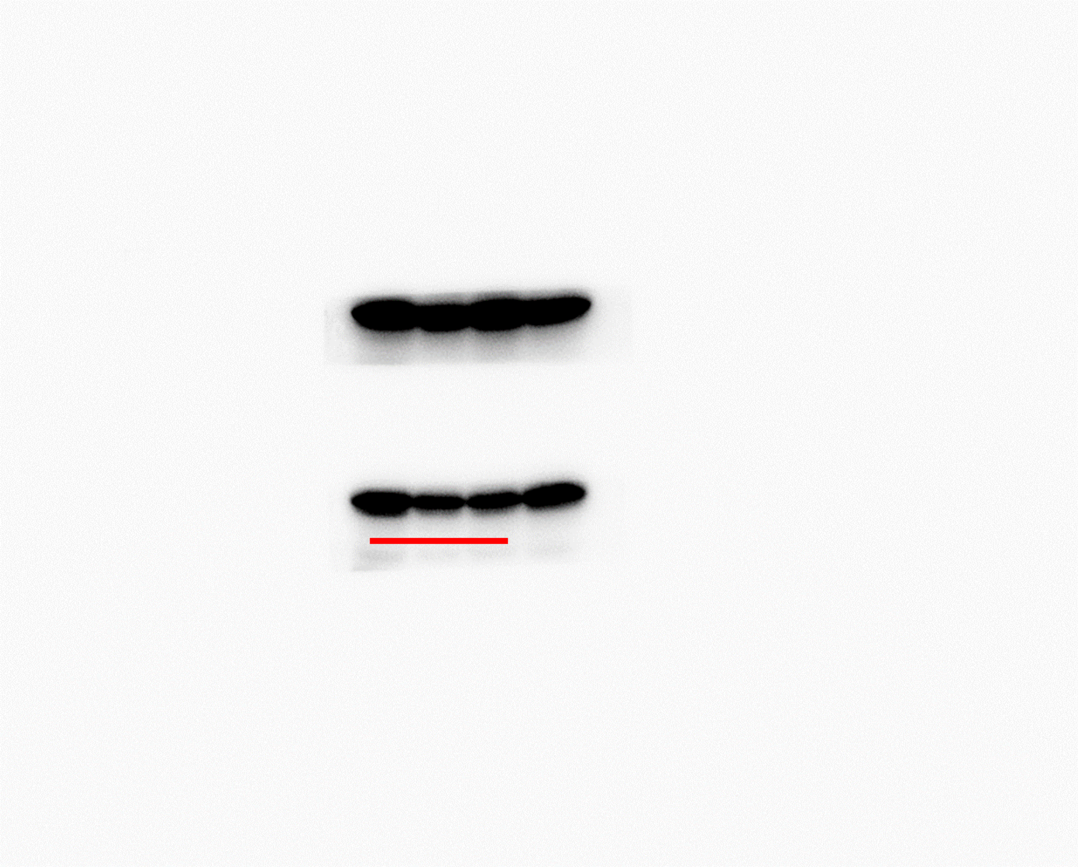

Fig 5A H23 CCNB1

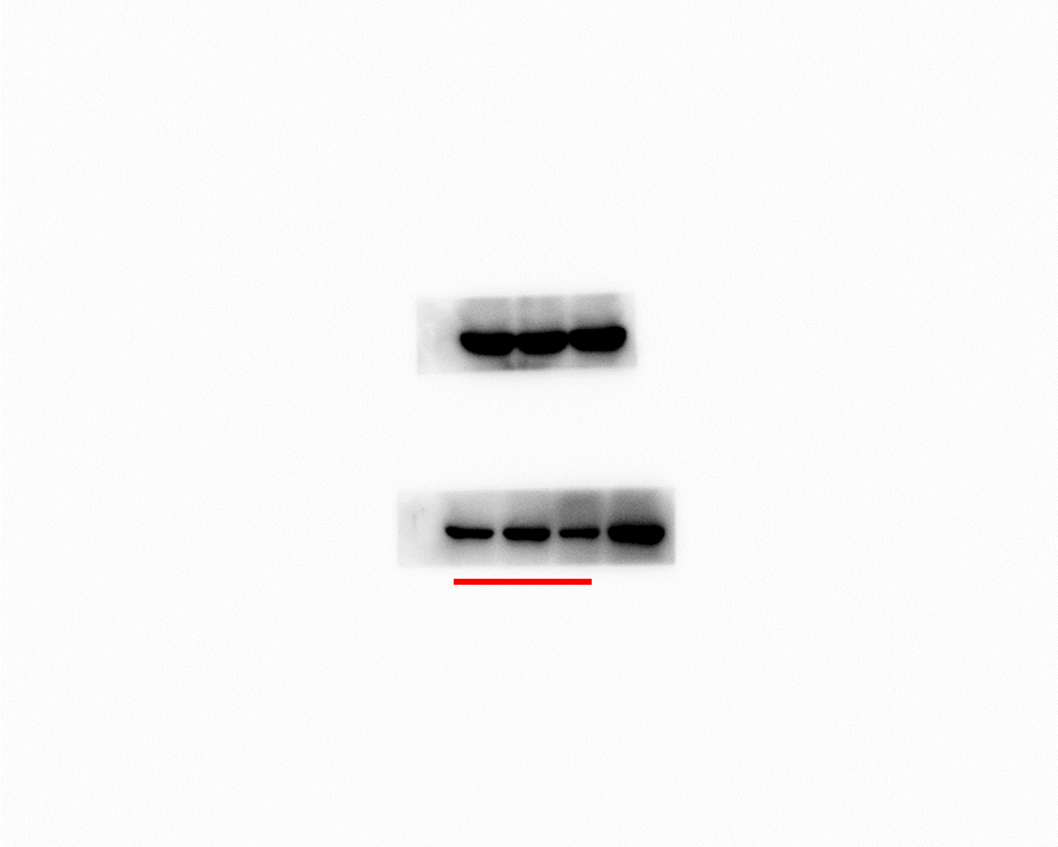

Fig 5A H23 TUBULIN

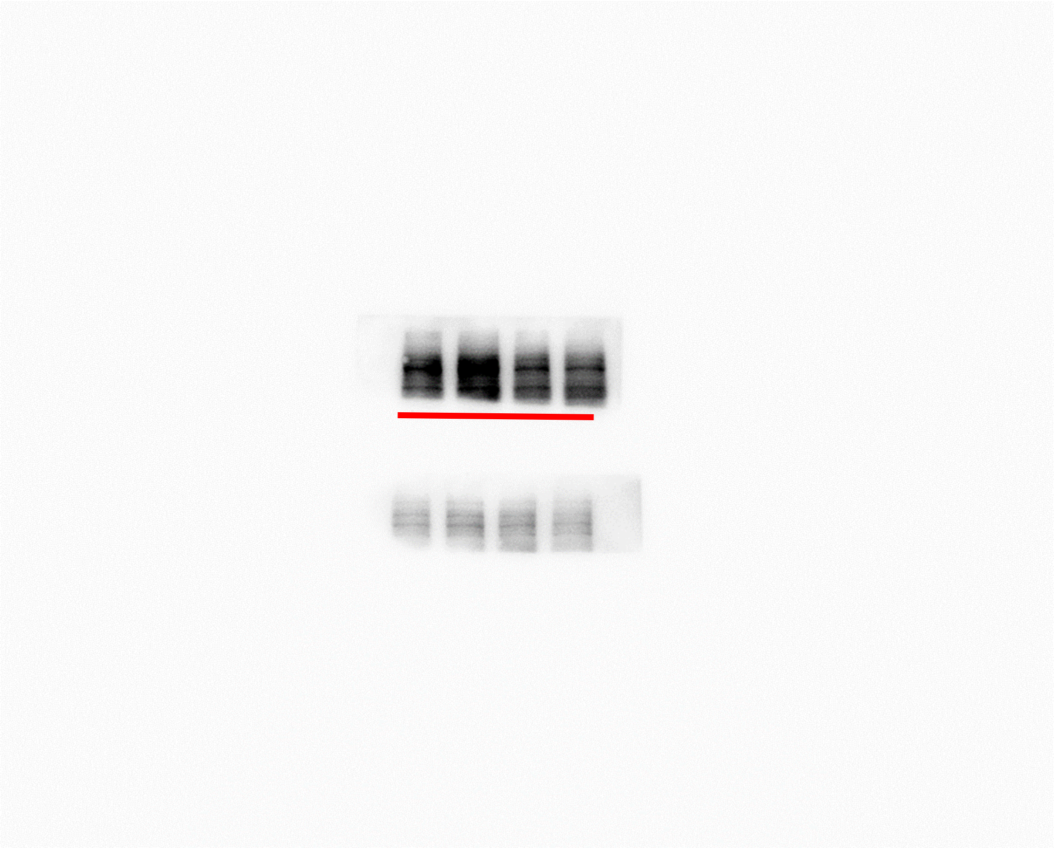

Fig 6A H1299 CENPF

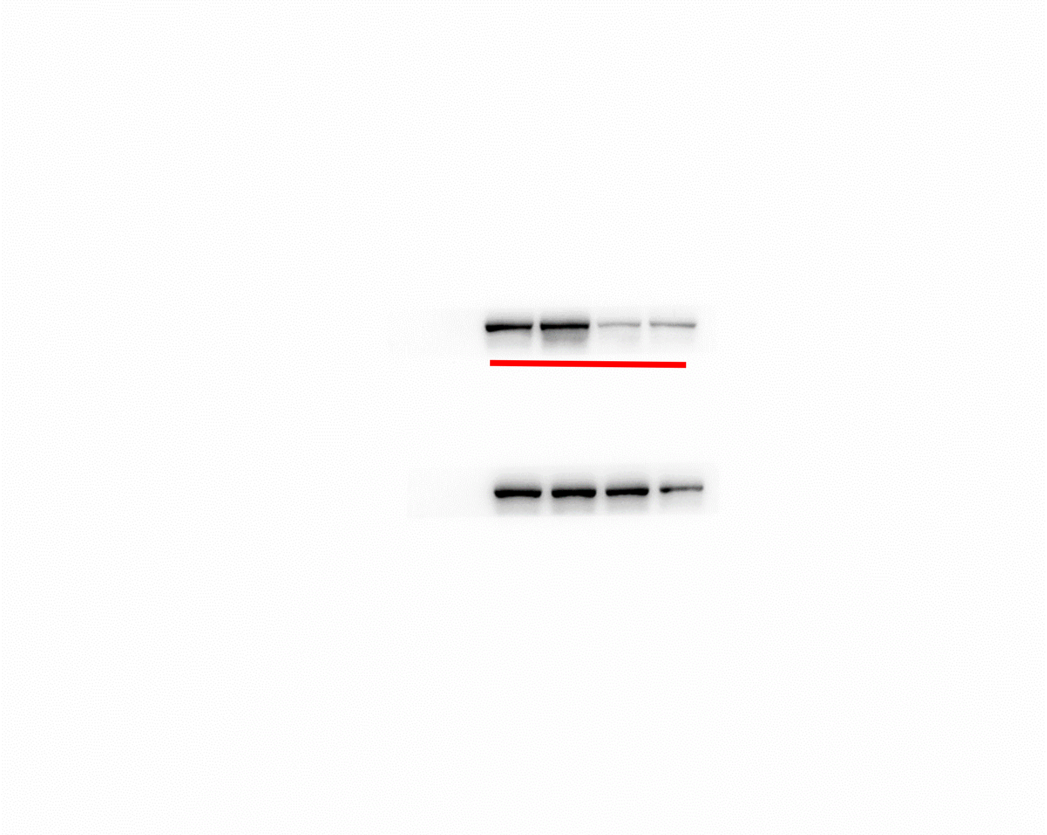

Fig 6A H1299 DHX9

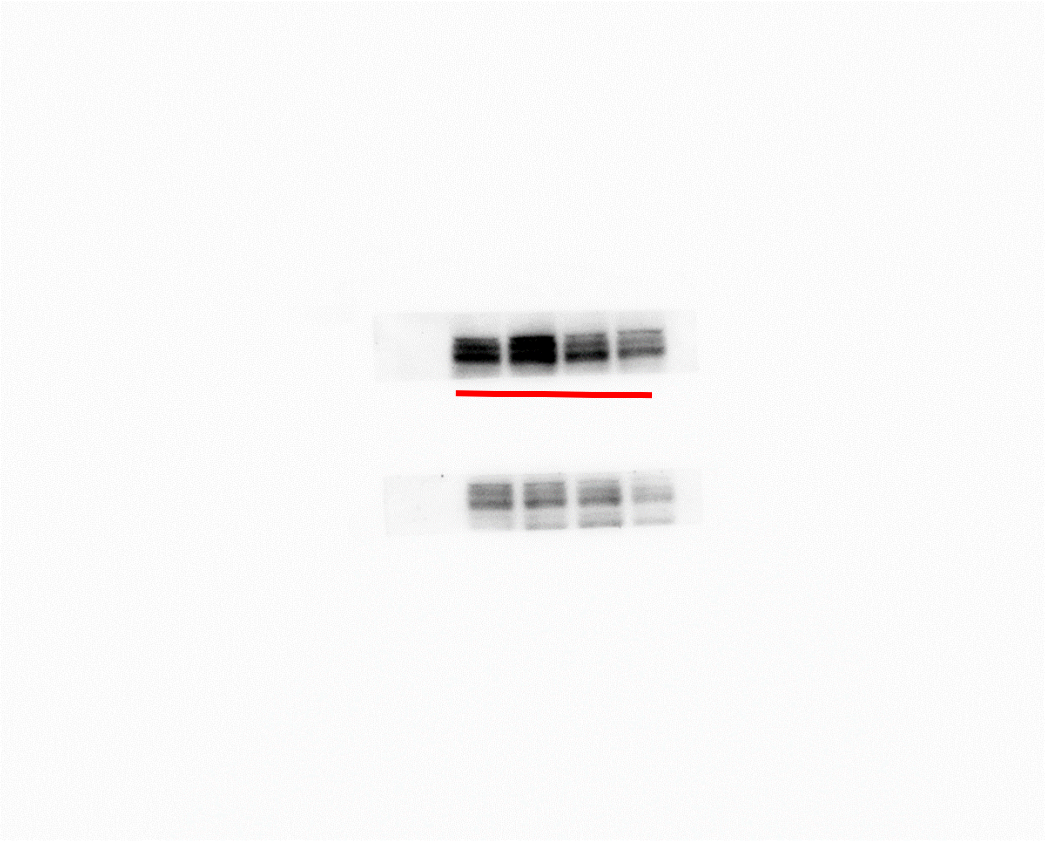

Fig 6A H1299 FOXM1

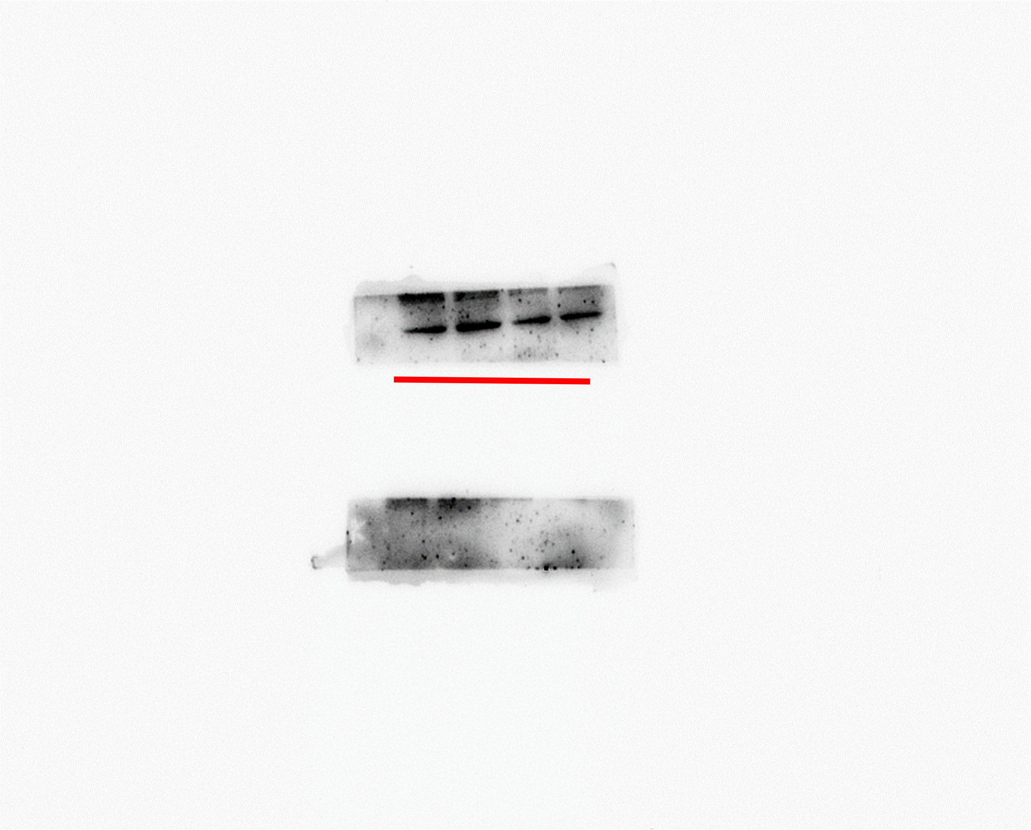

Fig 6A H1299 KIF20A

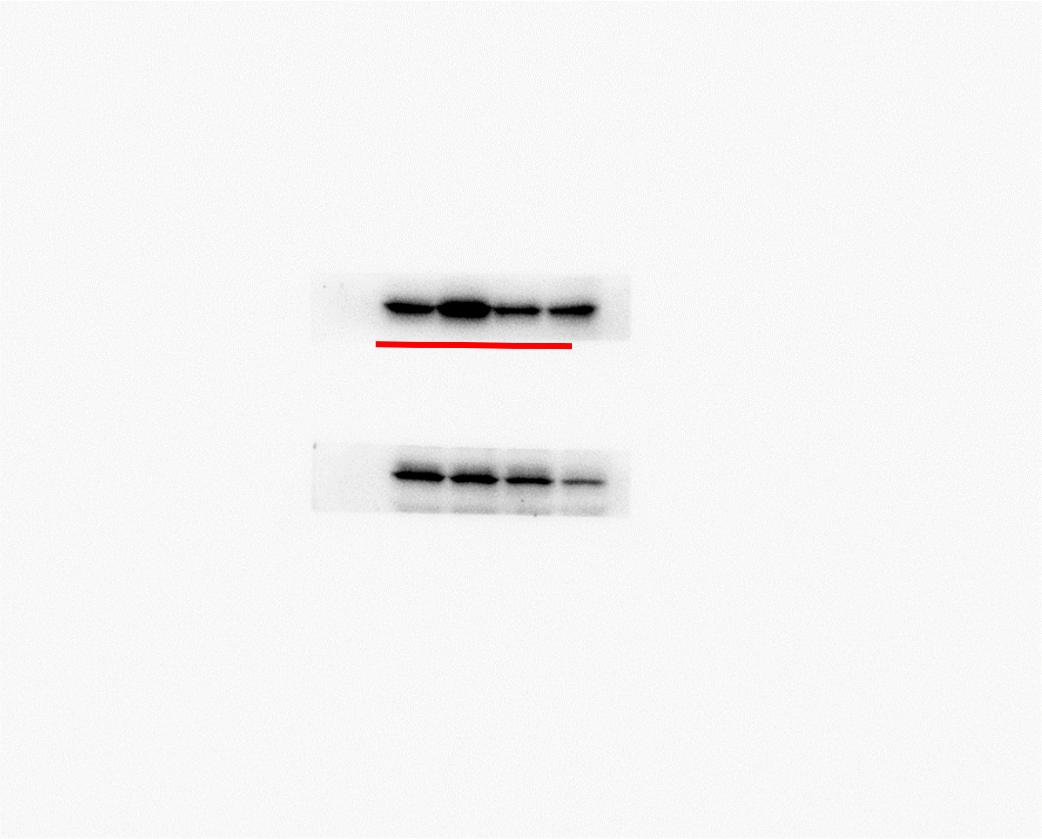

Fig 6A H1299 CCNB1

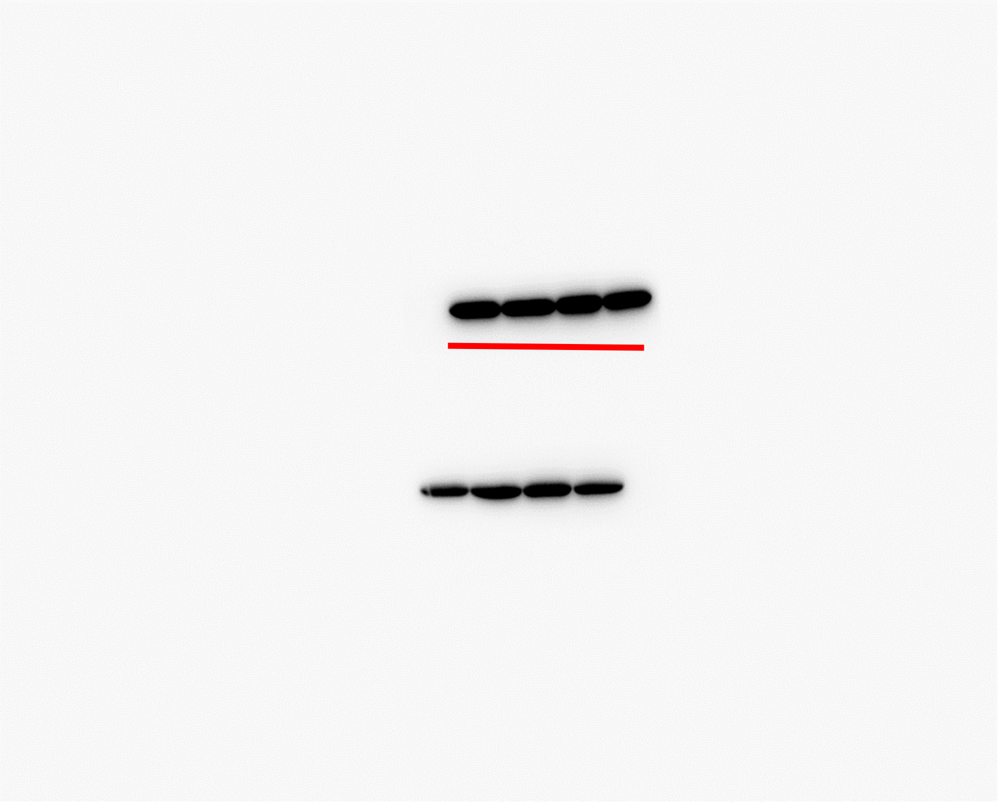

Fig 6A H1299 TUBULIN

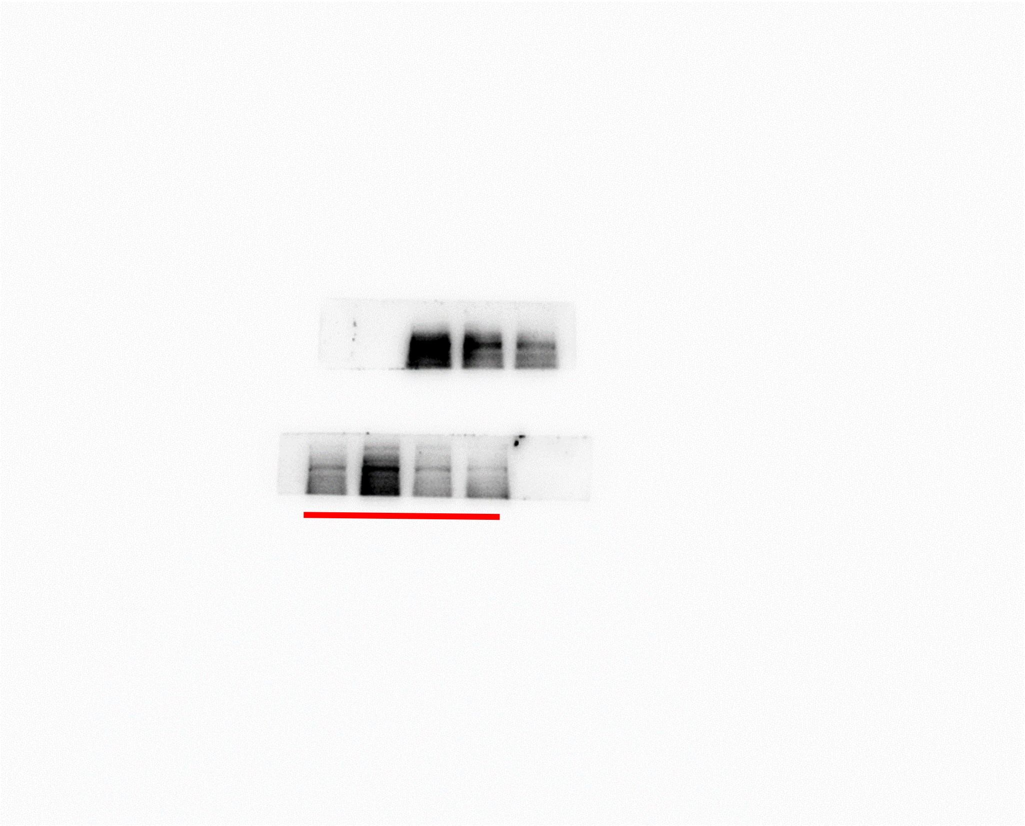

Fig 6A A549 CENPF

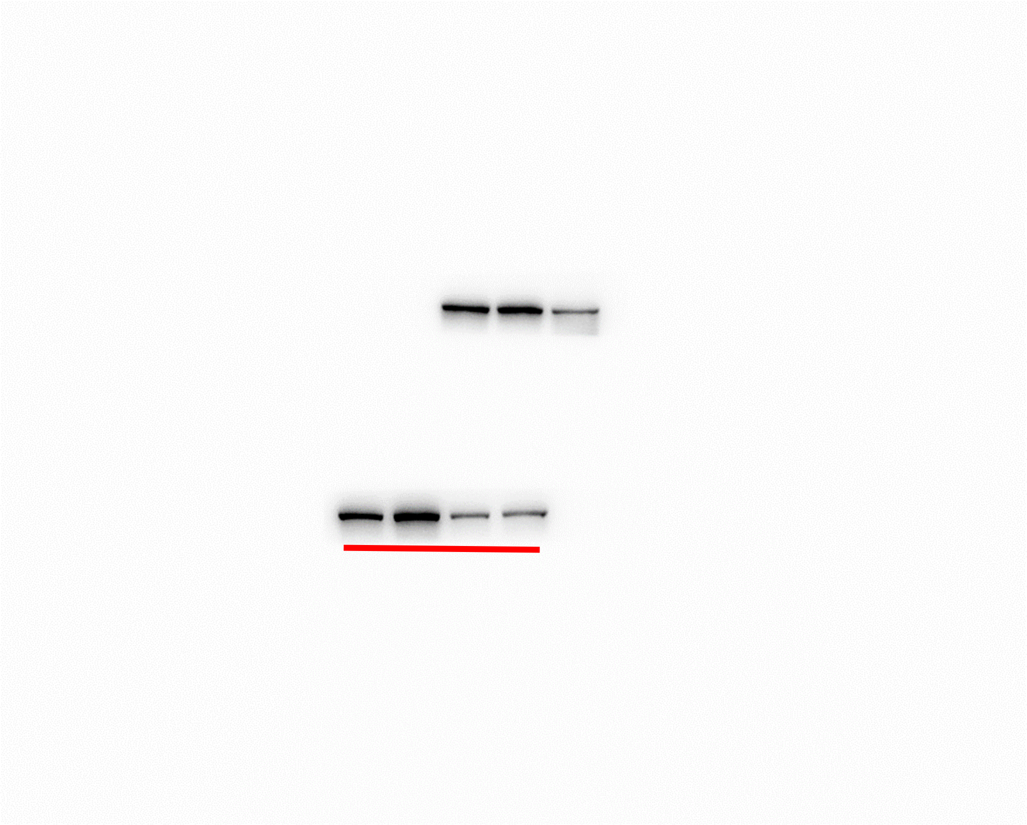

Fig 6A A549 DHX9

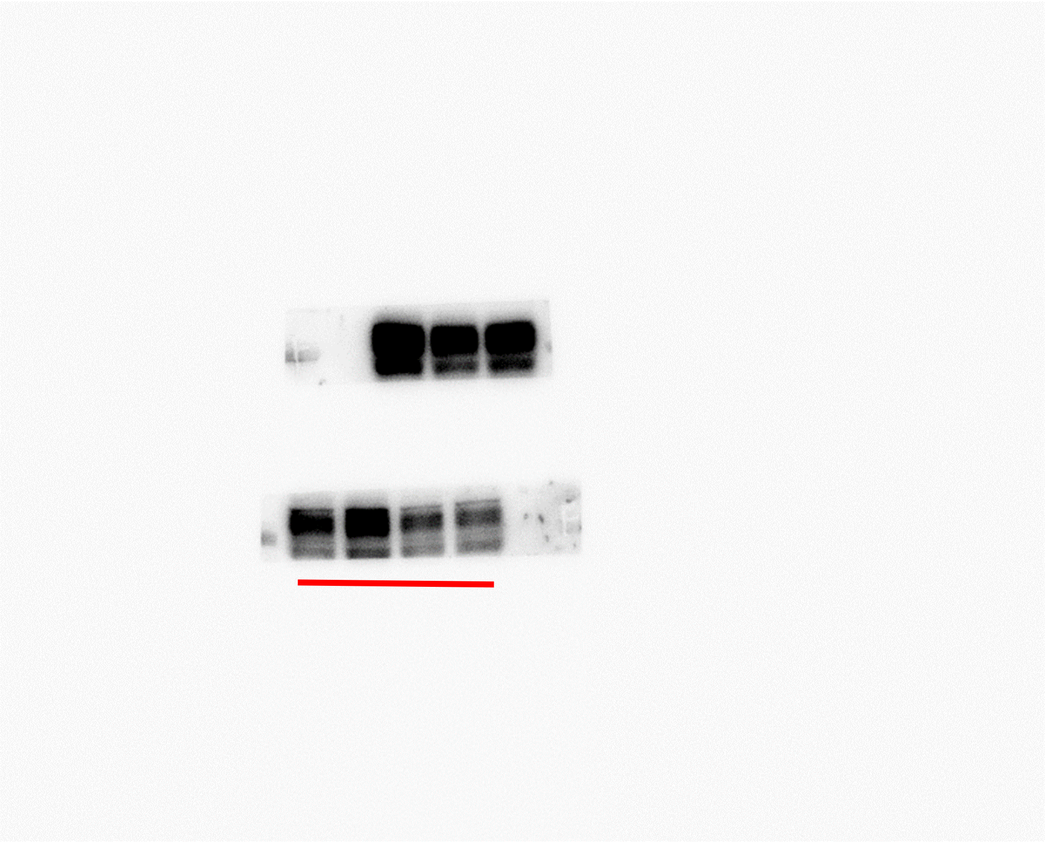

Fig 6A A549 FOXM1

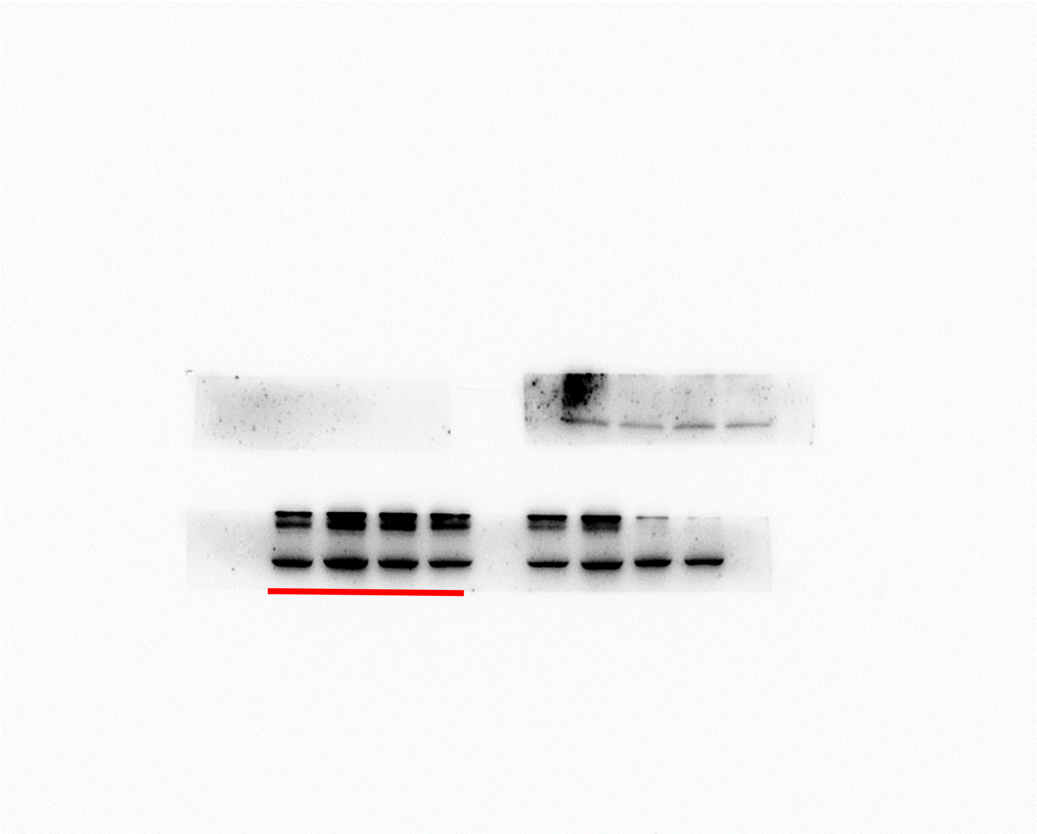

Fig 6A A549 KIF20A

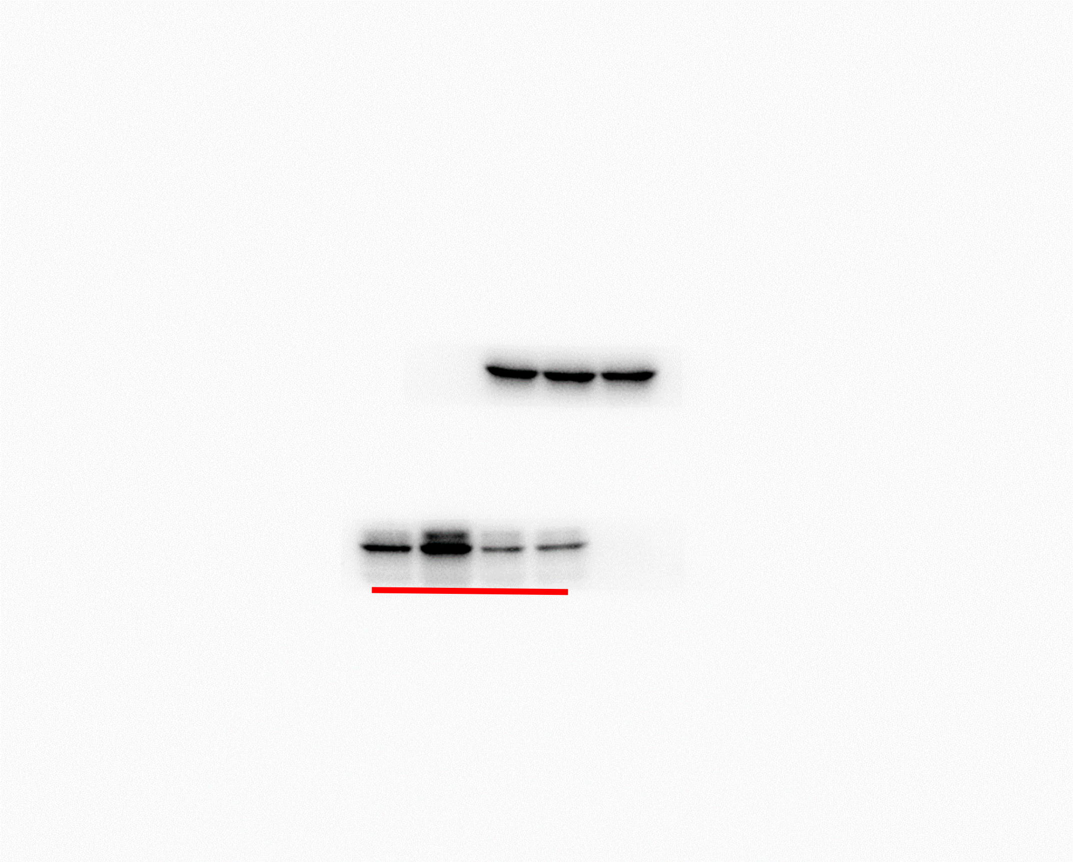

Fig 6A A549 CCNB1

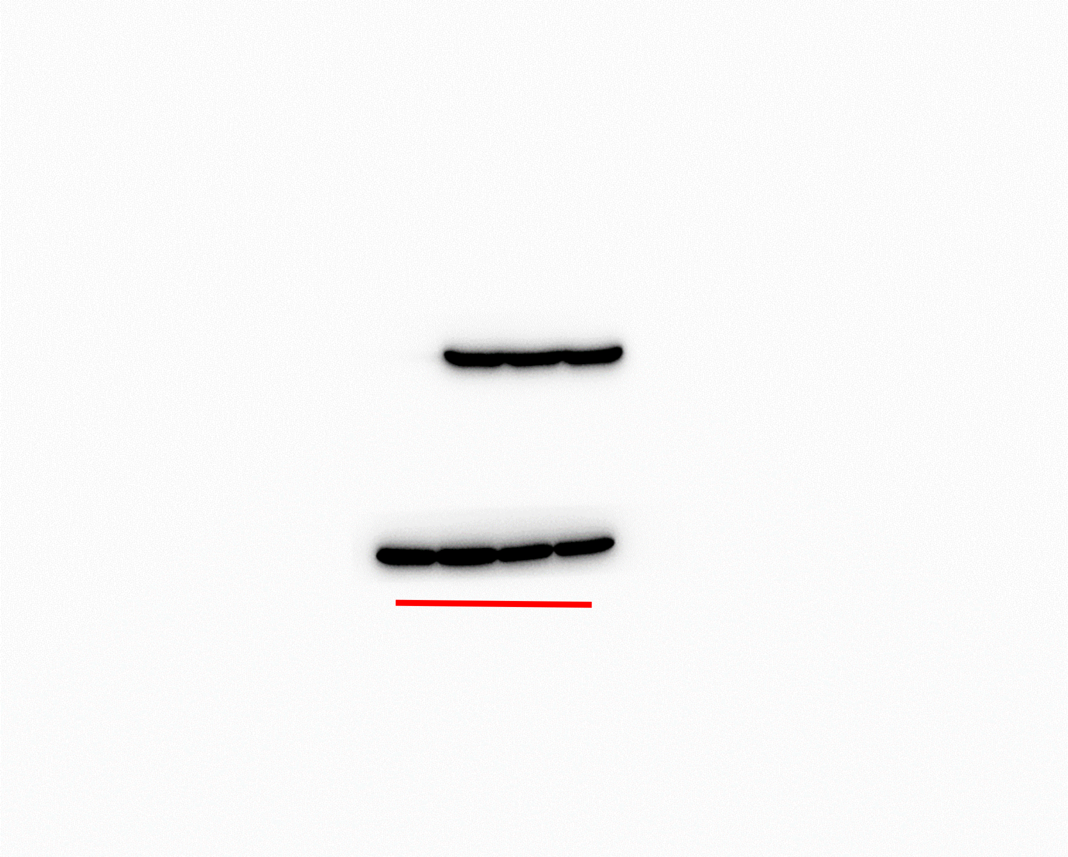

Fig 6A A549 TUBULIN

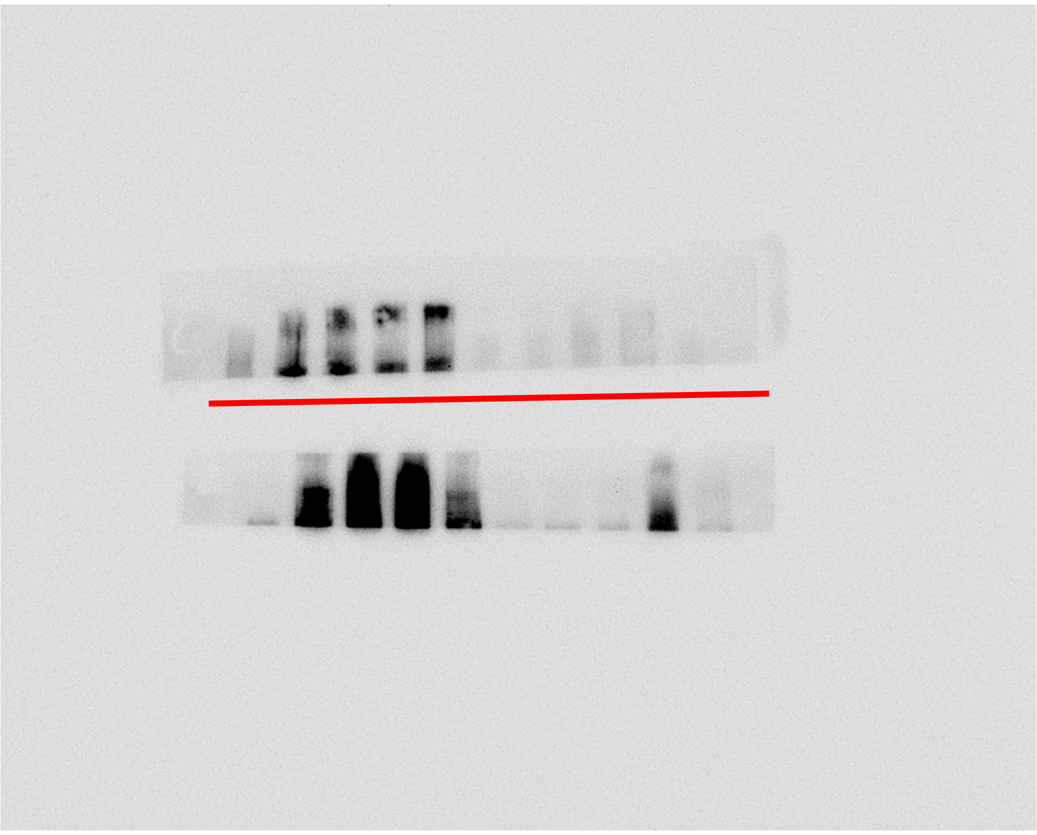

Fig 7D CENPF

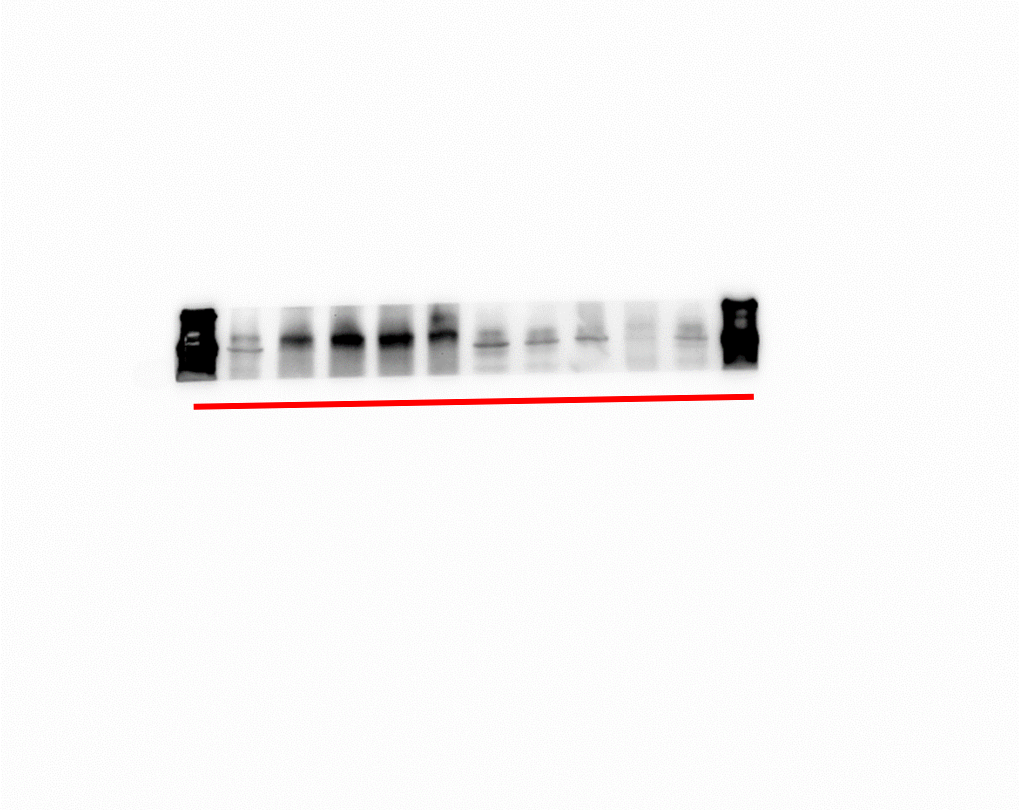

Fig 7D FOXM1

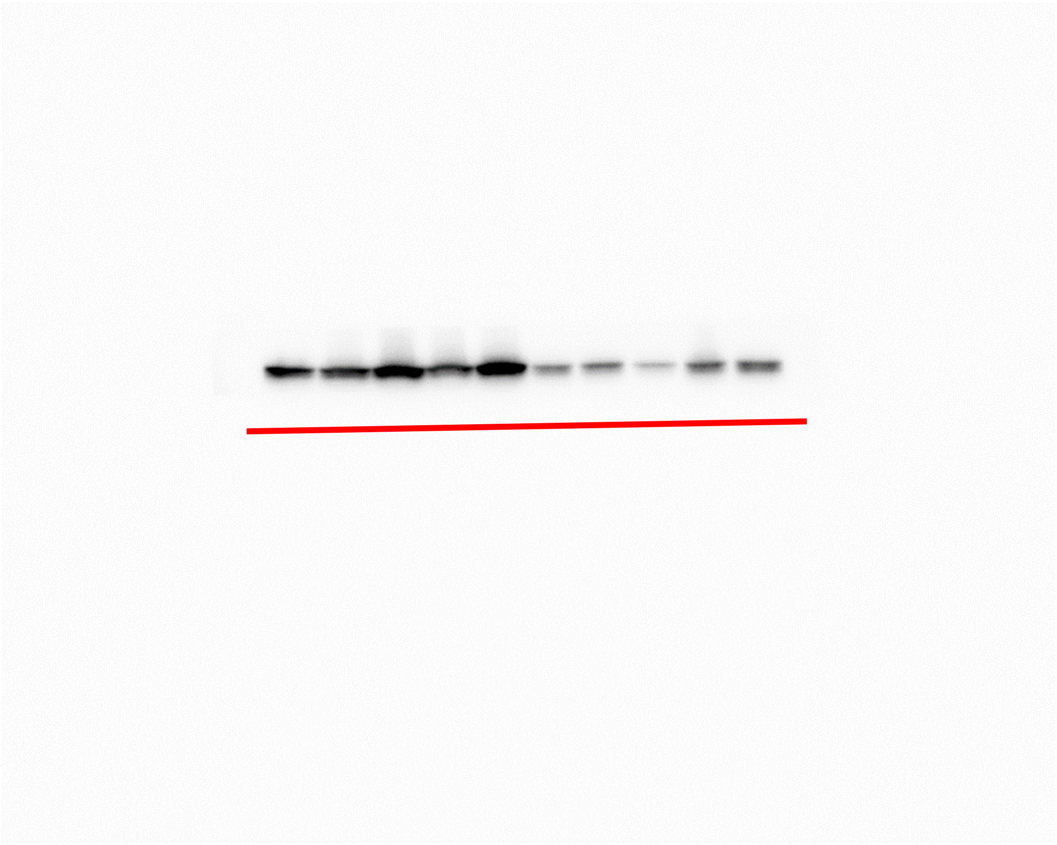

Fig 7D CCNB1

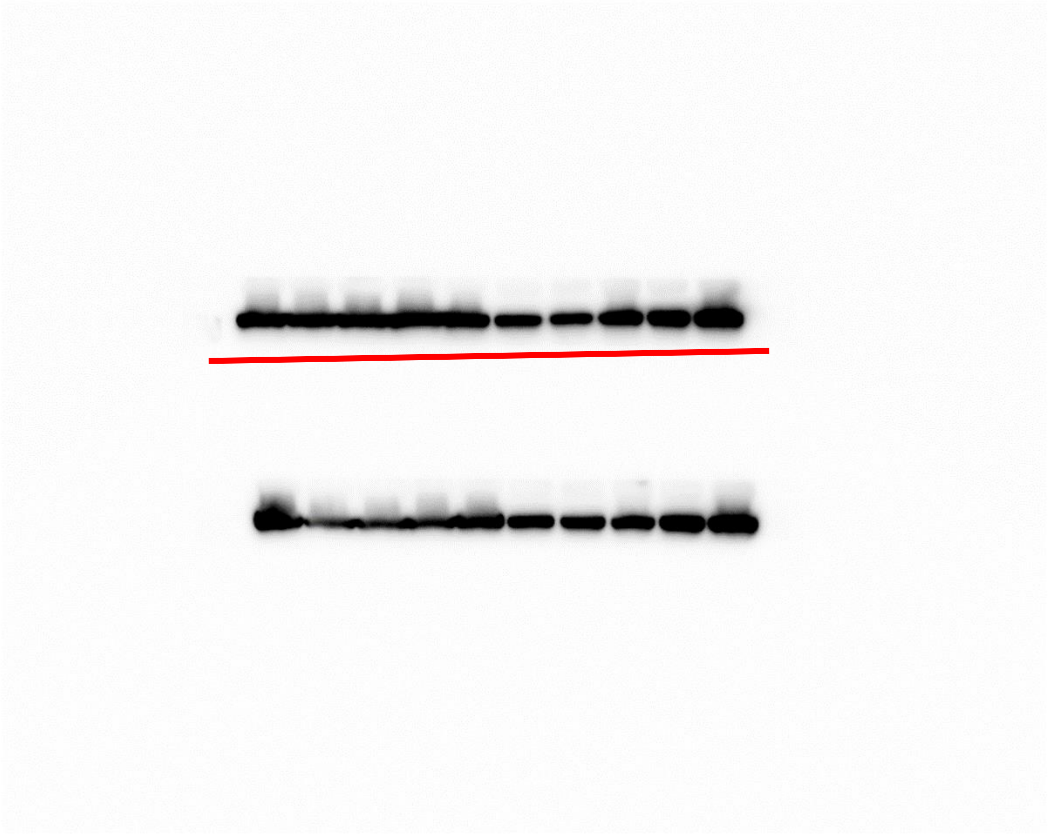

Fig 7D TUBULIN
